# Activation of H–H, HO–H, C(sp^2^)–H, C(sp^3^)–H, and RO–H Bonds by Transition-Metal Frustrated Lewis Pairs Based on M/N (M = Rh, Ir) Couples

**DOI:** 10.1021/acs.inorgchem.2c01902

**Published:** 2022-08-10

**Authors:** María Carmona, Roberto Pérez, Joaquina Ferrer, Ricardo Rodríguez, Vincenzo Passarelli, Fernando J. Lahoz, Pilar García-Orduña, Daniel Carmona

**Affiliations:** Departamento de Química Inorgánica, Instituto de Síntesis Química y Catálisis Homogénea (ISQCH), CSIC - Universidad de Zaragoza, Pedro Cerbuna 12, 50009 Zaragoza, Spain

## Abstract

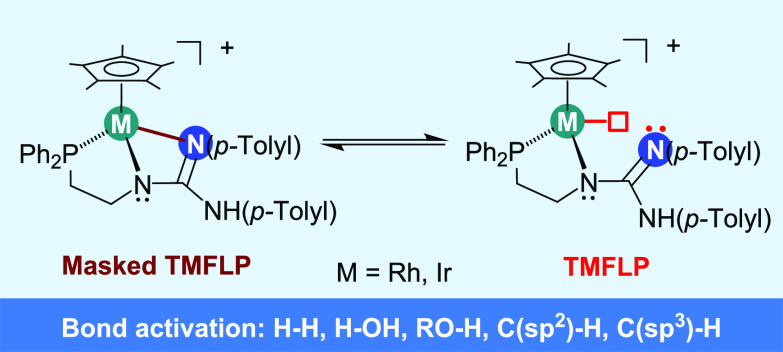

Reaction of the dimers [(Cp*MCl)_2_(μ-Cl)_2_] (Cp* = η^5^-C_5_Me_5_) with Ph_2_PCH_2_CH_2_NC(NH(*p*-Tolyl))_2_ (**H_2_L**) in the presence of NaSbF_6_ affords the chlorido complexes [Cp*MCl(κ^2^*N*,*P*-**H_2_L**)][SbF_6_] (M = Rh, **1**; Ir, **2**). Upon treatment with aqueous NaOH, solutions of **1** and **2** yield the corresponding complexes [Cp*M(κ^3^*N*,*N′*,*P*-**HL**)][SbF_6_] (M = Rh, **3**; Ir, **4**) in which the ligand **HL** presents a *fac* κ^3^*N*,*N′*,*P* coordination mode. Treatment of THF solutions of complexes **3** and **4** with hydrogen gas, at room temperature, results in the formation of the metal hydrido-complexes [Cp*MH(κ^2^*N*,*P*-**H_2_L**)][SbF_6_] (M = Rh, **5**; Ir, **6**) in which the N(*p*-Tolyl) group has been protonated. Complexes **3** and **4** react with deuterated water in a reversible fashion resulting in the gradual deuteration of the Cp* group. Heating at 383 K THF/H_2_O solutions of the complexes **3** and **4** affords the orthometalated complexes [Cp*M(κ^3^*C*,*N*,*P*-**H_2_L_-H_**)][SbF_6_] [M = Rh, **7**; Ir, **8**, **H_2_L_-H_** = Ph_2_PCH_2_CH_2_NC(NH(*p*-Tolyl))(NH(4-C_6_H_3_Me))], respectively. At 333 K, complexes **3** and **4** react in THF with methanol, primary alcohols, or 2-propanol giving the metal-hydrido complexes **5** and **6**, respectively. The reaction involves the acceptorless dehydrogenation of the alcohols at a relatively low temperature, without the assistance of an external base. The new complexes have been characterized by the usual analytical and spectroscopic methods including the X-ray diffraction determination of the crystal structures of complexes **1**–**5**, **7**, and **8**. Notably, the chlorido complexes **1** and **2** crystallize both as enantiopure conglomerates and as racemates. Reaction mechanisms are proposed based on stoichiometric reactions, nuclear magnetic resonance studies, and X-ray crystallography as well as density functional theory calculations.

## Abstract 2

In solution, masked transition-metal frustrated Lewis pairs (TMFLPs) give rise to the corresponding TMFLP species which activate dihydrogen, water, and alcohols following FLP reaction pathways. When D_2_O or alcohols with deuterated OH groups were employed, H/D exchange at the Cp* ligand (involving C(sp^3^)−H activation) was observed. C(sp^2^)−H bond activation involving orthometalation of the *p*-Tolyl ring was also observed.

## Introduction

In 2006, Stephan’s group reported that the phosphano-borane compound (C_6_H_2_Me_3_)_2_P(C_6_F_4_)B(C_6_F_5_)_2_ reacted reversibly with molecular hydrogen to give the phosphonium-borate species (C_6_H_2_Me_3_)_2_PH(C_6_F_4_)BH(C_6_F_5_)_2_. This reaction demonstrates that compounds of representative elements are capable of activating the dihydrogen molecule breaking the paradigm that hydrogen activation is an exclusive ability of transition-metal compounds.^1^ This novel reactivity is based on the cooperative behavior of an acidic (electron acceptor, boron) and a basic (electron donor, phosphorus) component that cannot form dative bonds due to geometry constrains. To highlight this feature, the term “frustrated Lewis pair” (FLP) was coined.^2^

Shortly afterward, the assortment of acidic and basic components was significantly expanded, and it was demonstrated that the resulting FLPs were capable of activating a variety of substrates including imines, olefins, alkynes, organic carbonyl compounds, carbon dioxide, azides, or nitric oxide. Subsequently, the FLP chemistry advanced by incorporating unusual stoichiometric reactions as well as catalytic processes such as hydrogenation (including enantioselective hydrogenation), hydrosilylation, hydroboration, or hydroamination.^3^

In addition, the potential of FLP systems increased considerably with the proposal of Wass’s group to incorporate components based on transition metals in their design, resulting in the so-called transition-metal frustrated Lewis pairs (TMFLPs).^4^ The incorporation of transition-metal fragments into FLP systems increases their structural and electronic diversity in such a way that it should allow them to efficiently promote the whole set of elementary reactions characteristic of catalytic processes. In this regard, Wass^5^ and Erker’s^6^ groups developed extensively the FLP chemistry of Zr/P systems and demonstrated their potential in the activation of small molecules as well as in catalysis. The area was quickly extended to new TMFLPs with various transition metals including bimetallic FLPs.^7^ In this context, it should be noted that the reactivity of TMFLP species can be framed in the broader field of metal–ligand cooperation.^8^

A further qualitative leap in the area of FLP systems occurred when it was discovered that some combinations of Lewis acids and Lewis bases exhibited FLP reactivity despite the fact that the formation of the corresponding classical Lewis adduct (CLA) was observed.^9^ In this regard, it was established that in order for the system to exhibit FLP behavior it is enough that an equilibrium exists between the CLA form and the dissociated form, that is, that the dissociated form is thermally accessible.^10^ To describe this type of system, the concept of “thermally induced frustration” was introduced^11^ and the terms “masked”^12^ and “dormant”^13^ have been used to refer to the involved FLPs.

The activation of the O–H bond of water is one of the steps in the search for efficient catalysts for water splitting on the route to renewable energy generation.^14^ Among the strategies employed to this end, metal–ligand cooperative chemistry^14g,15^ and FPLs, based on both representative elements^16^ and transition-metal components,^7d,7^ have been successfully applied. On the other hand, the Cp* ligand forms robust complexes with a large variety of elements of the periodic table and, usually, it is a nonreactive ligand. However, rare examples of cooperative metal–ligand reactivity involving this ligand have been reported. Indeed, hydrogen abstraction from Cp* methyls has been accomplished either by treatment with an external strong base^17^ or through an intramolecular pathway by means of a basic ligand.^18^ The C–H bond cleavage usually leads to tetramethylfulvene complexes in which the fulvene moiety may display distinct coordination modes.^17b−17d^ When this activation was coupled with the activation of the O–D bond of deuterated water, in some instances, a very unusual H/D exchange of the Cp* methyl protons was observed.^17e,18^

The field of metal–ligand cooperation also includes some of the acceptorless alcohol dehydrogenation (AAD) processes catalyzed by metallic compounds. AAD is a dehydrogenative oxidation process with important applications in energy, green chemistry, and organic synthetic methods. Successful cases of AAD include the use of a variety of transition-metal complexes containing chelates, pincers, and related multidentate ligands as catalysts.^19^ Some of the ligands possess a basic site able to abstract a proton from the alcohol, and the resulting alkoxide transfers a hydride from the α-CH position to the metal directly or via β-elimination.^19g,20^

**Chart 1:**
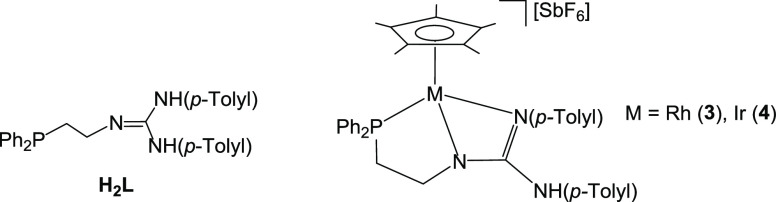
The Ligand **H_2_L** and the Complexes [Cp*M(κ^3^*N*,*N′*,*P*-**HL**)][SbF_6_]

With these concerns in mind, in the present article, we report: (i) the preparation and characterization of the masked TMFLP compounds [Cp*M(κ^3^*N*,*N′*,*P*-**HL**)][SbF_6_] (Cp* = η^5^-C_5_Me_5_; **H_2_L** = *N*,*N′*-bis(*p*-Tolyl)-*N″*-(2-diphenylphosphanoethyl)guanidine; M = Rh, **3**; Ir, **4**; Chart 1); (ii) the reactivity of these complexes with H_2_ and H_2_O; (iii) the hydrogen abstraction from Cp* methyls in complexes **3** and **4** that results in an H/D gradual exchange when deuterated reagents were employed; (iv) the orthometalation reaction of one *p*-Tolyl ring of the phosphano-guanidine ligand, and (v) the acceptorless dehydrogenation of alcohols promoted by **3** and **4**.

Part of this work has been previously communicated.^21^ Herein, we extend our study to the iridium homologue complex **4**. Moreover, the reaction of complexes **3** and **4** with alcohols as well as orthometalation reactions, involving new C(sp^3^)–H, O–H and C(sp^2^)–H activations, is also included.

## Results and Discussion

### Preparation of the Complexes [Cp*MCl(κ^2^*N*,*P*-H_2_L)][SbF_6_] (M = Rh, 1; Ir, 2)

Reaction of the dimers [(Cp*MCl)_2_(μ-Cl)_2_]^22^ with the phosphano-guanidine compound **H_2_L**(21) in the presence of NaSbF_6_ affords the chlorido complexes [Cp*MCl(κ^2^*N*,*P*-**H_2_L**)][SbF_6_] (M = Rh, **1**; Ir, **2**; eq 1).
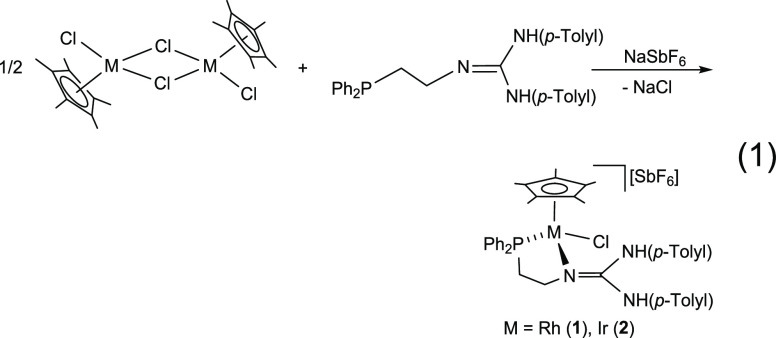
1

Compounds **1** and **2** were characterized by analytical and spectroscopic methods (see the Supporting Information) and by the X-ray determination of their crystal structures. The κ^2^*N*,*P* coordination of the **H_2_L** ligand renders the metal atom a stereogenic center. Consequently, the methylene protons of the phosphano-guanidine ligand are diastereotopic, and in the proton nuclear magnetic resonance (^1^H NMR) spectrum, they give four distinct resonances. Broad bands in the 3000–3400 cm^–1^ region of the IR spectra together with ^1^H NMR singlets at 9.24 and 7.34 ppm (**1**) and 8.93 and 7.16 ppm (**2**) are indicative of the presence of two nonequivalent NH groups in the molecule. The ^31^P{^1^H} NMR spectrum consists of a doublet centered at 51.37 ppm for the rhodium complex [*J*(RhP) = 142.4 Hz] and a singlet at 26.52 ppm for the iridium compound, proving the coordination of the phosphorus to the metal (δP free ligand: −21.14 ppm).

Slow evaporation of saturated solutions of **1** and **2** in CH_2_Cl_2_/Et_2_O/*n*-pentane mixtures gave rise to the simultaneous formation of single crystals of pure enantiomers (conglomerates^23^) and racemates, for both compounds. Enantiopure samples of **1** and **2** slowly racemize in solution. Thus, for example, starting from a dichloromethane solution of pure *S*_Rh_-**1**, *S*_Rh_-**1**/*R*_Rh_-**1** molar ratios of about 92/8 and 74/26 were measured, by circular dichroism (CD) spectroscopy, after 2 and 18 h at room temperature, respectively.

**Figure 1:**
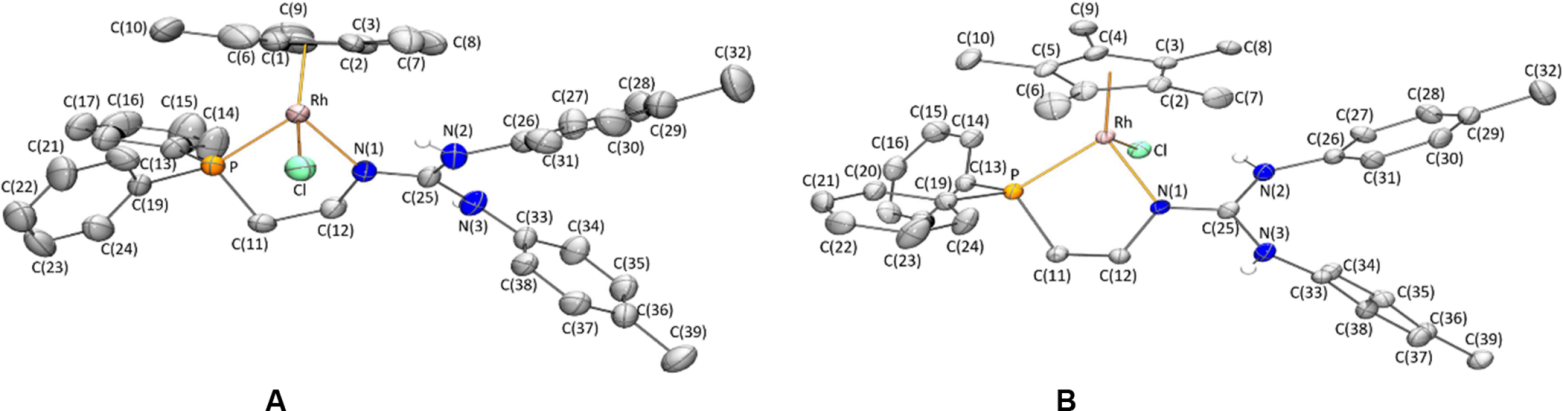
View of the cations of the *R*_Rh_ (A) and *S*_Rh_ (B) enantiomers of the rhodium complex [Cp*RhCl(κ^2^*N*,*P*-**H_2_L**)][SbF_6_] (**1**). For clarity, hydrogen atoms (except those bonded to nitrogen atoms) have been omitted.

**Figure 2:**
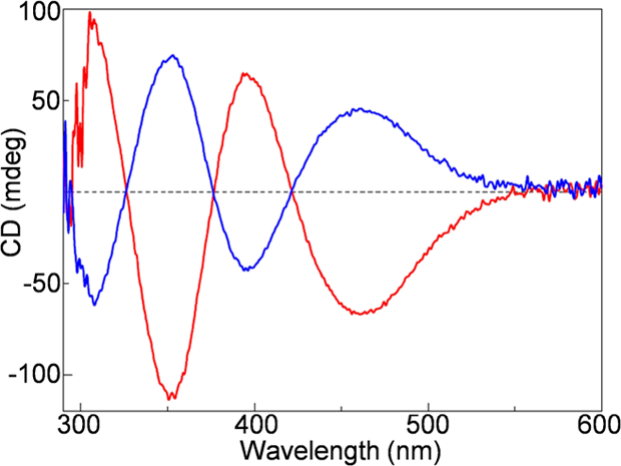
CD spectra of *S*_Rh_-**1** (blue) and *R*_Rh_-**1** (red) in CH_2_Cl_2_.

A view of the cation of both enantiomers of the rhodium complex **1** is depicted in Figure 1. Views of the *rac*-**1** cations as well as of the cation of the iridium enantiomers *R*_Ir_-**2**, *S*_Ir_-**2** and racemate *rac*-**2** are included in the Supporting Information. Figure 2 shows the enantiomorphic relationship of the CD spectra of the two enantiomers of the rhodium complex **1**.^24^

**Figure 3:**
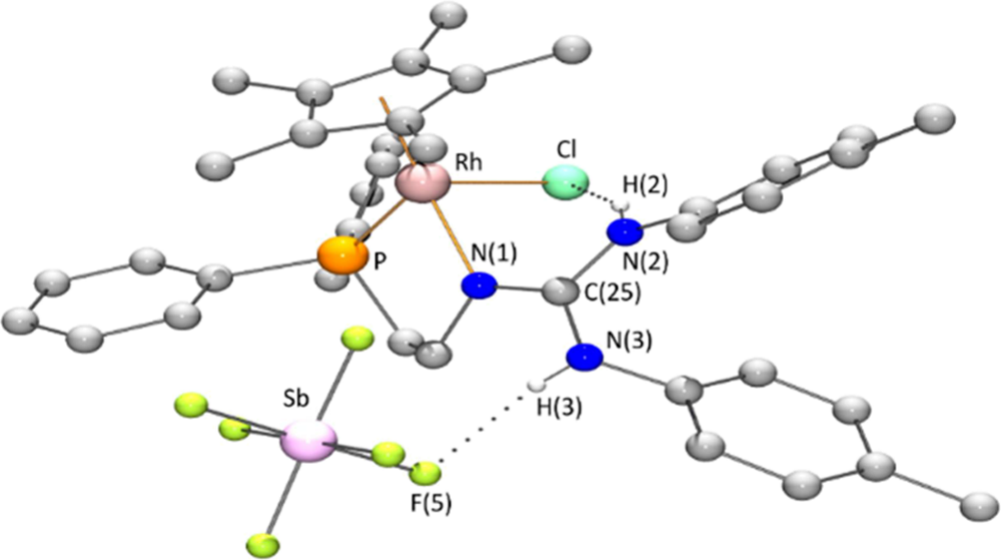
H-bond interactions in complex *S*_Rh_-**1**. For clarity, only hydrogen atoms of N–H fragments have been depicted.

Table S1 (Supporting Information) collects the most relevant structural parameters for the cations of *R*_Rh_-**1**, *rac*-**1**, *R*_Ir_-**2**, *S*_Ir_-**2**, and *rac*-**2**, comparable structural parameters being observed regardless of the configuration and the nature of the metal center. Hence, only the structural parameters found in the *S*_Rh_-**1** isomer will be discussed. Selected bond lengths and angles of the cation of *S*_Rh_-**1** are summarized in Table 1. The cation of this complex exhibits “three-legged piano-stool” geometry. An η^5^-C_5_Me_5_ group occupies three *fac* positions, and the κ^2^*N*,*P* chelating phosphano-guanidine ligand and a chlorine atom complete the coordination sphere of the metal. The absolute configuration of the metal center is *S*, according to the atom priority sequence *η^5^*-C_5_Me_5_ > Cl > P > N.^25^ The structural parameters of the CN_3_ guanidine moiety deserve some comments. The C–NH(*p*-Tolyl) bond distances, N(2)–C(25) 1.344(8), N(3)–C(25) 1.369(7) Å, indicate a slight partial double bond character for these bonds,^26^ while the N(1)–C(25) bond distance, involving the nitrogen coordinated to the metal atom, is found to be comparatively shorter, 1.310(7) Å, but also longer than typical N=C bond lengths (1.279(8) Å).^26^ The sum of the bond angles at the coordinated nitrogen is 359.6(7)° indicating that the C(12)N(1)Rh(1)C(25) fragment is essentially planar. Hydrogen bonds between the N(2)–H(2) proton and the chlorido ligand [N–H = 0.82(7) Å, H···Cl = 2.45(7) Å, N···Cl = 3.203(5) Å, N–H···Cl = 153(7)°] and between the N(3)–H(3) proton and one of the fluorine atoms of the SbF_6_ anion [N–H = 0.82(8) Å, H···F = 2.13(8) Å, N···F = 2.939(7) Å, N–H···F = 169(7)°] were observed (Figure 3).

**Table 1:** Selected Bonds Lengths (Å) and Angles (°) for Complex *S*_**Rh**_-**1**

Rh–Cl	2.4199(14)	Cl-Rh–Ct^a^	121.8(1)
Rh–P	2.2916(15)	P–Rh–N(1)	83.31(13)
Rh–N(1)	2.123(5)	P–Rh–Ct^a^	130.4(2)
Rh–Ct^a^	1.8218(1)	N(1)–Rh–Ct^a^	131.2(2)
N(1)–C(25)	1.310(7)	Rh–N(1)–C(12)	118.3(3)
N(2)–C(25)	1.344(8)	Rh–N(1)–C(25)	122.8(4)
N(3)–C(25)	1.369(7)	C(12)–N(1)–C(25)	118.5(5)
Cl–Rh–P	90.54(5)	Σ°N(1)^b^	359.6(7)
Cl–Rh–N(1)	85.15(14)		

a Ct represents the centroid of the η^5^-C_5_Me_5_ ligand.

b Σ°N(1) is the sum of bond angles around N(1) atom.

### Preparation of the Complexes [Cp*M(κ^3^*N*,*N′*,*P*-HL)][SbF_6_] (M = Rh, 3; Ir, 4)

Solutions of **1** and **2** in 1:1 (v/v) THF/toluene were treated with aqueous NaOH for 1.5 h affording the corresponding complexes [Cp*M(κ^3^*N*,*N′*,*P*-**HL**)][SbF_6_] (M = Rh, **3**; Ir, **4**) through base-induced elimination of HCl and subsequent coordination of the deprotonated nitrogen (eq 2).
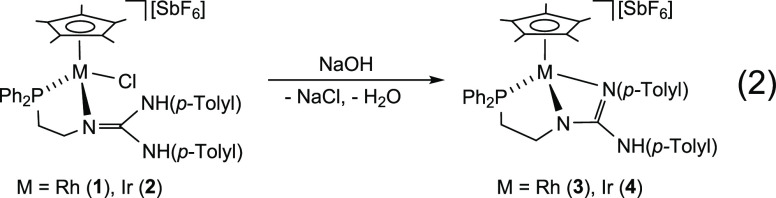
2

The compounds were characterized by analytical and spectroscopic methods (see the Supporting Information) and by the X-ray diffraction determination of their crystal structures. A weak IR band at 3377 and 3362 cm^–1^ for **3** and **4**, respectively, and a broad singlet in the proton NMR spectrum at 7.89 (**3**) and 8.00 ppm (**4**) are attributed to the NH functionality. As a consequence of the stereogenicity at the metal, the PCH_2_CH_2_N methylene protons are asynchronous and give rise to four resonances at the expected chemical shifts and with the awaited multiplicities (see the Supporting Information). A doublet centered at 48.27 ppm [*J*(RhP) = 159.0 Hz] and a singlet at 27.75 ppm in the ^31^P{^1^H} NMR spectrum are assigned to the phosphorus nucleus of the PPh_2_ group of the phosphano-guanidine ligand.

**Figure 4:**
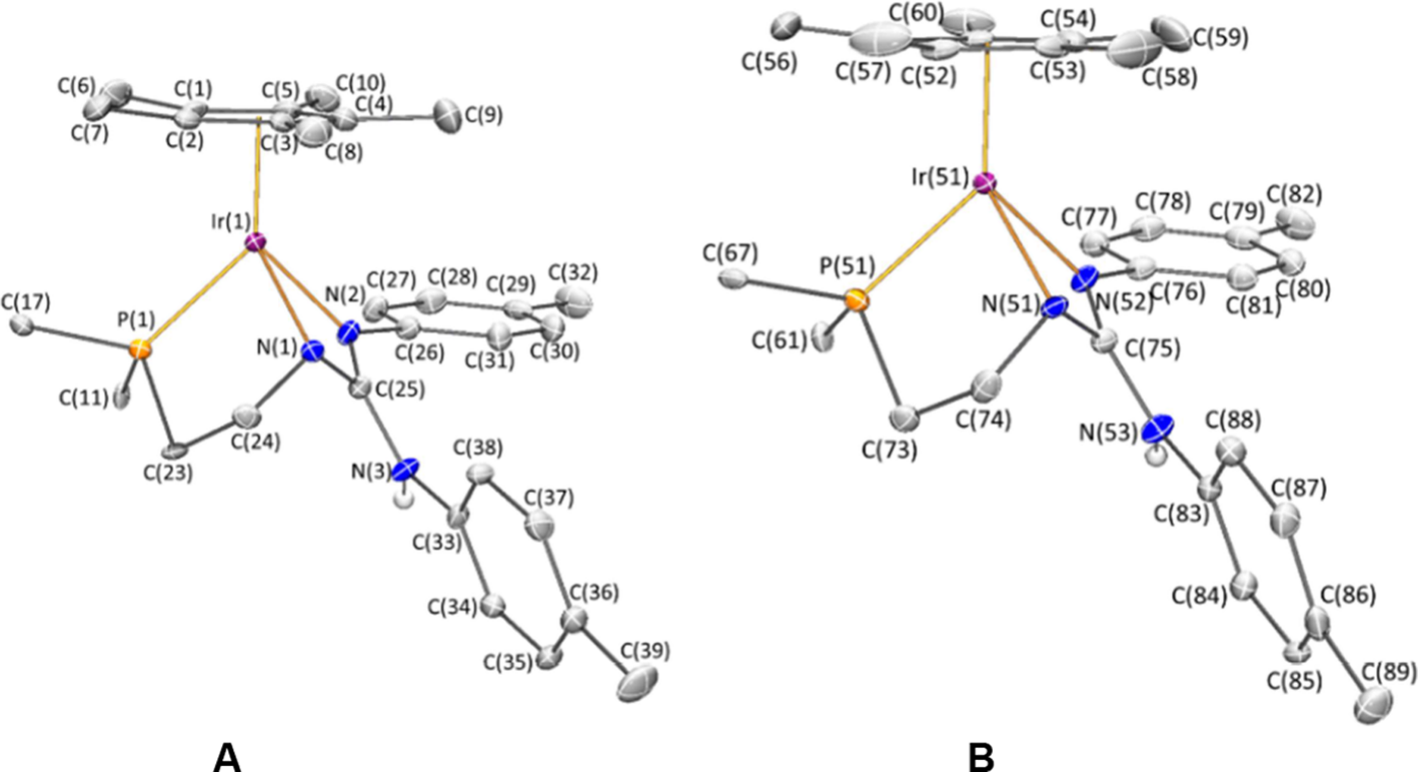
View of the two independent molecules of the cation of complex *R*_M_,*S*_N_-**4**. For clarity, only the *ipso* carbon of the phenyl rings of the PPh_2_ group is shown, and hydrogen atoms (except the NH proton) have been omitted.

The molecular structure of **3** and **4** has been determined by X-ray diffraction means. There is no significant chemical difference to be remarked when comparing the structural parameters of the cations of the two complexes. For more detailed data about the molecular structure of the rhodium complex **3**, see ref (21). Figure 4 shows a view of the two crystallographically independent, but chemically equivalent, cations (**A** and **B**) found in the asymmetric unit of iridium complex **4**. Table 2 collects selected bond lengths and angles of both cations. The molecular structure reveals that the ligand **HL** presents a *fac* κ^3^*N*,*N′*,*P* coordination mode. This type of coordination renders the metal and the central nitrogen atom of the ligand stereogenic. The configuration at metal induces the configuration at nitrogen in such a way that only the *R*_M_,*S*_N_ diastereomer and its *S*_M_,*R*_N_ enantiomer form, both of them being present in the centrosymmetric unit cell of **4·C_4_H_8_O**. In Figure 4, a view of the two independent cations of the *R*_M_,*S*_N_ diastereomer is depicted.

**Table 2:** Selected Bond Lengths (Å) and Angles (°) for the Two Independent Cations of Complex *R*_M_,*S*_N_-**4**

	cation **A**		cation **B**
Ir(1)–P(1)	2.2984(12)	Ir(51)–P(51)	2.2843(12)
Ir(1)–N(1)	2.110(4)	Ir(51)–N(51)	2.124(4)
Ir(1)–N(2)	2.121(4)	Ir(51)–N(52)	2.130(4)
Ir(1)–Ct^a^	1.8276(1)	Ir(51)–Ct^a^	1.8338(1)
N(1)–C(25)	1.357(6)	N(51)–C(75)	1.362(6)
N(2)–C(25)	1.329(5)	N(52)–C(75)	1.324(5)
N(3)–C(25)	1.364(6)	N(53)–C(75)	1.361(6)
P(1)–Ir(1)–N(1)	80.02(11)	P(51)–Ir(51)–N(51)	80.31(11)
P(1)–Ir(1)–N(2)	90.53(11)	P(51)–Ir(51)–N(52)	91.49(11)
P(1)–Ir(1)–Ct^a^	134.64(1)	P(51)–Ir(51)–Ct^a^	133.98(1)
N(1)–Ir(1)–N(2)	62.33(14)	N(51)–Ir(51)–N(52)	62.03(14)
N(1)–Ir(1)–Ct^a^	131.10(1)	N(51)–Ir(51)–Ct^a^	133.21(1)
N(2)–Ir(1)–Ct^a^	130.92(1)	N(52)–Ir(51)–Ct^a^	129.55(1)
Ir(1)–N(1)–C(24)	118.3(3)	Ir(51)–N(51)–C(74)	118.5(3)
Ir(1)–N(1)–C(25)	93.9(3)	Ir(51)–N(51)–C(75)	93.8(3)
C(25)–N(1)–C(24)	116.6(4)	C(75)–N(51)–C(74)	114.9(4)
Σ°N(1)^b^	328.8(6)	Σ°N(51)^b^	327.2(6)
Ir(1)–N(2)–C(25)	94.3(3)	Ir(51)–N(52)–C(75)	94.7(3)
N(1)–C(25)–N(2)	109.2(4)	N(51)–C(75)–N(52)	109.4(4)

a Ct represents the centroid of the *η*^5^-C_5_Me_5_ ligand.

b Σ°N(1) and Σ°N(51) stand for the sum of bond angles around N(1) and N(51) atoms, respectively.

Focusing the discussion on cation **A**, the *fac* κ^3^*N*,*N′*,*P* coordination mode of the **HL** ligand forces the central N(1) atom to adopt a pyramidal geometry [Σ°N(1) = 328.8(6)°]. This geometry together with the N(1)–C(25) bond length [1.357(6) Å] contrasts with the structural features of the corresponding nitrogen atom in the precursor complex **2** where the **H_2_L** ligand coordinates in a chelate κ^2^*N*,*P* manner (for the corresponding parameters of compound **2**, see Table S1, Supporting Information). Remarkably, the bond angles N(1)–Ir(1)–N(2) and N(1)–C(25)–N(2), 62.33(14) and 109.2(4)°, respectively, are far from the hybridization ideal values. All these features will most likely lead to a strong strain within the Ir–N–C–N four-membered metalacycle.

### Reaction of Complexes **3** and **4** with Molecular Hydrogen

The structural parameters found in compounds **3** and **4**, and in particular the envisaged strain within the four-membered metalacycle M–N–C–N led us to hypothesize that these compounds could behave like masked FLPs: the heterolytic cleavage of one of its M–N bonds could generate a TMFLP in which the metal and the nitrogen would play the role of the acid and basic center, respectively. As reported for compound **3**,^21^ these assumptions prompted us to try the reaction of complex **4** with molecular hydrogen.

Indeed, treatment of THF solutions of complexes **3** and **4** with hydrogen gas (4 bar), at room temperature, resulted in the formation of the metal hydrido-complexes [Cp*MH(κ^2^*N*,*P*-**H_2_L**)][SbF_6_] (M = Rh, **5**; Ir, **6**) in which the N(*p*-Tolyl) group has been protonated (eq 3). Formally, the heterolytic breakage of the molecule of hydrogen gives rise to hydridic M–H and protic N–H bonds. Complete conversion to complex **5** was obtained after 4 h of reaction under the above mentioned conditions. Conversion to the iridium complex **6** was complete after 24 h at 373 K. The reaction is reversible but to achieve appreciable dehydrogenation rates it is necessary to heat THF solutions above 373 K. Indeed, heating at 393 K a solution of the hydride **5** for 30 min, a conversion of 30% to the rhodium compound **3** was observed. After heating at the same temperature a solution of the iridium complex **6** for 2.5 h, a conversion of 50% to the dehydrogenated compound **4** was measured.
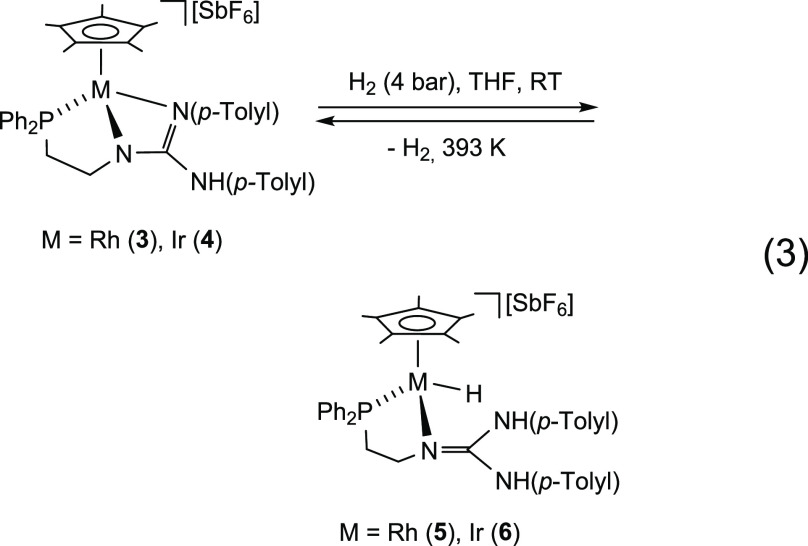
3

A doublet of doublets centered at −10.79 ppm [*J*(PH) = 38.8 Hz, *J*(RhH) 22.6 Hz] for complex **5** and a doublet centered at −10.16 ppm [*J*(PH) = 32.3 Hz] for complex **6** are attributed to the M–H functionality in the cations. The presence of two peaks attributed to NH protons (see the Supporting Information) is indicative of the protonation of the N(*p*-Tolyl) group. The ^31^P{^1^H} NMR spectrum consists of a doublet centered at 61.77 ppm [*J*(RhP) = 143.9 Hz] for the rhodium complex and a singlet at 27.88 ppm for the iridium one.

**Figure 5:**
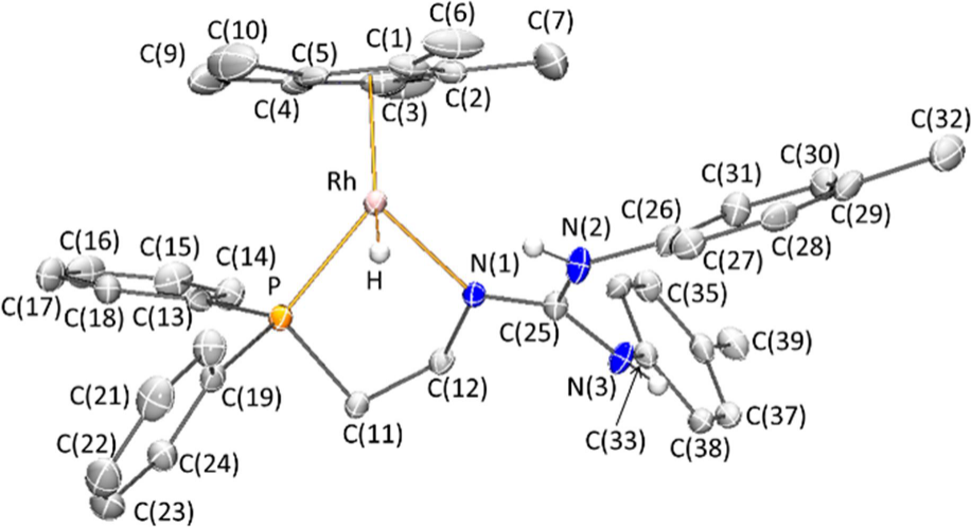
Molecular structure of the cation of complex **5·CD_4_O**. For clarity, hydrogen atoms (except the Rh–H and N–H protons) have been omitted.

The molecular structure of complex **5** corroborates all these features.^21^ The compound crystallizes as a racemate in the *P2_1_/n* space group of the monoclinic system with one solvent molecule in the asymmetric unit (**5·CD_4_O**). The *R*_Rh_ enantiomer is depicted in Figure 5. Selected bond lengths and angles are shown in Table 3. The phosphano-guanidine ligand displays a κ^2^*N*,*P* coordination mode. A Cp* ligand, formally occupying three coordination sites, and a hydrido ligand [Rh–H = 1.56(5) Å] complete the coordination sphere of the metal. The observed RhH···HN (2) separation, 2.20(7) Å, is shorter than twice the hydrogen Van der Waals radius, 2.4 Å, indicating a significant H···H interaction between the protic NH and hydridic RhH functionalities. The structural parameters of the CN_3_ guanidino fragment are comparable to those found for the chlorido compound **1**, that is, a greater double bond character for the CN bond involving the nitrogen atom coordinated to the metal [N(1)–C(25) 1.309(6) Å] when compared with the remaining CN bonds [N(2)–C(25) 1.359(6) Å, N(3)–C(25) 1.369(6) Å] and a planar geometry at the N(1) atom [Σ°N(1) = 358.9(6)°].

**Table 3:** Selected Bond Lengths (Å) and Angles (°) for the Cation of Complex **5·CD_4_O**

Rh–P	2.2419(12)	P–Rh–H	82(2)
Rh–N(1)	2.110(4)	N(1)–Rh–Ct^a^	129.39(1)
Rh–Ct^a^	1.8697(1)	N(1)–Rh–H	88(2)
Rh–H	1.56(5)	Ct^a^–Rh–H	124
N(1)–C(25)	1.309(6)	Rh–N(1)–C(12)	115.9(3)
N(2)–C(25)	1.359(6)	Rh–N(1)–C(25)	124.7(3)
N(3)–C(25)	1.369(6)	C(12)–N(1)–C(25)	118.3(4)
P–Rh–N(1)	82.85(11)	Σ°N(1)^b^	358.9(6)
P–Rh–Ct^a^	134.74(1)		

a Ct represents the centroid of the η^5^-C_5_Me_5_ ligand.

b Σ°N(1) stands for the sum of bond angles around N(1) atom.

Probably, the structural relaxation within the four-membered Ir–N–C–N metalacycle facilitates the reaction from **3** to **5** as well as from **4** to **6**, which, in turn, results in the change in the coordination mode of the phosphano-guanidine ligand from κ^3^*N*,*N′*,*P* to κ^2^*N*,*P* with the concomitant change of the geometry at the N(1) atom from pyramidal to planar.

### Water Activation by Complexes **3** and **4**

As previously reported,^21^ the rhodium complex **3** reacts with deuterated water in a reversible fashion resulting in the gradual deuteration of the Cp* group. At 293 K, ^1^H NMR measurements and mass spectrometry analysis show that deuteration of this group is complete after 15 h in [D_8_]THF/D_2_O (78%/22%, v/v) solution. Also, deuteration was evidenced by the determination of the crystal structure of **3**-*d*_15_ by low-temperature single crystal neutron-diffraction experiments.^21^ During the deuteration process, only isotopologues of compound **3** at different degrees of deuteration are detected by NMR spectroscopy.

Kinetic measurements indicate that the deuteration process obeys a pseudo-first-order rate law with *k*_obs_ values from 3.31 × 10^–6^ to 4.99 × 10^–4^ s^–1^, in the 298–333 K temperature range. The formation of **3**-*d*_15_ from **3** is reversible, and at 313 K, a [D_8_]THF/H_2_O (78%/22%, v/v) solution of **3**-*d*_15_ evolves to **3** with an observed pseudo-first-order rate constant of 3.89 × 10^–5^ s^–1^. The measured ratio *k*_H_/*k*_D_ (2.44) indicates that the rate-determining step for the exchange process is the C–H(D) bond cleavage.^21^

**Scheme 1:**
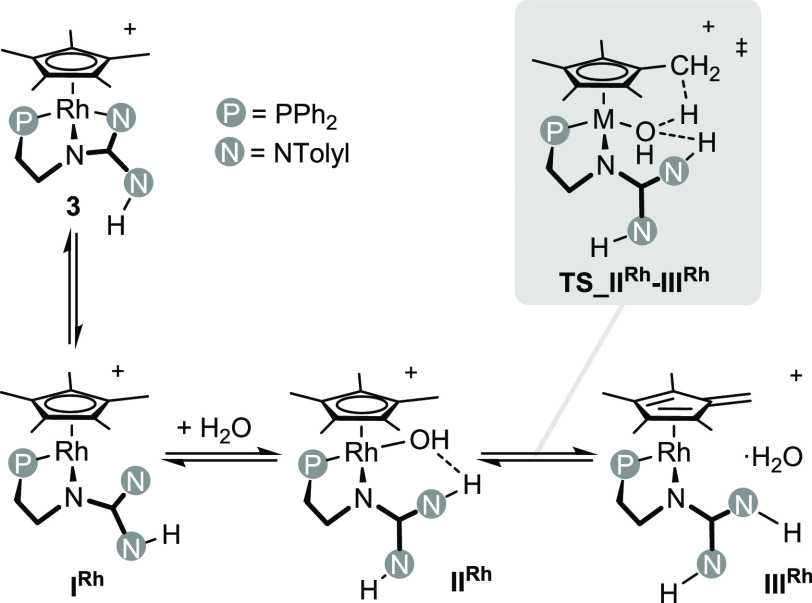
Reaction Sequence for the Hydrogen Exchange at **3**

Based on density functional theory (DFT) calculations, we previously reported^21^ that the H/D exchange relies on the activation of the water O–H bond at **I^Rh^** rendering the key hydroxo intermediate **II^Rh^** (Scheme 1). **II^Rh^** ultimately promotes the reversible hydrogen abstraction from Cp* (**TS_II^Rh^-III^Rh^**) and affords the rhodium(I)-fulvene complex **III^Rh^**, which should undergo a reversible H_2_O/D_2_O exchange, yielding the progressive hydrogen exchange/deuteration of the Cp* ligand (Scheme 1).

The Cp* ligand of the iridium complex **4** undergoes an H/D exchange process similar to that described for the rhodium analogue **3** but at a much slower rate. Indeed, at 293 K the ^1^H NMR spectrum of [D_8_]THF/D_2_O (78%/22%, v/v) solutions of the iridium complex **4** does not change over time. It is necessary to heat the reaction mixture at 343 K to observe the H/D exchange at an appreciable rate. After 4 days at this temperature, the Cp* ligand is deuterated at about 50%, on average. Apart from the isotopologues of **4** derived from the H/D exchange process, the formation of a new iridium complex, labeled as **8** (vide infra), was detected by NMR spectroscopy. The overlapping of the ^1^H NMR signals prevents the detailed study of the evolution of both the H/D exchange process and the reaction of formation of complex **8**. The complete characterization of **8** will be discussed in the next subsection.

**Figure 6:**
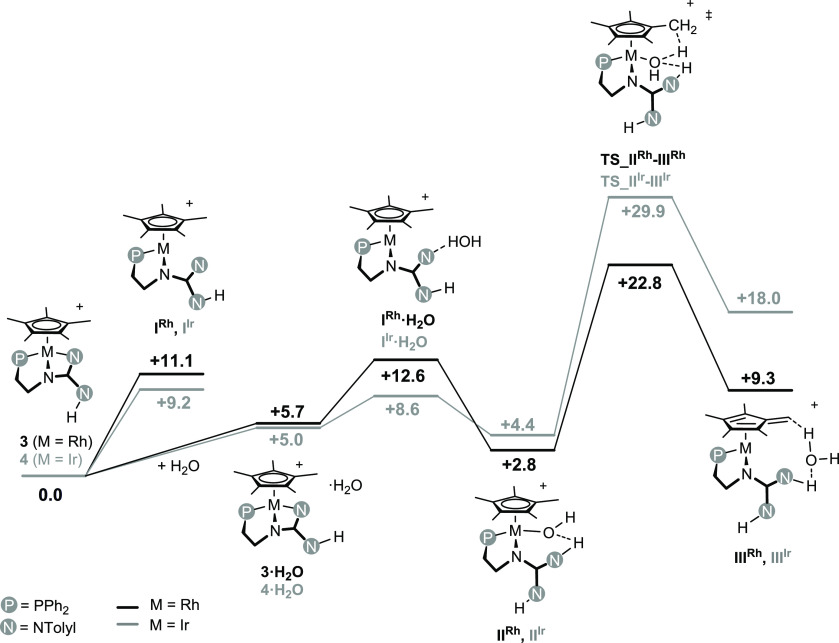
Gibbs free energy profile (kcal·mol^–1^) for the hydrogen exchange at **3** (black) and **4** (gray) in the presence of water [wB97XD/def2tzvp//wB97XD/def2svp, in THF (SMD model), 298 K, 1 atm].

For the sake of comparison, the Gibbs free energy profiles of the hydrogen exchange for both **3** and **4** were calculated at the level wB97XD/def2tzvp//wB97XD/def2svp, using the SMD model for the solvent, at 298 K. Figure 6 shows the calculated intermediates and transition states along with the relative Gibbs free energies.

**Figure 7:**
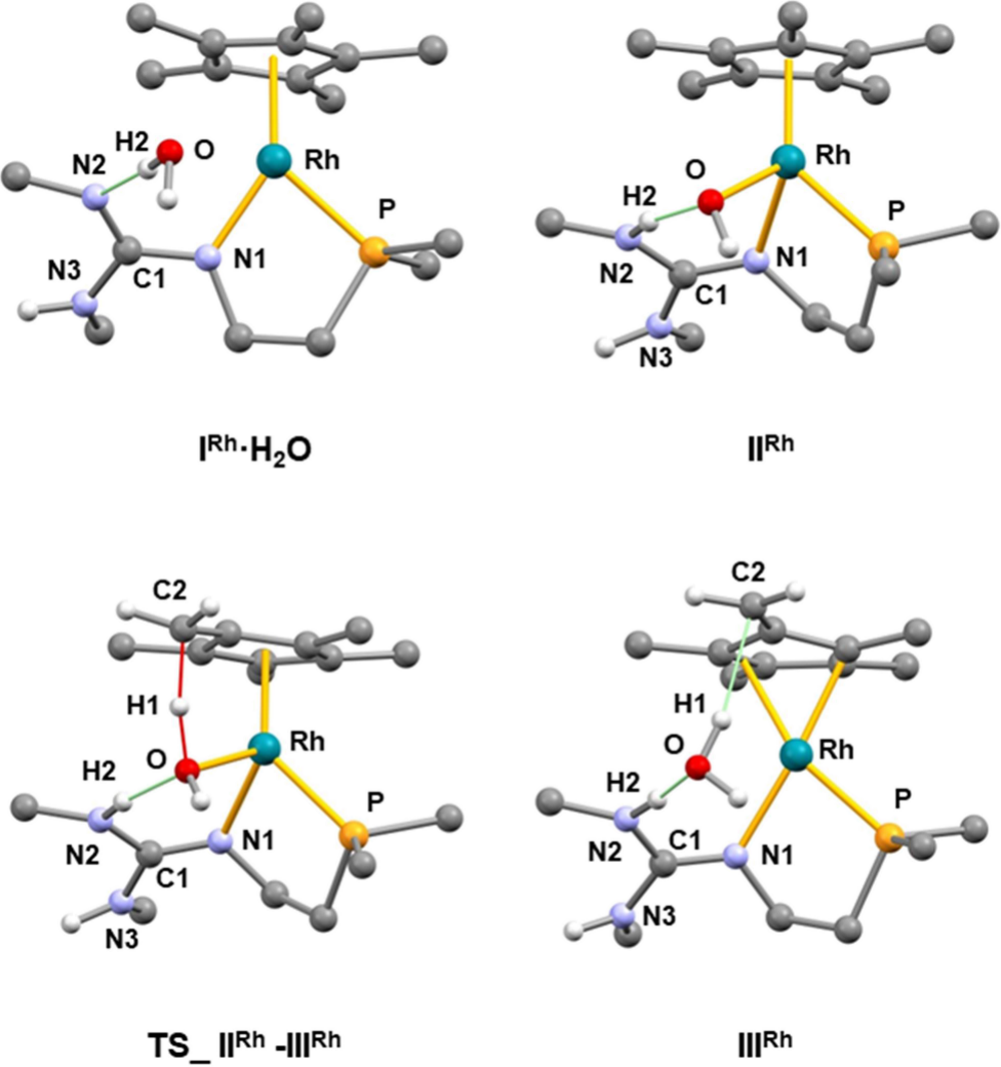
Calculated structures of **I^Rh^·H_2_O**, **II^Rh^**, **III^Rh^**, and **TS_II^Rh^-III^Rh^** with the numbering scheme adopted. The calculated structures of **I^Ir^·H_2_O**, **II^Ir^**, **III^Ir^**, and **TS_II^Ir^-III^Ir^** are similar and are not reported for the sake of brevity, the same numbering scheme being adopted. For clarity, most hydrogens are omitted, and only *ipso* carbon atoms of Tolyl and Phenyl groups are shown. Selected bond lengths/interatomic distances (Å) and angles (°) are: **I^Rh^·H_2_O**, N2–H2 1.836, O–H2 0.988, N2–O 2.820, N2–H2–O 173.4, Rh–O 3.651; **I^Ir^·H_2_O**, N2–H2 1.842, O–H2 0.986, N2–O 2.826, N2–H2–O 176.0, Ir–O 3.685; **II^Rh^**, Rh–O 2.078, O–H2 1.642, N2–H2 1.055, N2–O 2.655, N2–H2–O 159.4; **II^Ir^**, Ir–O 2.095, O–H2 1.647, N2–H2 1.051, N2–O 2.649, N2–H2–O 157.5; **TS_II^Rh^-III^Rh^**, Rh–O 2.219, O–H2 1.787, N2–H2 1.031, N2–O 2.787, N2–H2-O 162.2; C2–H1 1.429, O–H1 1.207, O–H1–C2 158.2; **TS_II^Ir^-III^Ir^**, Ir–O 2.255, O–H2 1.807, N2–H2 1.209, N2–O 2.798, N2–H2–O 160.4; C2–H1 1.485, O–H1 1.169, O–H1–C2 159.1; **III^Rh^**, Rh–O 3.562, O–H2 1.847, N2–H2 1.029, N2–O 2.866, N2–H2–O 170.1; C2–H1 2.267, O–H1 0.972, O–H1–C2 167.8; **III^Ir^**, Ir–O 3.616, O–H2 1.849, N2–H2 1.031, N2–O 2.865, N2–H2–O 167.7; C2–H1 2.196, O–H1 0.974, O–H1–C2 170.2.

DFT calculations indicate that for both **3** and **4** the hydroxo intermediates **II^Rh^** and **II^Ir^**, respectively, are obtained stepwise by reaction of water with **3** or **4**. Actually, the dissociation of the terminal M–N bond of **3** or **4** renders the true FLP complex, namely **I^Rh^** and **I^Ir^**, which interacts with one water molecule affording **I^Rh^·H_2_O** or **I^Ir^·H_2_O**. Neither **I^Rh^·H_2_O** nor **I^Ir^·H_2_O** contains a metal-oxygen bond, rather the incoming water molecule forms an N···H–O bond (Figure 7).^27^ Subsequent coordination of oxygen to the metal center and the concomitant H–OH bond rupture yield **II^Rh^** or **II^Ir^** in which a hydrogen bond still exists between the newly formed MOH and NH moieties (Figure 7). Once **II^Rh^** and **II^Ir^** form, hydrogen abstraction from its Cp* ligand gives the η^4^-tetramethylfulvene ligand in **III^Rh^** and **III^Ir^** and one weakly bonded water molecule (Figure 7). The hydrogen abstraction (**II^Rh^** → **III^Rh^**; **II^Ir^** → **III^Ir^**) entails the formal reduction of the metal center from the oxidation state +3 to +1. Accordingly, the metal centers of **III^Rh^** and **III^Ir^** feature a distorted square planar geometry in which two coordination sites are occupied by fulvene, whereas the phosphorus and nitrogen atoms from the phosphano-guanidine ligand complete the coordination sphere of the metal center (Figure 7). Notably, the metal-oxygen distance (**III^Rh^**, 3.562; **III^Ir^**, 3.616 Å) rules out the existence of a metal-oxygen bond. In addition, the water molecule is weakly bonded to the metal complex by means of the N2–H2···O hydrogen bond and an additional C2···H1–O short contact (Figure 7). Finally, the exchange of this weakly bonded water molecule with water (or D_2_O) solvent molecules should cause the progressive hydrogen exchange (deuteration) of the Cp* ligand. In view of the Gibbs free energy profiles given in Figure 6, it should be noted that intermediates **3**·H_2_O (or **4**·H_2_O) and **II^Rh^** (or **II^Ir^**), even if thermally accessible, are less stable than the starting complex **3** (or **4**), which nicely agrees with the fact that **3** (or **4**) are the only detected species in the course of the H/D exchange, and none of the intermediates has been observed by NMR spectroscopy.

As for the Gibbs free energy variation along the reaction sequence **3**/**4** + H_2_O ⇆ **III^Rh^**/**III^Ir^**, hydrogen exchange at **4** exhibits a significantly higher activation barrier (Δ*G*_act_ = +29.9 kcal mol^–1^) when compared with **3** (Δ*G*_act_ = +22.8 kcal mol^–1^), which perfectly fits in with the experimental conditions required for the H/D exchange of **3** and **4**, and with the observed degree of deuteration (vide supra).

**Scheme 2:**
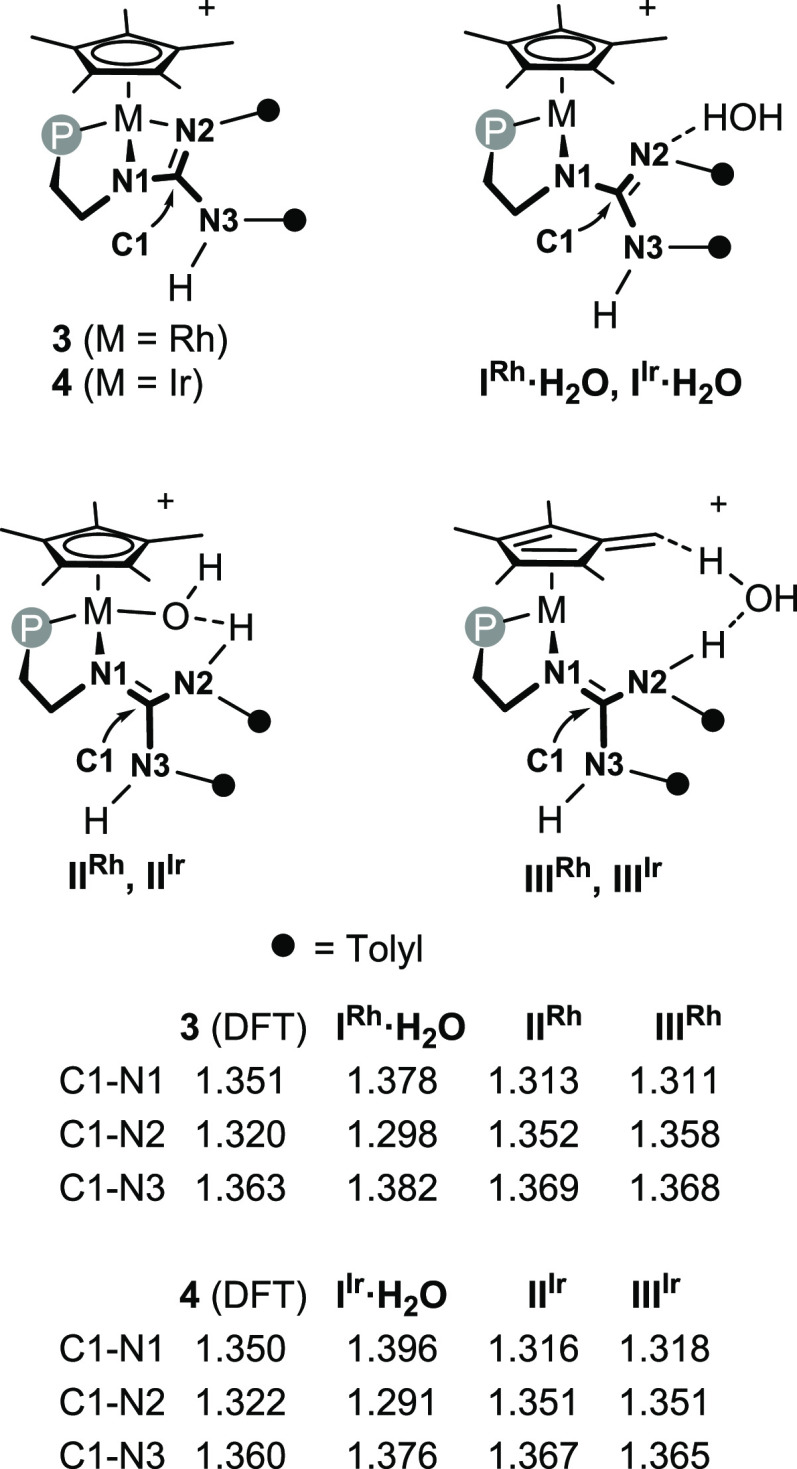
Calculated Carbon-Nitrogen Bond Lengths (Å) in **3**, **4**, **I^M^·H_2_O**, **II^M^**, and **III^M^** (M = Rh, Ir)

Finally, as far as the CN_3_ core is concerned, despite the fact that some degree of delocalization is expected to occur within the three carbon-nitrogen bonds, in the course of the hydrogen exchange a considerable electronic rearrangement takes place and it is reasonably beneficial to the accomplishment of the hydrogen exchange itself. Indeed, the analysis of the calculated carbon-nitrogen bond lengths for complexes **3**, **4**, **I^M^·H_2_O**, **II^M^**, **TS_II^M^-III^M^**, and **III^M^** (M = Rh, Ir, Scheme 2) points out that in the course of the hydrogen exchange the C1–N3 bond essentially holds its single bond character, whereas the C1–N1 and C1–N2 bonds switch from single to double and vice versa in the course of the sequence **I^M^·H_2_O** ⇆ **II^M^** ⇆ **III^M^**, the formal protonation of N2 triggering the switch from one electronic distribution to the other.

### Orthometalation Reactions

Heating THF/H_2_O (4/1, v/v) solutions of **3** or **4** at 383 K affords the orthometalated complexes **7** and **8**, respectively (eq 4).
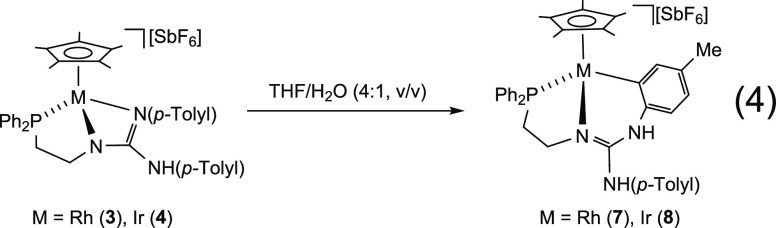
4

**Figure 8:**
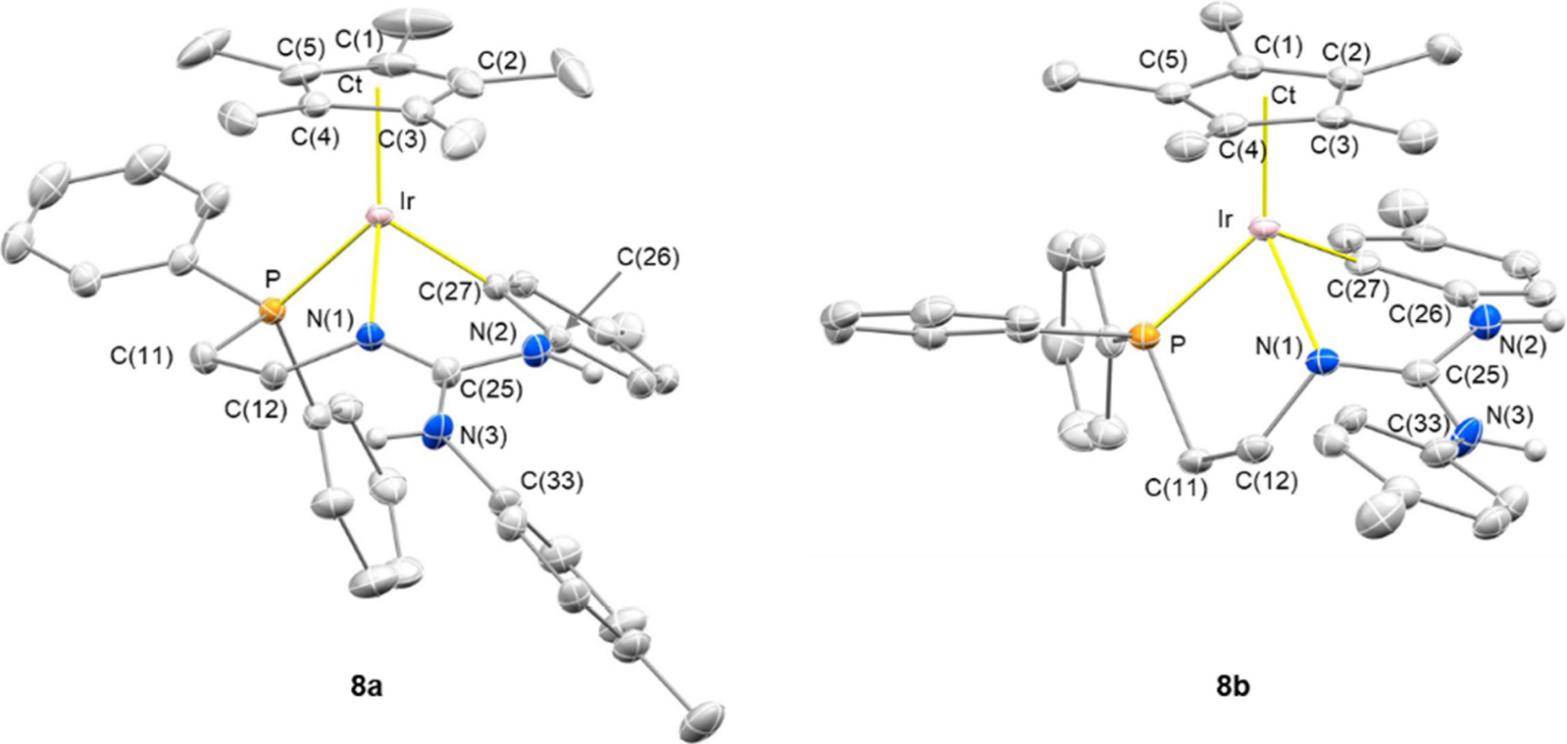
ORTEP plot of the cations of complexes **8a** and **8b**. Thermal ellipsoids are at 50% probability, and most hydrogen atoms have been omitted for clarity.

Single crystals of **7** and **8** were grown from THF/Et_2_O (**7**), CH_3_OH/Acetone/Et_2_O/*n*-pentane (**8a**, from here on) and CH_2_Cl_2_ (**8b**, from here on) solutions, **8a** and **8b** featuring different crystal structures. Figure 8 shows the ORTEP plot of the cations [Cp*Ir(κ^3^*C*,*N*,*P*-**H_2_L_-H_**)]^+^ in **8a** and **8b** [**H_2_L_-H_** = Ph_2_PCH_2_CH_2_NC(NH(*p*-Tolyl))(NH(4-C_6_H_3_Me))], and Table 4 contains selected bond lengths and angles. The rhodium compound **7** exhibits a crystal structure virtually superimposable to **8a**, so only **8a** will be discussed in detail and selected data of **7** are included in the Supporting Information.

**Table 4:** Selected Bond Lengths (Å) and Angles (°) of **8a** and **8b**

	**8a**	**8b**		**8a**	**8b**
Ir–P	2.2621(10)	2.2598(10)	P–Ir–Ct^a^	133.81(3)	135.48(3)
Ir–N(1)	2.081(3)	2.095(3)	N(1)–Ir–C(27)	84.25(14)	82.48(13)
Ir–C(27)	2.065(4)	2.069(4)	N(1)–Ir–Ct^a^	126.22(9)	128.99(9)
Ir–Ct^a^	1.8559(2)	1.8809(2)	C(27)–Ir–Ct^a^	125.66(10)	119.32(10)
N(1)–C(25)	1.312(5)	1.304(5)	Ir–N(1)–C(12)	118.0(2)	118.6(2)
N(2)–C(25)	1.355(5)	1.356(5)	Ir–N(1)–C(25)	122.2(3)	119.9(3)
N(3)–C(25)	1.363(5)	1.374(5)	C(12)–N(1)–C(25)	119.5(3)	120.4(3)
P–Ir–N(1)	77.05(9)	81.44(9)	Σ°N(1)^b^	359.7(5)	358.9(5)
P–Ir–C(27)	92.47(11)	93.21(11)	N(1)–C(25)–N(3)–C(33)	153.8(4)	–43.0(6)

a Ct represents the centroid of the η^5^-C_5_Me_5_ ligand.

b Σ°N(1) stands for the sum of bond angles around the N(1) atom.

Both **8a** and **8b** exhibit a three-legged-piano stool geometry with an η^5^ coordinated Cp* ligand. The metalated phosphano-guanidine ligand occupies three mutually *cis* positions at the metal center [**8a**, P–Ir–N(1) 77.05(9), P–Ir–C(27) 92.47(11), N(1)–Ir–C(27) 84.25(14)°; **8b**, P–Ir–N(1) 81.44(9), P–Ir–C(27) 93.21(11), N(1)–Ir–C(27) 82.48(13)°] rendering two fused metalacycles, namely, the five-membered ring Ir–P–C(11)–C(12)–N(1) and the six-membered ring Ir–C(27)–C(26)–N(2)–C(25)–N(1).

Interestingly, the metal center in both **8a** and **8b** is stereogenic. Nonetheless, as a consequence of the centrosymmetric space group *P*2_1_/*c* of **8a** and **8b**, both enantiomers, namely, *S*_Ir_-**8a**/**b** (shown in Figure 8) and *R*_Ir_-**8a**/**b**, are present in the crystal.^28^ When dealing with the differences between **8a** and **8b**, the arrangement of the exocyclic N(3)H(*p*-Tolyl) moiety with respect to the IrCp*(P)(N1)(C27) core is worth a mention. As a matter of fact, the dihedral angle N(1)–C(25)–N(3)–C(33) is 153.8(4)° in **8a** and–43.0(6)° in **8b** indicating that the N(3)–C(25) bond adopts a conformation close to *s-trans* in **8a** and close to *s-cis* in **8b** (Figure 8). As a final remark, when comparing **8a**/**8b** with **4**, reasonably as a consequence of the formation of the less strained six membered ring Ir–C(27)–C(26)–N(2)–C(25)–N(1) instead of the four membered ring Ir–N(1)–C(25)–N(2), the nitrogen atom N(1) exhibits a planar geometry both in **8a** [Σ°N(1) = 359.7(5)°] and **8b** [Σ°N(1) = 358.9(5)°]. On this ground, N(1) should adopt a sp^2^ hybridization in **8a** and **8b**. Accordingly, the N(1)–C(25) bond length is shorter [**8a**, 1.312(5); **8b**, 1.304(5) Å] than the N(2)–C(25) [**8a**, 1.355(5); **8b**, 1.356(5) Å] and N(3)–C(25) bond lengths [**8a**, 1.363(5); **8b**, 1.374(5) Å], suggesting that, despite some degree of delocalization over the CN_3_ core, the N(1)–C(25) bond should exhibit a higher double bond character when compared with N(2)–C(25) and N(3)–C(25).

No significant differences between the solution NMR spectra of both iridium rotamers **8a** and **8b** have been found in the 293–233 K temperature range indicating that, under these conditions, rotation around the N(3)–C(25) bond is free. Most probably, crystal packing accounts for the two dispositions encountered in the solid state.

The presence of two ^1^H peaks attributed to NH protons, at 8.41 and 8.36 ppm for **7**, and at 8.43 and 8.34 ppm for **8**, is indicative of the protonation of the N(*p*-Tolyl) group. The orthometalated carbon nucleus gives a doublet of doublets at 141.15 ppm [*J*(RhC) = 31.7 Hz, *J*(PC) = 13.8 Hz] for complex **7** and a doublet at 123.61 ppm (*J*(PC) = 9.1 Hz) for complex **8**. The ^31^P{^1^H} NMR spectrum consists of a doublet centered at 46.45 ppm [*J*(RhP) = 153.3 Hz] and one singlet at 19.01 ppm for **8**.

**Figure 9:**
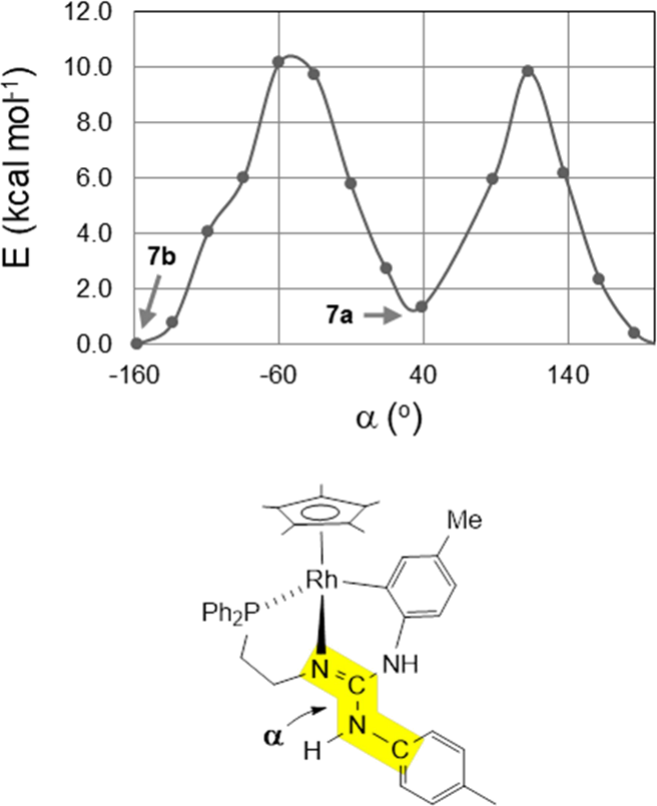
Energy profile (E vs α, wB97XD/def2svp, 298 K) for the rotation around the exocyclic C–N bond.

The energy profile E vs dihedral angle NCNC (α) for the rotation around the exocyclic C–N bond (Figure 9) was calculated for the rhodium complexes **7a**/**7b** showing that the barrier for the rotation is about 10 kcal mol^–1^. In addition, the Gibbs free energy differences between isomers **7a** and **7b** as well as between isomers **8a** and **8b** are small (G_**7a**_-G_**7b**_ = +1.4 kcal mol^–1^, G_**8a**_-G_**8b**_ = +0.1 kcal mol^–1^). On these grounds, both isomers for each metal should be present in solution at room temperature and the interconversions **7a** ⇆ **7b** and **8a** ⇆ **8b** should be fast at that temperature, which fits in with the observation of averaged NMR spectra for **7a** and **7b** as well as for **8a** and **8b**, and with the isolation of crystals of each isomer using different crystallization mixtures of solvents.

### Reaction of **3** or **4** with Alcohols

At 333 K, complexes **3** and **4** react in THF with methanol, primary alcohols, and 2-propanol cleanly giving the metal-hydrido complexes **5** and **6**, respectively (eq 5). The reaction involves the dehydrogenation of the alcohols at a relatively low^19,20^ temperature and without the assistance of an external base. ^1^H NMR signals assigned to methyl formate^19h^ [δ_H_ 8.07, brq; 3.76, brd], acetaldehyde [δ_H_ 9.67, q; 2.07, d (*J* = 2.8 Hz)], propionaldehyde [δ_H_ 9.56 t (*J* = 1.3 Hz)], benzaldehyde (δ_H_ 10.02, s), and acetone (δ_H_ 2.04, s) were detected after the reaction with methanol, ethanol, *n*-propanol, benzyl alcohol, and 2-propanol, respectively.
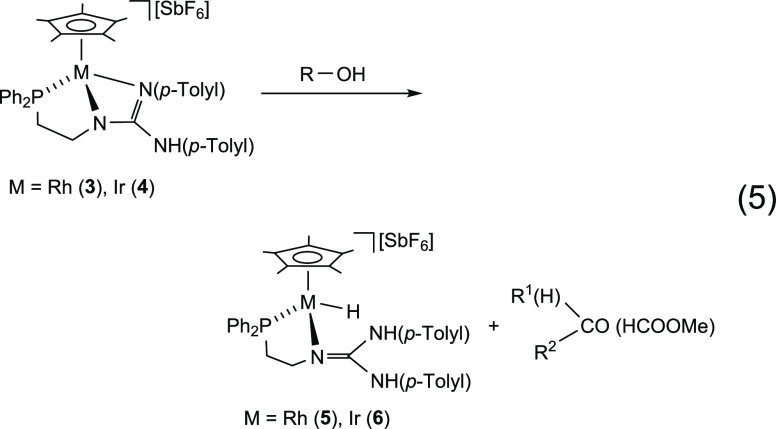
5

As the reactions with the iridium complex **4** were much slower than those with the rhodium compound **3**, kinetic studies were carried out only with **3**. Table 5 collects the values of the kinetic constants measured at 333 K (see the Supporting Information). The dehydrogenation rate is greater for methanol and primary alcohols (entries 1–4) than for the secondary alcohol 2-propanol (entry 5).^19h^ To obtain information about the mechanism, in independent experiments the reaction was carried out with CH_3_OD (entry 6) or CD_3_OH (entry 7). In the reaction with CD_3_OH, the metal-hydrido region of the ^1^H NMR spectrum of the resulting product was silent, but when CH_3_OD was used as a reagent, a Rh–H ^1^H resonance was observed in the product. Notably, values of 5.21 and 8.29 were obtained for the *k*_obs_(CH_3_OH)/*k*_obs_(CH_3_OD) and *k*_obs_(CH_3_OH)/*k*_obs_(CD_3_OH) ratios, respectively. A detailed kinetic study was not carried out with CD_3_OD because the reaction rate in this solvent is very low. Indeed, a conversion of only about 4% was measured after 60 h of reaction at 333 K.

**Table 5:** Kinetic Constant for the Reaction of Complex **3** with Alcohols at 333 K^a^

entry	alcohol	10^6^*k*_obs_/s^–1^
1	MeOH	8.12 ± 0.07
2	EtOH	11.6 ± 0.1
*3*	*n*PrOH	4.0 ± 0.2
4	BnOH	24.9 ± 0.5
5	*i*PrOH	1.12 ± 0.04
6	CH_3_OD	1.56 ± 0.03
7	CD_3_OH	1.0 ± 0.1

a See the SI for experimental details.

As it was observed in the reaction of **3** or **4** with deuterated water, mass and ^1^H NMR spectra of solutions of compound **3** in alcohols with deuterated hydroxo groups indicate that the progressive deuteration of the methyl groups of the Cp* ring occurs. Kinetic measurements establish that the deuteration process obeys a pseudo-first order rate law. Table 6 collects the values of the kinetic constants measured at 313 K, and for comparative purposes, *k*_obs_ obtained for D_2_O^21^ was also included. In general, the *k*_obs_ for the H/D exchange process is greater than that measured for the alcohol dehydrogenation. For example, for CH_3_OD, the *k*_obs_ for the Cp* deuteration is 6.39 ± 0.08 × 10^–4^ s^–1^ at 323 K (see the Supporting Information), and that for the dehydrogenation process is 1.56 ± 0.03 × 10^–6^ s^–1^, at 333 K (entry 6, Table 5), that is, the latter is about 400 times lower than the former despite being measured at a temperature 10 K higher. Based on the Eyring plot [ln(*k*_obs_/*T*) vs 1/*T*] Δ*G*^≠^_293_ of around 24 kcal·mol^–1^ was calculated in all cases (see the Supporting Information).

**Table 6:** Kinetic Constants for the H/D Exchange at 313 K^a^

entry	R–OD	10^5^*k*_obs_/s^–1^
1	D–OD	9.5 ± 0.2
2	CH_3_–OD	28.2 ± 0.6
3	CD_3_–OD	16.9 ± 0.2
4	Et–OD	7.73 ± 0.08
5	*i*Pr–OD	1.92 ± 0.04

a See the SI for experimental details.

The mechanism of the reactions of **3** and **4** with methanol was explored by DFT calculations in order to shed light on the deuteration of **3** and **4** in the presence of CH_3_OD as well as on the formation of **5** and **6**, respectively, as a result of the dehydrogenation of methanol. For both reactions, the energy profiles for **3** and **4** were elucidated by means of DFT computational methods at the level wB97XD/def2tzvp//wB97XD/def2svp using the SMD model for the solvent (THF).

**Figure 10:**
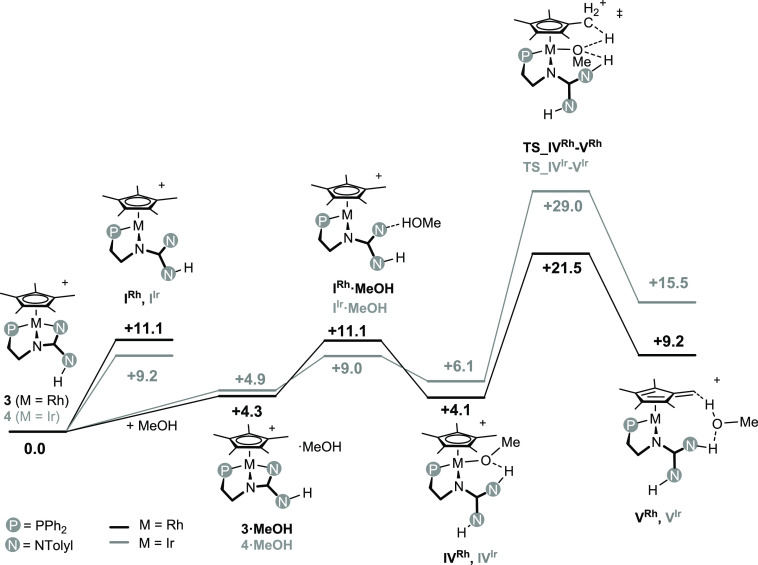
Gibbs free energy profile (kcal·mol^–1^) for the hydrogen exchange at **3** (black) and **4** (gray) in the presence of methanol [wB97XD/def2tzvp//wB97XD/def2svp, in THF (SMD model), 298 K, 1 atm].

As for the H/D exchange, the calculated reaction sequence is reminiscent of that previously discussed for the reaction of **3** or **4** with water (Figure 10). As a matter of fact, methanol reacts with **3** or **4** yielding **I^Rh^·MeOH** or **I^Ir^·MeOH**, respectively, in which a N···HO hydrogen bond brings together the dissociated form of **3** or **4**, namely, **I^Rh^** and **I^Ir^**, with a methanol molecule, no metal-oxygen bond being observed (vide infra). In the following, the rupture of the O–H bond affords the methoxo derivatives **IV^Rh^** and **IV^Ir^** featuring an intramolecular NH···O hydrogen bond between the newly formed NH group and the methoxo ligand. Similar to **II^Rh^** and **II^Ir^** (Figure 6), the Cp* ligand in **IV^Rh^** and **IV^Ir^** undergoes a hydrogen abstraction yielding the tetramethylfulvene metal(I) complexes **V^Rh^** and **V^Ir^**, respectively, in which the resulting methanol molecule is still involved in an NH···O hydrogen bond with the NH group. Also, a short OH···CH_2_^fulvene^ contact is observed between the fulvene ligand and the methanol molecule. Like for **III^Rh^** and **III^Ir^**, no metal-oxygen bond exists in **V^Rh^** and **V^Ir^**, and the exchange of the weakly bonded methanol molecule with methanol/methanol-*d_1_* solvent molecules triggers the hydrogen exchange/deuteration of the Cp* ligand. Similar to the reaction of **3** or **4** with water, the activation barrier for **3** + MeOH ⇆ **V^Rh^** (+21.5 kcal·mol^–1^) is significantly lower than that for **4** + MeOH ⇆ **V^Ir^** (+29.0 kcal mol^–1^), which nicely fits in with the experimental conditions and the outcome of the deuteration reaction with **3** or **4**.

**Figure 11:**
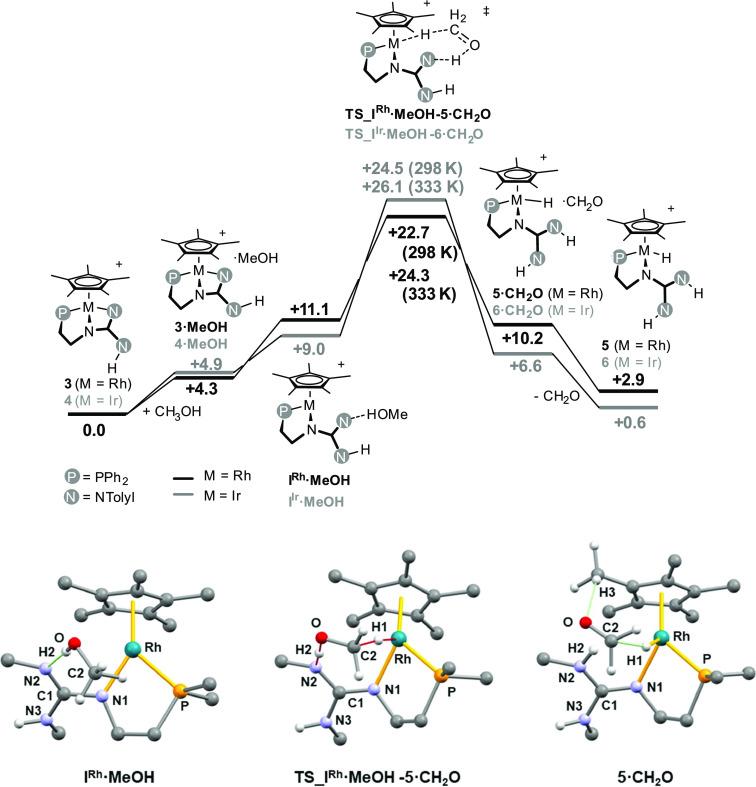
Gibbs free energy profile for the reaction **3** (or **4**) + MeOH → **5** (or **6**) + CH_2_O [wB97XD/def2tzvp//wB97XD/def2svp, in THF (SMD model), 298 K]. View of the **I^Rh^·MeOH**, **5·CH_2_O**, and **TS_I^Rh^·MeOH-5·CH_2_O** with the numbering scheme adopted. The calculated structures of **I^Ir^·MeOH**, **6·CH_2_O**, and **TS_I^Ir^·MeOH-6·CH_2_O** are similar and are not reported for the sake of brevity, the same numbering scheme being adopted. Selected bond lengths/interatomic distances (Å) and angles (°) are **I^Rh^·MeOH**, N2–H2 1.817, O–H2 0.988, N2–O 2.800, N2–H2–O 173.0, Rh–O 3.660, C2–O 1.397; **I^Ir^·MeOH**, N2–H2 1.821, O–H2 0.986, N2–O 2.802, N2–H2–O 172.8, Rh–O 3.673, C2–O 1.396; **TS_I^Rh^·MeOH-5·CH_2_O**, Rh–H1 1.832, H1–C1 1.198, C2–O 1.322, O–H2 1.279, N2–H2 1.206; **TS_I^Ir^·MeOH-6·CH_2_O**, Ir–H1 1.863, H1–C1 1.211, C2–O 1.319, O–H2 1.268, N2–H2 1.216; **5·CH_2_O**, H3–O 2.447, H1–C2 2.433, C2–O 1.206; **6·CH_2_O**, H3–O 2.436, H1–C2 2.413, C2–O 1.205.

As for the dehydrogenation of methanol rendering **5** or **6**, DFT calculations suggest that **I^Rh^·MeOH** and **I^Ir^·MeOH** are again key intermediates (Figure 11). As a matter of fact, they convert into **5·CH_2_O** or **6·CH_2_O**, respectively, via the concerted transition state **TS_I^Rh^·MeOH-5·CH_2_O** or **TS_I^Ir^·MeOH-6·CH_2_O**. Notably, the elimination of CH_2_O results from the simultaneous migration of one CH hydrogen atom to the metal center and of the OH hydrogen atom to a nitrogen atom of the guanidine moiety (*cf*. **TS_I^Rh^·MeOH-5·CH_2_O**, Figure 11). Accordingly, the carbon–oxygen bond shortens from 1.396 Å (av.) to 1.206 Å (av.) on going from **I^Rh^·MeOH** and **I^Ir^·MeOH** to **5·CH_2_O** and **6·CH_2_O**, respectively (Figure 11).

In this connection, previously reported studies already indicated that mono-^29^ and dinuclear^30^ iridium complexes as well as ruthenium derivatives^31^ are able to perform the acceptorless dehydrogenation of methanol via a concerted transition state taking advantage of the bifunctional character of the metal–ligand platform.

The calculated barriers for the rhodium complex **3** (+22.7 kcal·mol^–1^ at 298 K, +24.3 kcal·mol^–1^ at 333 K) and for the iridium complex **4** (+24.5 kcal·mol^–1^ at 298 K, +26.1 kcal·mol^–1^ at 333 K) underpin the experimental conditions. It is worth mentioning that according to the Gibbs free energy profile given in Figure 11 the above mentioned overall reactions are slightly endergonic (+2.9 kcal mol^–1^, M = Rh; +0.6 kcal mol^–1^, M = Ir). Nonetheless, in this regard, as mentioned before, methyl formate was detected as a product in the reaction of **3** or **4** with methanol, and its formation from formaldehyde was calculated to be exergonic (CH_2_O → 1/2 HCOOCH_3_, Δ*G*_r_ = −9.7 kcal mol^–1^) which compensate the above mentioned positive Δ*G*_r_. On the other hand, when ethanol, 2-propanol, and benzyl alcohol were used, the overall dehydrogenation reaction CHR_2_OH + **3** (or **4**) → CR_2_O + **5** (or **6**) was calculated to be exergonic (Δ*G*_r_ = from −10.8 to −4.5 kcal·mol^–1^) in agreement with the observation of acetaldehyde, acetone, and benzaldehyde, respectively, in the reaction mixture.

## Conclusions

Compounds **3** and **4** behave like masked TMFLPs. The *fac* κ^3^*N*,*N′*,*P* coordination of the phosphano-guanidine ligand forces the central nitrogen atom to adopt an sp^3^ hybridization thus generating a strong strain within the M–N–C–N four-membered metalacycle. This structural stress makes the “unmasked” TMFLP thermally accessible (eq 6). The metal (acidic center) and the iminic nitrogen (basic center) synergistically cooperate in the reversible activation of molecular hydrogen as well as in the activation of the O–H bond of water and alcohols. The resulting nucleophilic M–OH and M–OR fragments are able to reversibly dehydrogenate the Cp* methyl groups giving rise to sequential and complete H/D exchange of the Cp* protons when deuterated D–OD or R–OD solvents were employed. On the other hand, the “unmasked” TMFLP is also able to dehydrogenate alcohols affording metal hydrido derivatives via a concerted transition state involving simultaneously the acidic and the basic sites.
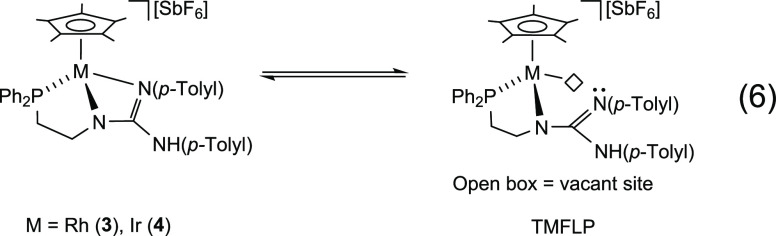
6

In this respect, the greater reactivity of the aldehyde or ketone products of the alcohol dehydrogenation versus the starting alcohol together with the reversibility of the hydrogenation reaction of complexes **3** and **4** paves the way to the potential application of these complexes to catalyzed reactions of alcohols using borrowing hydrogen methodology. On the other hand, judicious design of tridentate ligands capable of a *fac* κ^3^*N*,*N′*,*P* coordination as well as incorporation of d^6^ ions of precious and nonprecious metals would dramatically expand the applicability of the derived TMFLP species both in small-molecule activation chemistry and in the development of new catalytic processes.

Further work in this area is in progress and will be reported in due course.

## Supporting Information Available

The synthesis and characterization of the complexes **1**–**8**; ^1^H, ^31^P{^1^H}, and ^13^C{^1^H} spectra for the complexes **2**, **4**, **6**, **7**, and **8**; CD of complexes **1** and **2**; dehydrogenation reaction of complexes **5** and **6**; kinetic studies for the H/D exchange at Cp* of complex **3**; kinetic studies for acceptorless alcohol dehydrogenation; crystal structure determination of **1**, **2, 4**, **7**, **8a**, and **8b**; DFT calculations, and energies and coordinates of calculated structures (PDF)
