# Supplementary material for: Activation of H–H, HO–H, C(sp2)–H, C(sp3)–H, and RO–H Bonds by Transition-Metal Frustrated Lewis Pairs Based on M/N (M = Rh, Ir) Couples
Source: Inorg Chem. 2022 Aug 10;61(33):13149–64. doi: 10.1021/acs.inorgchem.2c01902 (PMC9406284; doi:10.1021/acs.inorgchem.2c01902)
Supplement: Supplementary file 1 — ic2c01902_si_001.pdf [file ic2c01902_si_001.pdf]

Supporting Information

Activation of H−H, HO–H, C(sp^2)−H, C(sp^3)−H and RO−H Bonds by Transition Metal Frustrated Lewis Pairs Based on M/N (M = Rh, Ir) Couples

María Carmona,[a] Roberto Pérez,[a] Joaquina Ferrer,*,[a] Ricardo Rodríguez,*[a] Vincenzo Passarelli,*,[a] Fernando J. Lahoz,[a] Pilar García-Orduña,[a] and Daniel Carmona*[a]

[a] Instituto de Síntesis Química y Catálisis Homogénea (ISQCH), CSIC - Universidad de Zaragoza, Departamento de Química Inorgánica, Pedro Cerbuna 12, 50009 Zaragoza, Spain, E-mail: dcarmona@unizar.es (D. C.), jfecer@unizar.es (J. F.) riromar@unizar.es, (R. R.), passarel@unizar.es (V. P.)

Table of Contents

1. General information **S2**

2. Synthesis and characterization of the complexes 1-8 **S2**

3. ^{1}H, ^{31}P{^{1}H} and ^{13}C{^{1}H} spectra for the complexes 2, 4, 6, 7, and 8 **S11**

4. CD of complexes 1 and 2 **S19**

5. Dehydrogenation reaction of complexes 5 and 6 **S22**

6. Kinetic studies for the H/D exchange at Cp* of complex 3 **S23**

7. Kinetic studies for acceptorless alcohol dehydrogenation **S31**

8. Crystal structure determination of 1, 2, 4, 7, 8a and 8b **S35**

9. DFT calculations **S41**

10. Energies and coordinates of calculated structures **S42**

11. References **S99**

1. General information

All preparations have been carried out under argon, unless otherwise stated. All solvents were treated in a PS-400-6 Innovative Technologies Solvent Purification System (SPS). Infrared spectra were recorded on Perkin-Elmer Spectrum-100 (ATR mode) FT-IR spectrometer. Carbon, hydrogen and nitrogen analyses were performed using a Perkin-Elmer 240 B microanalyzer. ^{1}H, ^{13}C and ^{31}P spectra were recorded on a Varian UNITY 300, a Bruker AV-300 (300.13 MHz), a Bruker AV-400 (400.16 MHz) or a Bruker AV-500 (500.13 MHz) spectrometers. Chemical shifts are expressed in ppm upfield from $SiMe_4$ or 85% H_3PO_4 (^{31}P). *J* values are given in Hz. Using standard procedures, the correlation spectra of COSY, NOESY, HSQC, HMQC, and HMBC ^{1}H-X (X = ^{1}H, ^{13}C, ^{31}P) were obtained. Mass spectra were obtained with a Micro Tof-Q Bruker Daltonics spectrometer. CD spectra were determined in a 1 cm path length cell by using a Jasco-710 apparatus.

2. Synthesis and characterization of the complexes 1-8

Preparation and characterization of the complexes [Cp*MCl(κ^{2}*N,P*−H_2L)][SbF_6] (M = Rh, 1; Ir, 2)

Under argon atmosphere, to a suspension of the corresponding dimer [$(Cp^*MCl)_2(\mu\text{-}Cl)_2$] (834.2 mg, (Rh); 1075.56 mg (Ir); 1.35 mmol) in MeOH (15 mL), a solution of **H_2L** (1.22 g, 2.70 mmol) in MeOH (5 mL) and $NaSbF_6$ (698.4 mg, 2.70 mmol) were added. After 12 h of stirring, the resulting solution was vacuum-evaporated until dryness and the residue was extracted in CH_2Cl_2 (3 × 5 mL). The resulting solution was vacuum-concentrated until ca. 3 mL. The addition of *n*-hexane afforded an orange (Rh) or yellow (Ir) solid which was filtered off, washed with *n*-hexane (3 × 5 mL) and vacuum-dried. Yield: 2.41 g, 93 % (Rh); 1.11 g, 78 % (Ir). Crystals suitable for X-ray diffraction analysis were obtained by crystallization from CH_2Cl_2/Et_2O/*n*-pentane solutions of **1** or **2**.

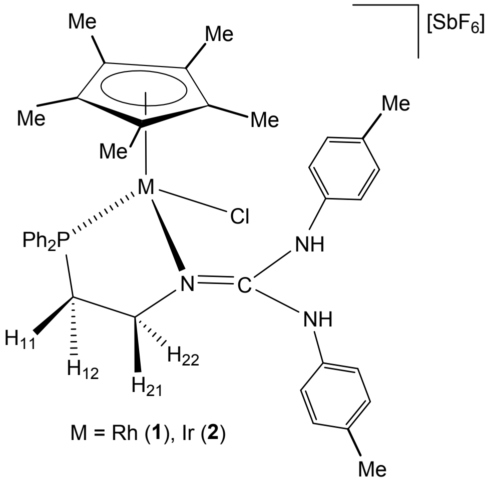

M = Rh (**1**), Ir (**2**)

Compound **1**

Anal. calcd for $C_{39}H_{45}ClF_6N_3PRhSb$, %: C, 48.75; H, 4.72; N, 4.37. Found, %: C, 48.36; H, 4.52; N, 4.41. HRMS (μ-TOF): $C_{39}H_{45}ClF_6N_3PRhSb$ $[M\text{-}SbF_6]^+$: calcd 724.2089, found 724.2092. IR (solid, cm^{-1}): ν(NH) 3374 (w), 3250-3200 (br); ν(N=C) 1609 (m); $\nu(SbF_6)$ 657 (s). *^1H NMR (400.16 MHz, $CDCl_3$, RT, ppm):* δ = 9.24 (s, 1H, $NH_{cis\text{ to Rh}}$), 7.64-7.43 (m, 8H, PPh_2), 7.34 (s, 1H, $NH_{trans\text{ to Rh}}$), 7.28 (m, 2H, PPh_2), 6.88 (d, *J* = 8.4 Hz, 2H, CH(*p*-Tolyl)), 6.84 (d, *J* = 8.4 Hz, 2H, CH(*p*-Tolyl)), 6.80 (d, *J* = 8.4 Hz, 2H, CH(*p*-Tolyl)), 6.74 (d, *J* = 8.4 Hz, 2H, CH(*p*-Tolyl)), 4.34 (ddd, *J* = 44.5, 13.0, 6.7 Hz, 1H, H_{22}), 3.88 (tt, *J* = 14.0, 4.1 Hz, 1H, H_{21}), 2.83 (td, *J* = 14.1, 2.2 Hz, 1H, H_{11}), 2.36 (m, 1H, H_{12}), 2.16 (s, 3H, Me(*p*-Tolyl)), 2.13 (s, 3H, Me(*p*-Tolyl)), 1.47 (d, *J* = 3.6 Hz, 15 H, C_5Me_5). *$^{13}C\{^1H\}$ NMR (100.62 MHz, $CDCl_3$, RT, ppm):* δ = 153.85 (C=N), 135.93 (C(*p*-Tolyl)), 135.55 (C(*p*-Tolyl)), 133.95 (d, *J* = 9.4 Hz, 2C, PPh_2), 132.80 (C(*p*-Tolyl)), 132.66 (C(*p*-Tolyl)), 132.63 (dd, *J* = 42.4, 1.1 Hz, C(PPh_2)), 132.13 (d, *J* = 9.0 Hz, 2C, PPh_2), 131.90 (d, *J* = 2.4 Hz, PPh_2), 131.79 (d, *J* = 2.8 Hz, PPh_2), 129.75 (2C, CH(*p*-Tolyl)), 129.64 (2C, CH(*p*-Tolyl)), 129.32 (d, *J* = 10.2 Hz, 2C, PPh_2), 129.03 (d, *J* = 10.8 Hz, 2C, PPh_2), 128.28 (d, *J* = 54.3 Hz, C(PPh_2)), 119.04 (2C, CH(*p*-Tolyl)), 118.80 (2C, CH(*p*-Tolyl)), 100.90 (dd, *J* = 6.7, 2.5 Hz, 5C, C_5Me$_5$), 54.32 (d, *J* = 2.2 Hz, NCH_2), 34.62 (d, *J* = 29.6 Hz, PCH_2), 20.79 (Me(*p*-Tolyl)), 20.75 (Me(*p*-Tolyl)), 9.29 (5C, C_5Me_5). *$^{31}P\{^1H\}$ NMR (161.98 MHz, $CDCl_3$, RT, ppm):* δ = 51.37 (d, *J* = 142.4 Hz).

CD spectra. (*S*)-**1**, CH_2Cl_2, 4.39 × 10^{-4} M, RT, λ(nm), (Δε): (307 (-4.27), 353 (+5.19), 396 (-2.96), 461 (+3.16). (*R*)-**1**, CH_2Cl_2, 5.58 × 10^{-4} M, RT, λ(nm), (Δε): (307 (+4.91), 353 (-6.17), 396 (+3.54), 461 (-3.63).

Compound **2**

Anal. calcd for $C_{39}H_{45}ClF_6IrN_3PSb$: C, 44.60; H, 4.32; N, 4.00. Found: C, 43.95; H, 4.00; N, 4.07. HRMS (*μ*-TOF): $C_{39}H_{45}ClF_6IrN_3PSb$ $[M-SbF_6]^+$: calcd 814.2657, found 814.2654. IR (cm^{-1}): *ν*(NH) 3370-3050 (br); *ν*(N=C) 1609 (m); *ν*(SbF_6) 657 (s). *^1H NMR (400.16 MHz, $CDCl_3$, RT, ppm):* δ = 8.93 (s, 1H, $NH_{cis\ \mathrm{to\ Ir}}$), 7.57-7.33 (m, 8H, PPh_2), 7.16 (m, H, $NH_{trans\ \mathrm{to\ Ir}}$, PPh_2), 6.80 (d, *J* = 8.0 Hz, 2H, CH(*p*-Tolyl)), 6.76 (d, *J* = 8.1 Hz, 2H, CH(*p*-Tolyl)), 6.69 (d, *J* = 8.6 Hz, 2H, CH(*p*-Tolyl)), 6.66 (d, *J* = 7.8 Hz, 2H, CH(*p*-Tolyl)), 4.29 (ddd, *J* = 40.8, 12.9, 6.8 Hz, 1H, H_{22}), 3.78 (tt, *J* = 13.9, 4.6 Hz, 1H, H_{21}), 2.85 (td, *J* = 13.2, 2.9 Hz, 1H, H_{11}), 2.27 (m, 1H, H_{12}), 2.09 (s, 3H, Me(*p*-Tolyl)), 2.06 (s, 3H, Me(*p*-Tolyl)), 1.44 (d, *J* = 2.1 Hz, 15 H, C_5Me_5). *$^{13}C\{^1H\}$ NMR (100.62 MHz, $CDCl_3$, RT, ppm):* δ = 154.07 (C=N), 135.79 (C(*p*-Tolyl)), 135.18 (C(*p*-Tolyl)), 133.97 (d, *J* = 10.0 Hz, 2C, PPh_2), 132.89 (C(*p*-Tolyl)), 132.82 (C(*p*-Tolyl)), 132.39 (d, *J* = 53.7 Hz, C(PPh_2)), 132.05 (d, *J* = 9.3 Hz, 2C, PPh_2), 131.89 (d, *J* = 2.3 Hz, PPh_2), 131.83 (d, *J* = 2.7 Hz, PPh_2), 129.69 (2C, CH(*p*-Tolyl)), 129.59 (2C, CH(*p*-Tolyl)), 129.16 (d, *J* = 11.2 Hz, 2C, PPh_2), 129.03 (d, *J* = 11.7 Hz, 2C, PPh_2), 126.66 (d, *J* = 63.2 Hz, C(PPh_2)), 119.16 (2C, CH(*p*-Tolyl)), 118.86 (2C, CH(*p*-Tolyl)), 94.43 (d, *J* = 2.7 Hz, 5C, C_5Me$_5$), 57.03 (NCH_2), 34.95 (d, *J* = 35.5 Hz, PCH_2), 20.79 (Me(*p*-Tolyl)), 20.75 (Me(*p*-Tolyl)), 8.87 (5C, C_5Me_5). *$^{31}P\{^1H\}$ NMR (161.98 MHz, $CDCl_3$, RT, ppm):* δ = 26.52 (s).

CD spectra. (*R*)-**2**, CH_2Cl_2, 3.81 × 10^{-4} M, RT, λ(nm), (Δε): 312 (-3.99), 334 (+1.56), 370 (-2.56), 385 (-2.99), 408 (-3.66). (*S*)-**2**, CH_2Cl_2, 5.95 × 10^{-4} M, RT, λ(nm), (Δε): 312 (+6.10), 334 (-2.31), 370 (+4.23), 385 (+4.82), 408 (+5.75).

Preparation and characterization of the complexes [Cp*M(κ^3*N,N',P*-HL)][SbF_6] (M = Rh, 3; Ir, 4).

At 278 K, to a solution of NaOH (25.0 mg, 0.62 mmol) in H_2O (5 mL) a solution of the corresponding complex [Cp*MCl(κ^2*N,P*-**H_2L**)][SbF_6] (400.0 mg, (Rh); 441.1 mg, (Ir); 0.42 mmol) in 1:1 (v/v) THF/toluene (20 mL) was added. The resulting biphasic suspension was stirred for 1.5 hours. Then, after the addition of 35 mL of H_2O, the organic phase was extracted with a 1:1 (v/v) solution of THF/toluene (3 × 10 mL) and evaporated to dryness. The obtained dark red (Rh) or yellow (Ir) residue was washed with Et_2O (3 × 5 mL) and *n*-pentane (3 × 5 mL) and vacuum-dried. Recrystallization in THF/Et_2O/*n*-pentane afforded complexes **3** and **4**. Yield: 280.9 mg, 73 % (Rh); 349.1 mg, 82 % (Ir). Crystals suitable for X-ray diffraction analysis were obtained by crystallization from THF/Et_2O/*n*-pentane solutions.

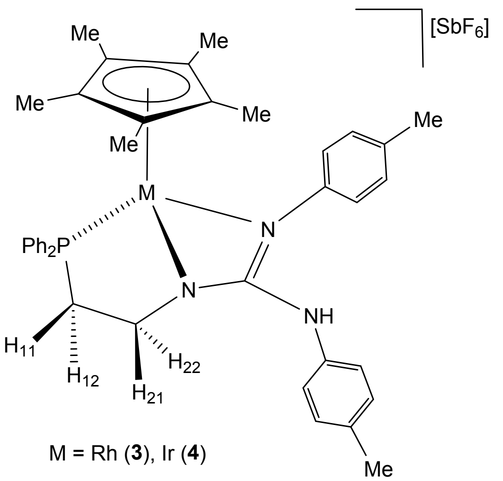

Compound **3**. For analytical and spectroscopic data of this complex see ref. S1.

Compound **4**.

Anal. calcd for $C_{39}H_{44}F_6IrN_3PSb$: C, 46.21; H, 4.37; N, 4.15. Found: C, 46.20; H, 4.57; N, 4.01. HRMS (μ-TOF): $C_{39}H_{44}F_6IrN_3PSb$ [M-SbF_6]$^+$: calcd 778.2899, found 778.2861. IR (cm^{-1}): ν(NH) 3362 (w); ν(N=C) 1595 (m); ν(SbF_6) 653 (s). *^{1}H NMR (300.13 MHz, THF-d_8, RT, ppm):* δ = 8.00 (brs, 1H, NH),

7.86 (m, 2H, PPh_2), 7.55 (m, 3H, PPh_2), 7.38 (m, 3H, PPh_2), 7.19 (m, 2H, PPh_2), 6.85 (d, J = 8.5 Hz, 4H, CH(*p*-Tolyl)), 6.75 (brd, J = 8.5 Hz, 2H, CH(*p*-Tolyl)), 6.60 (d, J = 8.5 Hz, 2H, CH(*p*-Tolyl)), 3.23 (ddd, J = 34.9, 13.6, 6.3 Hz, 1H, H_{22}), 3.01 (tt, J = 12.8, 5.1 Hz, 1H, H_{21}), 2.68 - 2.40 (m, 2H, H_{11}, H_{12}), 2.11 (s, 3H, Me(*p*-Tolyl)), 2.08 (s, 3H, Me(*p*-Tolyl)), 1.41 (d, J = 2.0 Hz, 15H, C_5Me_5). *$^{13}C\{^1H\}$ NMR (125.77 MHz, THF-d_8, RT, ppm):* δ = 169.65 (C=N), 139.98 (C(*p*-Tolyl)), 138.12 (C(*p*-Tolyl)), 135.50 (d, J = 11.6 Hz, 2C, PPh_2), 134.65 (d, J = 53.6 Hz, C(PPh_2)), 133.77 (C(*p*-Tolyl)), 132.88 (d, J = 1.7 Hz, PPh_2), 132.67 (C(*p*-Tolyl)), 132.17 (d, J = 9.5 Hz, 2C, PPh_2), 131.89 (d, J = 2.0 Hz, PPh_2), 131.59 (d, J = 52.6 Hz, C(PPh_2)), 130.45 (2C, CH(*p*-Tolyl)), 130.18 (d, J = 10.1 Hz, 2C, PPh_2), 130.17 (2C, CH(*p*-Tolyl)), 129.69 (d, J = 10.6 Hz, 2C, PPh_2), 123.94 (2C, CH(*p*-Tolyl)), 119.71 (2C, CH(*p*-Tolyl)), 93.52 (d, J = 2.9 Hz, 5C, C_5Me_5), 54.55 (NCH_2), 35.00 (d, J = 38.0 Hz, PCH_2), 20.96 (Me(*p*-Tolyl)), 20.91 (Me(*p*-Tolyl)), 9.11 (5C, C_5Me_5). *$^{31}P\{^1H\}$ NMR (121.42 MHz, THF-d_8, RT, ppm):* δ = 27.75 (s).

Preparation and characterization of the complexes [Cp*MH(κ^2*N,P*−H_2L)][SbF_6] (M = Rh, 5; Ir, 6)

To a high-pressure NMR tube containing a solution of the corresponding complex [Cp*M(κ^3*N,N',P*-**HL**)][SbF_6] (27.4 mg, (Rh); 30.7 mg, (Ir); 0.030 mmol) in THF-d_8 (0.45 mL), H_2 (4 bar) was added and the resulting solution was monitorized by NMR. After 4 h at room temperature, the conversion to the rhodium complex **5** was complete. Conversion to the iridium complex **6** was complete after 24 h at 373 K. Both complexes were characterized in situ (under H_2 atmosphere) by NMR. Crude complexes **5** and **6** were obtained by evaporation of the solvent. Yield: 19.1 mg, 69 % (**5**); 22.1 mg, 72 % (**6**). Crystals suitable for X-ray diffraction analysis were obtained from MeOH/diethyl ether solutions of **5**.

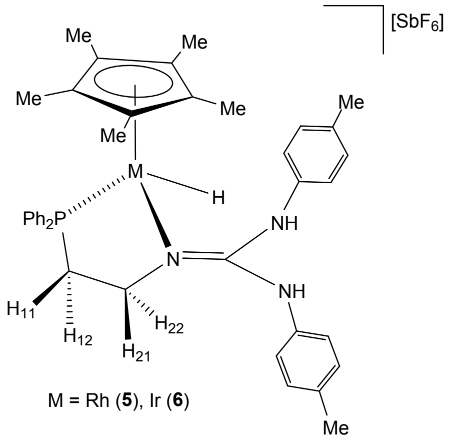

Compound **5**. For analytical and spectroscopic data of this complex see ref. S1.

Compound **6**.

Anal. calcd for $C_{39}H_{46}F_6IrN_3PSb$: C, 46.12; H, 4.56; N, 4.14. Found: C, 46.52; H, 5.00; N, 4.14. HRMS (μ-TOF): $C_{39}H_{46}IrN_3P$ $[M-SbF_6]^+$: calcd 780.3055, found 780.3031. IR (cm^{-1}): ν(NH) 3374, 3288 (w); ν(IrH) 2921 (m); ν(N=C) 1605 (m); ν(SbF_6) 654 (s). *^1H NMR (400.16 MHz, THF-d_8, RT, ppm): δ =* 7.99 (br, 1H, $NH_{cis\ \mathrm{to\ Ir}}$), 7.60 (m, 2H, PPh_2), 7.49 (s, 1H, $NH_{trans\ \mathrm{to\ Ir}}$), 7.38 (m, 6H, PPh_2), 7.12 (m, 2H, PPh_2), 6.96 (d, J = 8.1 Hz, 2H, CH(*p*-Tolyl)), 6.92 (d, J = 8.4 Hz, 2H, CH(*p*-Tolyl)), 6.85 (d, J = 8.5 Hz, 2H, CH(*p*-Tolyl)), 6.72 (d, J = 8.5 Hz, 2H, CH(*p*-Tolyl)), 3.98 (ddd, J = 40.6, 12.5, 6.5 Hz, 1H, H_{22}), 3.15 (tt, J = 13.3, 4.3 Hz, 1H, H_{21}), 2.82 (td, J = 12.9, 3.8 Hz, 1H, H_{11}), 2.11 (s, 3H, Me(*p*-Tolyl)), 2.10 (s, 3H, Me(*p*-Tolyl)), 1.86 (m, 1H, H_{12}), 1.61 (d, J = 1.8 Hz, 15H, C_5Me_5), -14.16 (d, J = 32.3 Hz, 1H, IrH). *$^{13}C\{^1H\}$ NMR (100.62 MHz, THF-d_8, RT, ppm): δ =* 155.12 (d, J = 1.1 Hz, C=N), 138.70 (C(*p*-Tolyl)), 137.66 (C(*p*-Tolyl)), 135.47 (d, J = 38.9 Hz, C(PPh_2)), 135.42 (d, J = 11.1 Hz, 2C, PPh_2), 134.89 (d, J = 55.7 Hz, C(PPh_2)), 134.55 (C(*p*-Tolyl)), 133.17 (C(*p*-Tolyl)), 132.42 (d, J = 2.7 Hz, P PPh_2), 132.28 (d, J = 9.6 Hz, 2C, PPh_2), 131.66 (d, J = 2.3 Hz, PPh_2), 130.96 (2C, CH(*p*-Tolyl)), 130.92 (2C, CH(*p*-Tolyl)), 129.68 (d, J = 10.5 Hz, 2C, PPh_2), 129.64 (d, J = 11.1 Hz, 2C, PPh_2), 121.05 (2C, CH(*p*-Tolyl)), 119.33 (2C, CH(*p*-Tolyl)), 94.93 (d, J = 2.8 Hz, 5C, *C_5*Me_5), 60.66 (d, J = 1.6 Hz,

NCH$_2$), 37.98 (d, J = 36.1 Hz, PCH$_2$), 20.88 (Me(p-Tolyl)), 20.85 (Me(p-Tolyl)), 10.23 (5C, C_5Me_5). *$^{31}P\{^1H\}$ NMR (161.98 MHz, THF-d_8, RT, ppm):* δ = 27.88 (s).

Synthesis and characterization of the metallated complexes [Cp*M(κ^3*C,N,P*-H_2L_{-H})][SbF$_6$] (M = Rh, 7; Ir, 8)

In a sealed NMR tube, 38.2 mg (0.040 mmol) of complex **3** or 40.1 mg (0.040 mmol) of complex **4** were dissolved in 0.5 mL of [D$_8$]THF/H_2O (4:1, v/v). The resulting solution was heated at 383 and the reaction was monitored by ^{1}H and ^{31}P NMR. After 24 (Rh) or 32 hours (Ir) of reaction, the resulting yellow solution was vacuum-evaporated until dryness. Pure complexes **7** and **8** were isolated without further purification. Yield: 25.5 mg, 69 % (Rh): 28.5 mg, 74 % (Ir). Crystals suitable for X-ray diffraction analysis were obtained by crystallization from THF/Et$_2$O solutions of **7**. Crystals of **8a** were obtained from CH$_3$OH/acetone/Et$_2$O/n-pentane (1/1/2/2, v/v/v/v) solutions and of **8b** by slow evaporation of CH$_2$Cl$_2$ solutions.

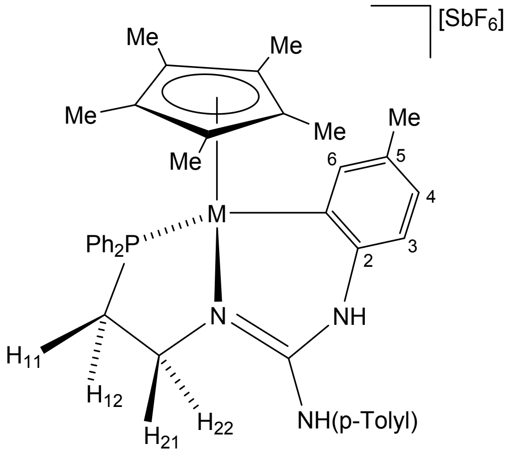

M = Rh (**7**), Ir (**8**)

Compound **7**.

Anal. calcd for $C_{39}H_{44}F_6N_3PRhSb\cdot H_2O$: C, 49.71; H, 4.92; N, 4.46. Found: C, 50.00; H, 4.60; N, 5.05. HRMS (μ-TOF): $C_{39}H_{44}F_6N_3PRhSb$ [M-SbF$_6$]$^+$: calcd 688.2322, found 688.2313. IR (cm $^{-1}$): ν(NH) 3373 (w), 2916 (w); ν(C=N) 1614 (m); ν(SbF$_6$) 654 (s). *^1H NMR (400.16 MHz, THF-d_8, RT, ppm):* δ =

8.41 (s, 1H, $NH_{cis\ to\ Rh}$), 8.36 (s, 1H, $NH_{trans\ to\ Rh}$), 7.74-7.55 (m, 5H, CH(PPh_2)), 7.41-7.19 (m, 5H, CH(PPh_2)), 7.17 (dd, J = 8.8, 0.6 Hz, 2H, CH(p-Tolyl)$_{trans\ to\ Rh}$), 6.96 (dd, J = 8.4, 2.4 Hz, 2H, CH(p-Tolyl)$_{trans\ to\ Rh}$), 6.67 (brs, 1H, 6-CH(p-Tolyl)), 6.53 (dd, J = 7.8, 1.7 Hz, 1H, 3-CH(p-Tolyl)), 6.49 (d, J = 7.8 Hz, 1H, 4-CH(p-Tolyl), 4.03 (m, 1H, H_{22}), 3.69 (m, 1H, H_{21}), 2.62 (m, 1H, H_{11}), 2.35 (m, 1H, H_{12}), 2.30 (s, 3H, Me(p-Tolyl)$_{trans\ to\ Rh}$), 1.94 (s, 3H, Me(p-Tolyl)), 1.48 (d, J = 3.0 Hz, 15H, C_5Me_5). *$^{13}C\{^1H\}$ NMR (75.48 MHz, THF-d_8, RT, ppm):* δ = 152.88 (brd, J = 1.4 Hz, C=N), 142.35 (d, J = 6.8 Hz, 6-CH(p-Tolyl)), 141.15 (dd, J = 31.7, 13.8 Hz, CRh), 138.79 (d, J = 1.8 Hz, 2-C(p-Tolyl)), 138.63 (C(p-Tolyl)$_{trans\ to\ Rh}$), 133.94 (5-C(p-Tolyl)), 133.76 (C(p-Tolyl)$_{trans\ to\ Rh}$), 133.15 (d, J = 8.7 Hz, 2C, CH(PPh_2)), 133.14 (d, J = 41.3 Hz, C(PPh_2)), 133.01 (d, J = 8.0 Hz, 2C, CH(PPh_2)), 132.00 (d, J = 2.3 Hz, CH(PPh_2)), 131.14 (d, J = 2.8 Hz, CH(PPh_2)), 130.97 (dd, J = 47.3, 2.4 Hz, C(PPh_2)), 130.88 (2C, CH(p-Tol)$_{trans\ to\ Rh}$), 129.97 (d, J = 9.8 Hz, 2C, CH(PPh_2)), 129.95 (d, J = 10.2 Hz, 2C, CH(PPh_2)), 125.33 (3-CH(p-Tolyl)), 120.92 (2C, CH(p-Tolyl)$_{trans\ to\ Rh}$), 116.09 (4-CH(p-Tolyl)), 100.77 (brs, 5C, C_5Me$_5$), 52.72 (NCH_2), 34.23 (d, J = 34.3 Hz, PCH_2), 20.88 (Me(p-Tolyl)$_{trans\ to\ Rh}$), 20.73 (Me(p-Tolyl)), 9.42 (5C, C_5Me_5). *$^{31}P\{^1H\}$ NMR (121.42 MHz, THF-d_8, RT, ppm):* δ = 46.45 (d, J = 153.3 Hz).

Compound **8**

Anal. calcd for $C_{39}H_{44}F_6IrN_3PSb\cdot 3H_2O$: C, 43.87; H, 4.72; N, 3.94. Found: C, 43.42; H, 4.58; N, 3.46. HRMS (μ-TOF): $C_{39}H_{44}F_6IrN_3PSb$ [M-SbF_6]$^+$: calcd 778.2899, found 778.2097. IR (cm^{-1}): ν(NH) 3375 (w); ν(N=C) 1625 (m); ν(SbF_6) 654 (s). *^1H NMR (400.16 MHz, THF-d_8, RT, ppm):* δ = 8.43 (s, 1H, $NH_{trans\ to\ Ir}$), 8.34 (s, 1H, $NH_{cis\ to\ Ir}$), 7.65-7.35 (m, 10H, CH(PPh_2)), 7.16 (brd, J = 8.2 Hz, 2H, CH(p-Tolyl)$_{trans\ to\ Ir}$), 6.98 (brd, J = 8.4, 2.4 Hz, 2H, CH(p-Tolyl)$_{trans\ to\ Ir}$), 6.77-6.51 (m, 2H, 6-CH(p-Tolyl, 4-CH(p-Tolyl)), 6.46 (dd, J = 7.8, 1.3 Hz, 1H, 3-CH(p-Tolyl)), 4.14 (ddd, J = 34.4, 12.2, 6.4 Hz, 1H, H_{22}), 3.59 (m, 1H, H_{21}), 2.46 (ptd, J = 12.2, 4.1 Hz, 1H, H_{11}), 2.30 (s, 3H, Me(p-Tolyl)$_{trans\ to\ Ir}$), 2.18 (m, 1H, H_{12}), 1.83 (s, 3H, Me(p-Tolyl)), 1.52 (d, J = 2.0 Hz, 15H, C_5Me_5). *$^{13}C\{^1H\}$ NMR (75.48 MHz, THF-d_8, RT, ppm):* δ = 153.22 (brd, J = 2.5 Hz, C=N), 142.79 (d, J = 4.4 Hz, 6-CH(p-Tolyl)), 139.71

(brs, 2-C(*p*-Tolyl)), 138.65 (C(*p*-Tolyl)$_{trans\text{ to Ir}}$), 134.01 (d, *J* = 52.2 Hz, C(PPh_2)), 133.92 (5-C(*p*-Tolyl)), 133.67 (C(*p*-Tolyl)$_{trans\text{ to Ir}}$), 133.43 (d, *J* = 9.1 Hz, 2C, CH(PPh_2)), 133.05 (d, *J* = 9.9 Hz, 2C, CH(PPh_2)), 131.98 (d, *J* = 2.5 Hz, CH(PPh_2)), 131.37 (d, *J* = 2.8 Hz, CH(PPh_2)), 130.90 (2C, CH(*p*-Tolyl)$_{trans\text{ to Ir}}$), 129.75 (d, *J* = 10.3 Hz, 2C, CH(PPh_2)), 129.34 (d, *J* = 10.4 Hz, 2C, CH(PPh_2)), 128.65 (d, *J* = 56.7 Hz, C(PPh_2)), 125.36 (3-CH(*p*-Tolyl)), 123.61 (d, *J* = 9.1 Hz, CIr), 121.23 (2C, CH(*p*-Tolyl)$_{trans\text{ to Ir}}$), 115.73 (4-CH(*p*-Tolyl)), 95.45 (d, *J* = 2.9 Hz, 5C, C_5Me_5), 57.33 (brd, *J* = 2.1 Hz, NCH_2), 35.29 (d, *J* = 38.8 Hz, PCH_2), 20.93 (Me(*p*-Tolyl)$_{trans\text{ to Ir}}$), 20.68 (Me(*p*-Tolyl)), 9.19 (5C, C_5Me_5). *$^{31}P\{^1H\}$ NMR (161.98 MHz, THF-d_8, RT, ppm): δ* = 19.01 (s).

3. ^{1}H, ^{31}P{^{1}H} and ^{13}C{^{1}H} spectra for the complexes 2, 4, 6, 7 and 8

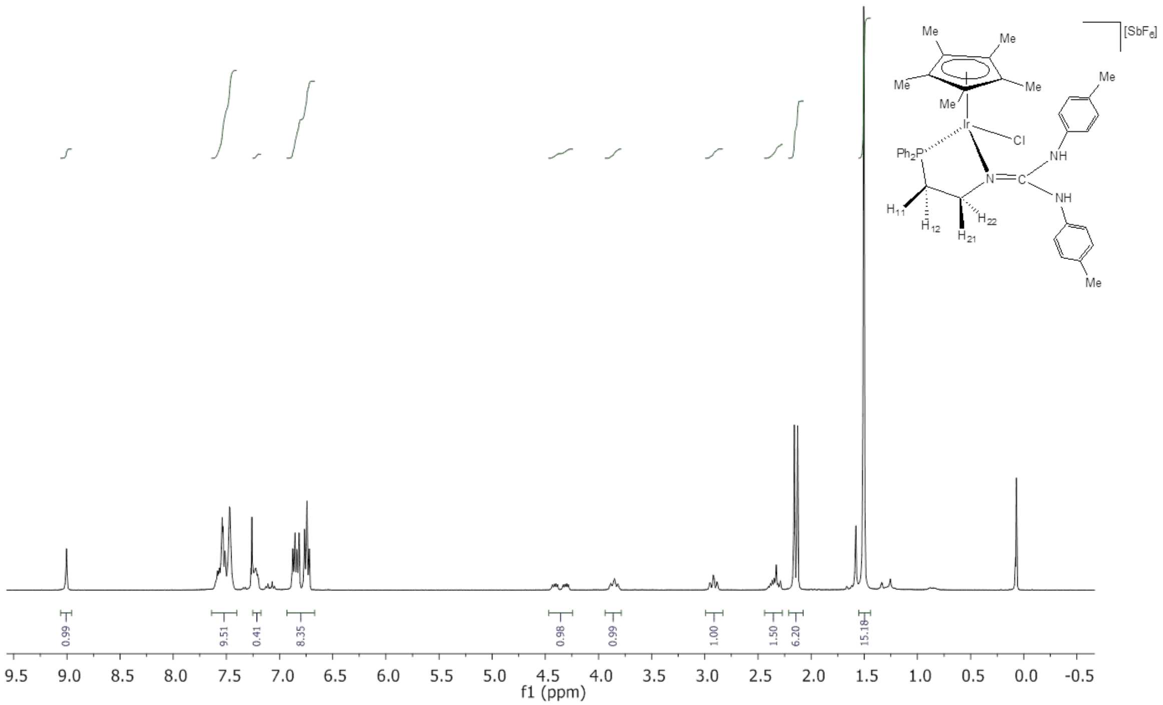

Figure S1. ^{1}H NMR ($CDCl_3$, RT) spectrum of **2**.

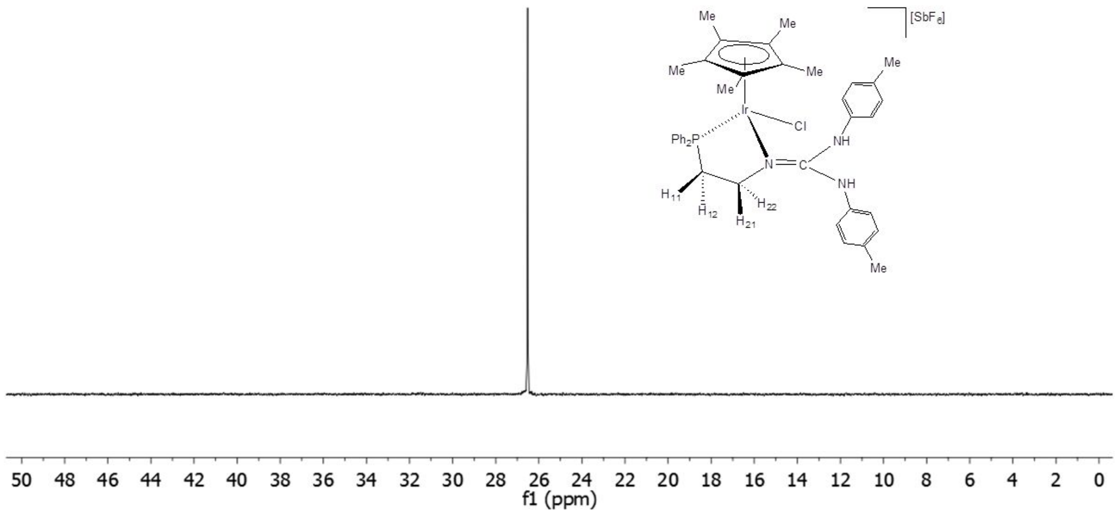

Figure S2. ^{31}P{^{1}H} ($CDCl_3$, RT) spectrum of **2**.

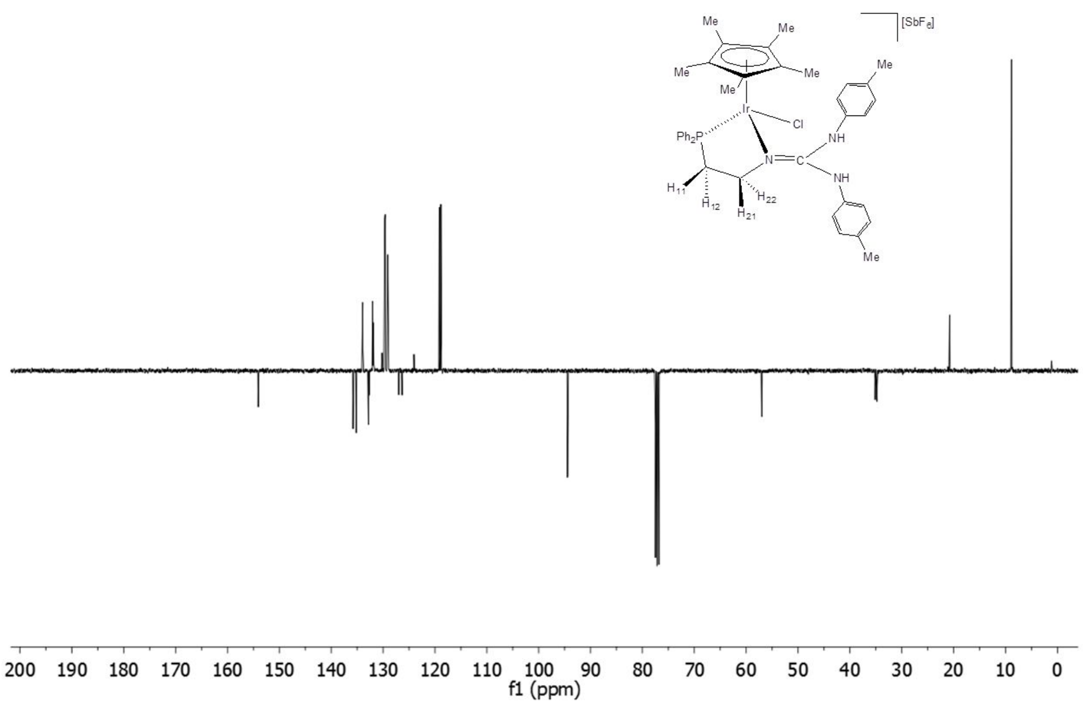

Figure S3. $^{13}C\{^{1}H\}$ ($CDCl_3$, RT) spectrum of **2**.

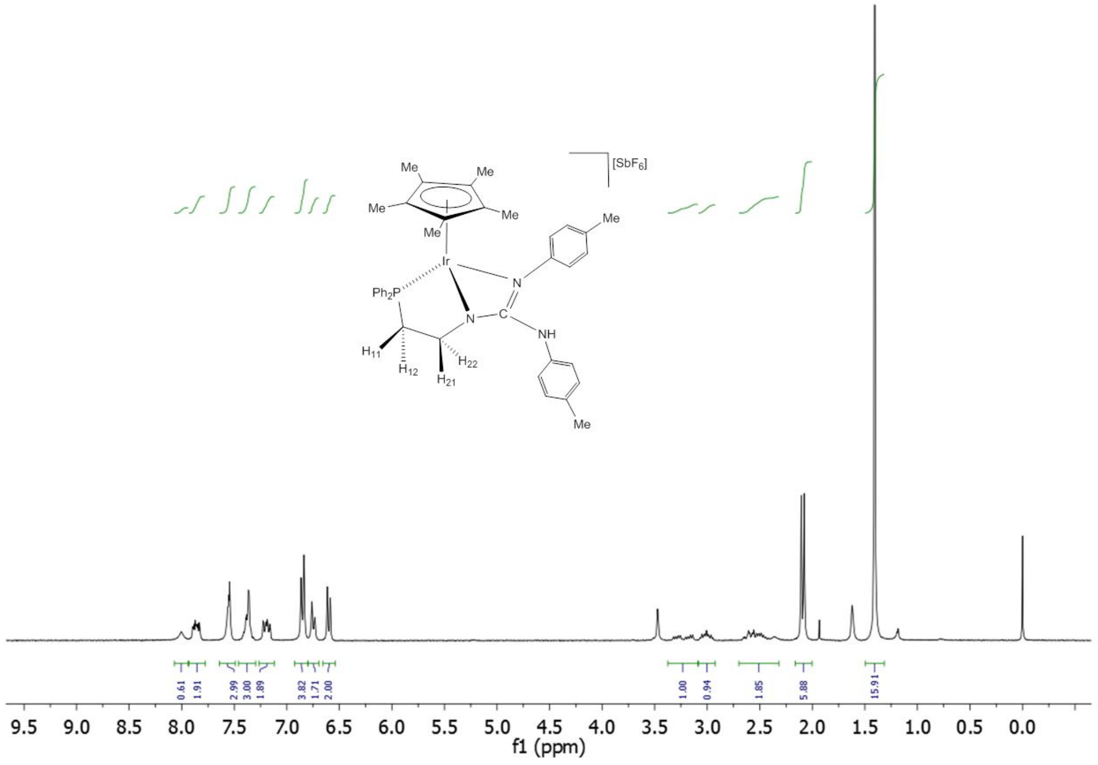

Figure S4. ^{1}H NMR (THF-d_8, RT) spectrum of **4**.

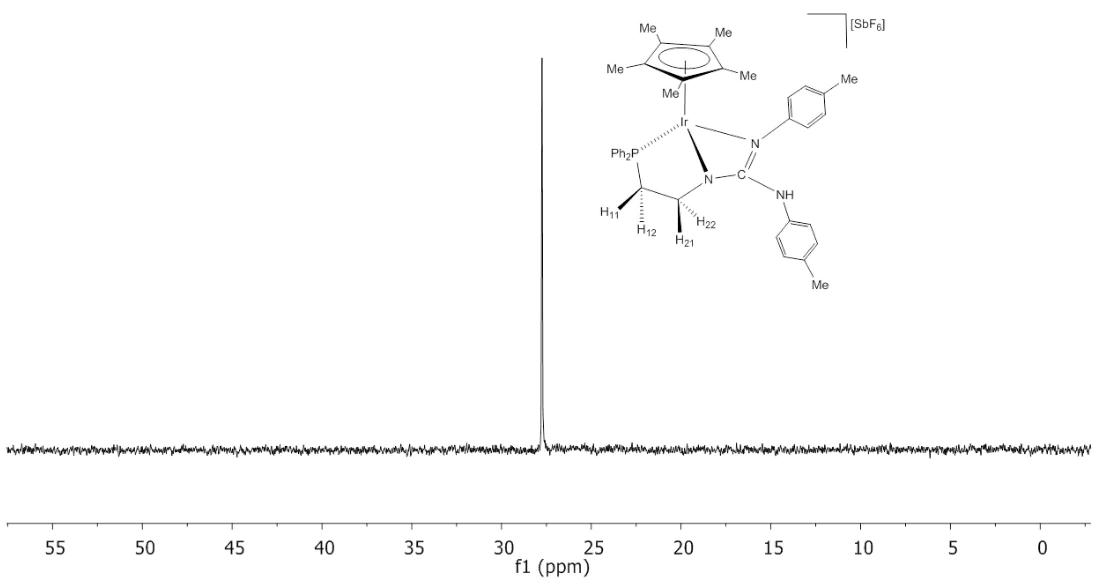

Figure S5. $^{31}P\{^{1}H\}$ (THF-d_8, RT) spectrum of **4**.

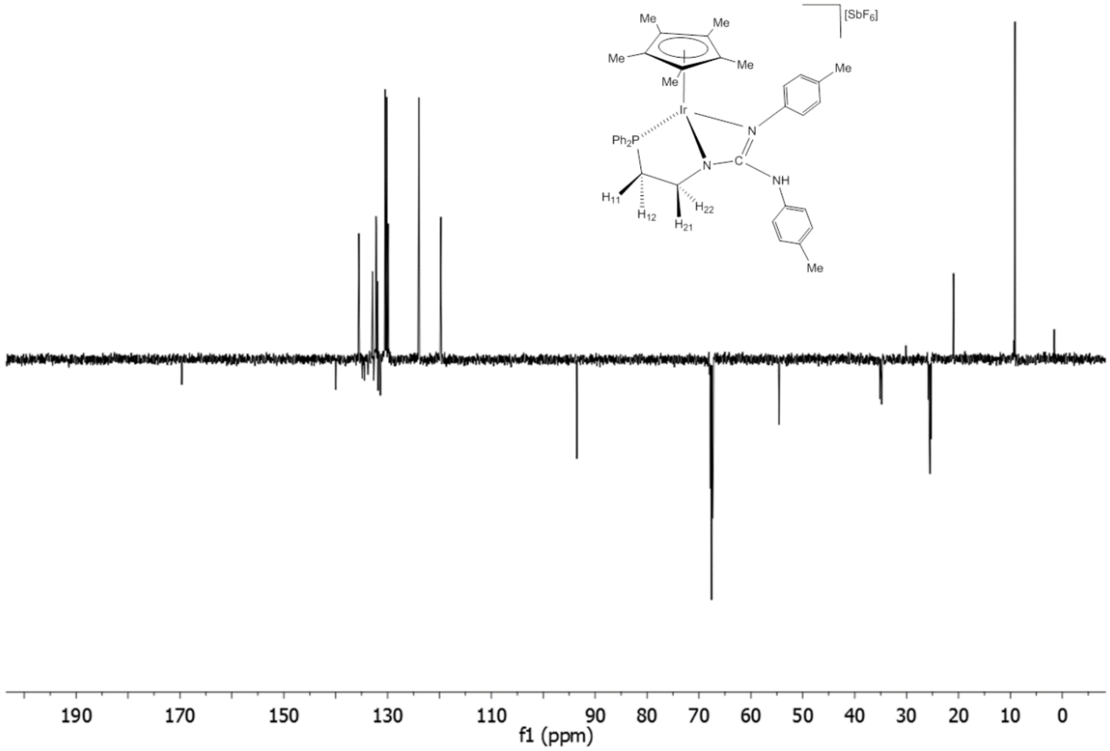

Figure S6. $^{13}C\{^{1}H\}$ (THF-d_8, RT) spectrum of **4**.

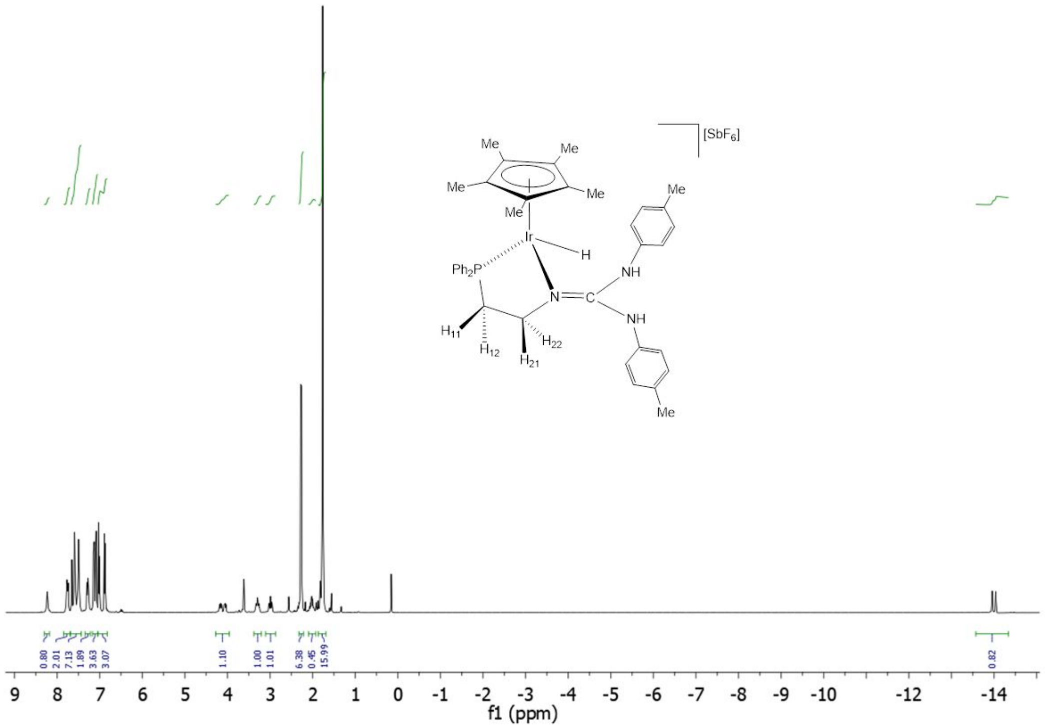

Figure S7. ^{1}H NMR (THF-d_8, RT) spectrum of **6**.

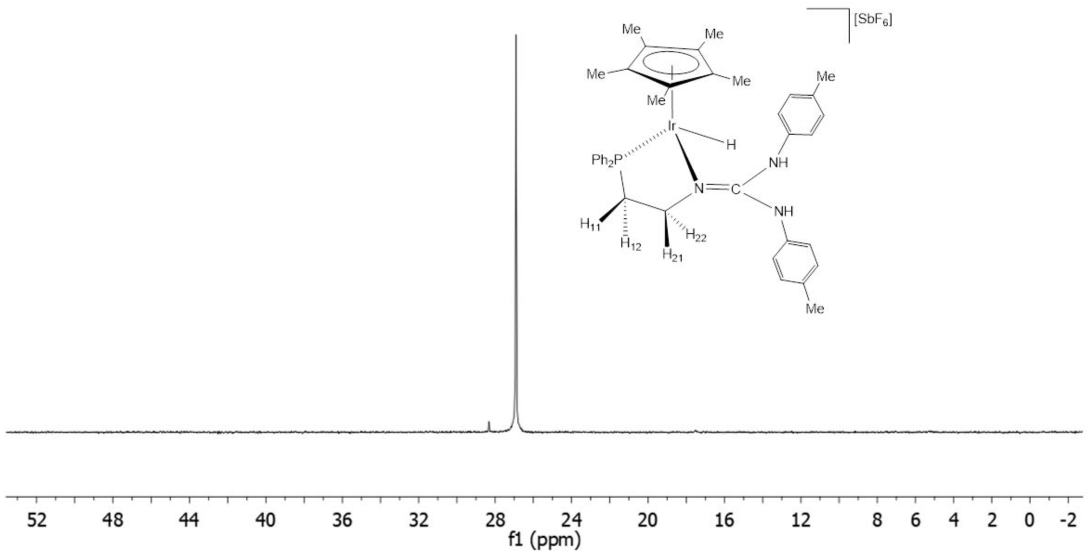

Figure S8. $^{31}P\{^{1}H\}$ (THF-d_8, RT) spectrum of **6**.

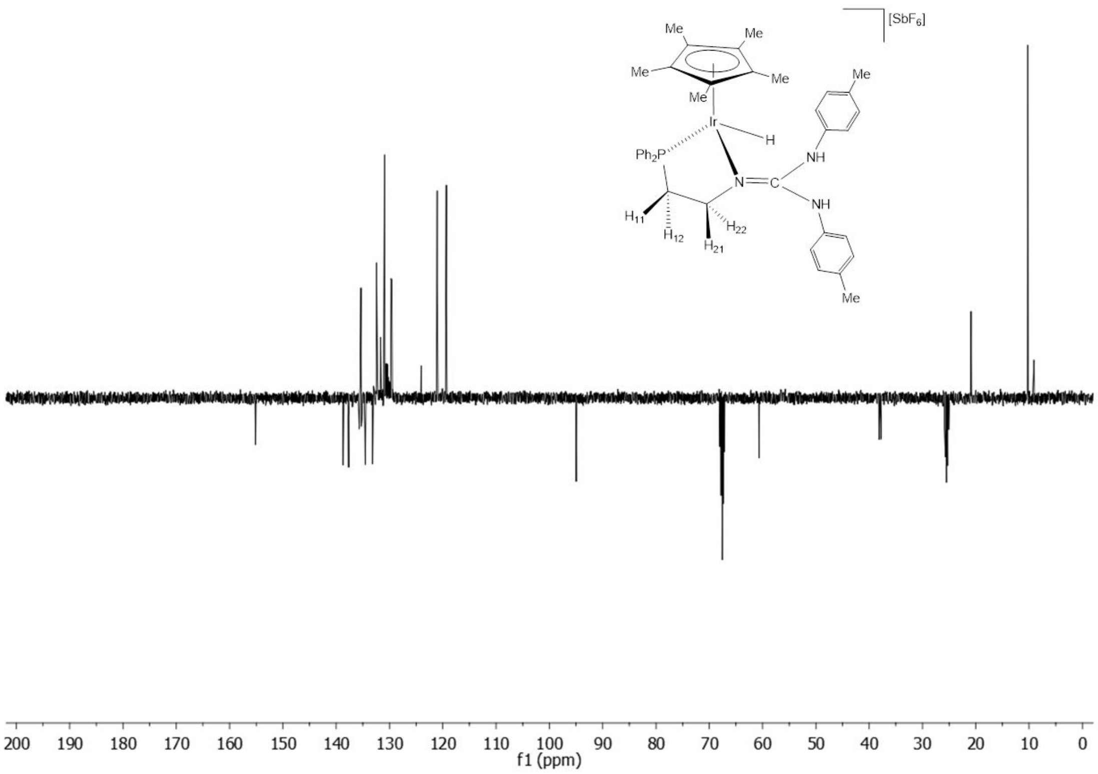

Figure S9. $^{13}C\{^{1}H\}$ (THF-d_8, RT) spectrum of **6**.

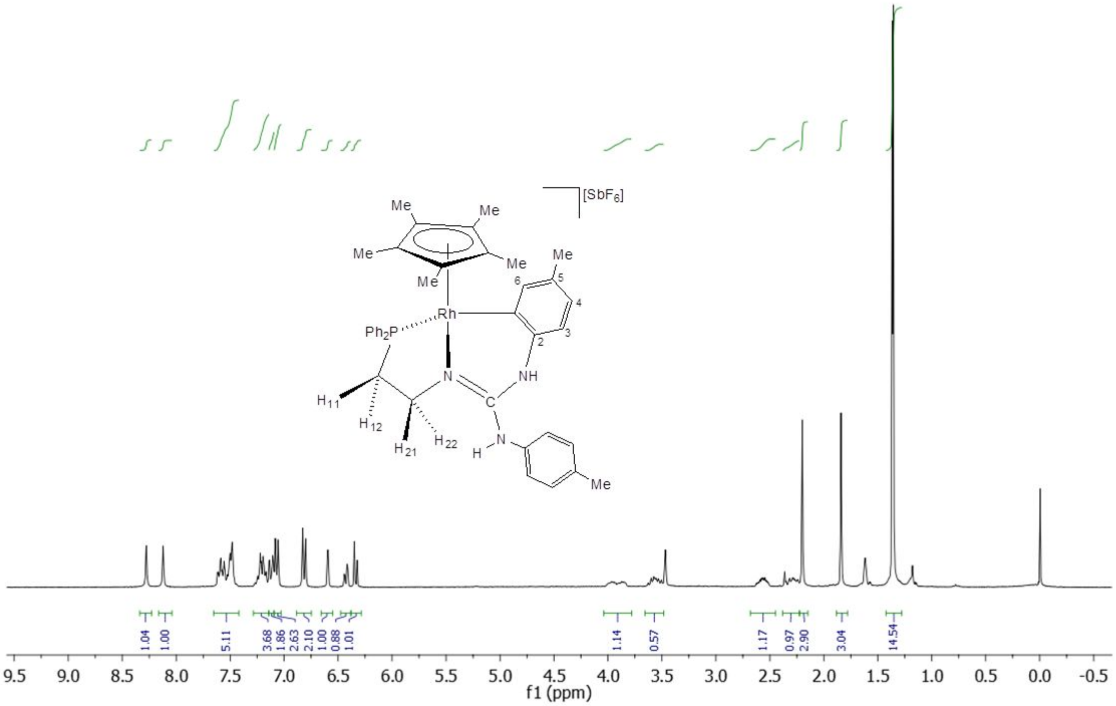

Figure S10. ^{1}H NMR (THF-d_8, RT) spectrum of **7.**

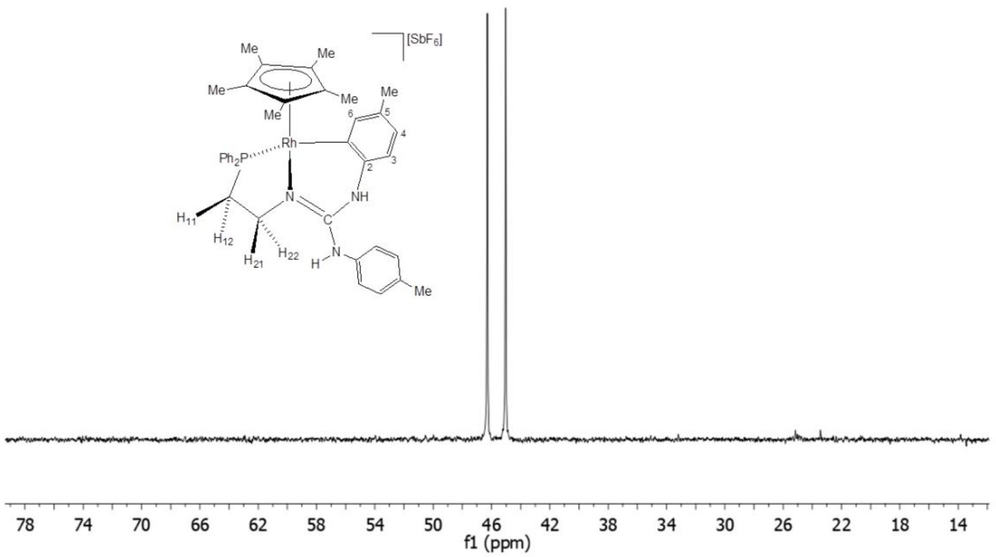

Figure S11. $^{31}P\{^{1}H\}$ (THF-d_8, RT) spectrum of 7**.**

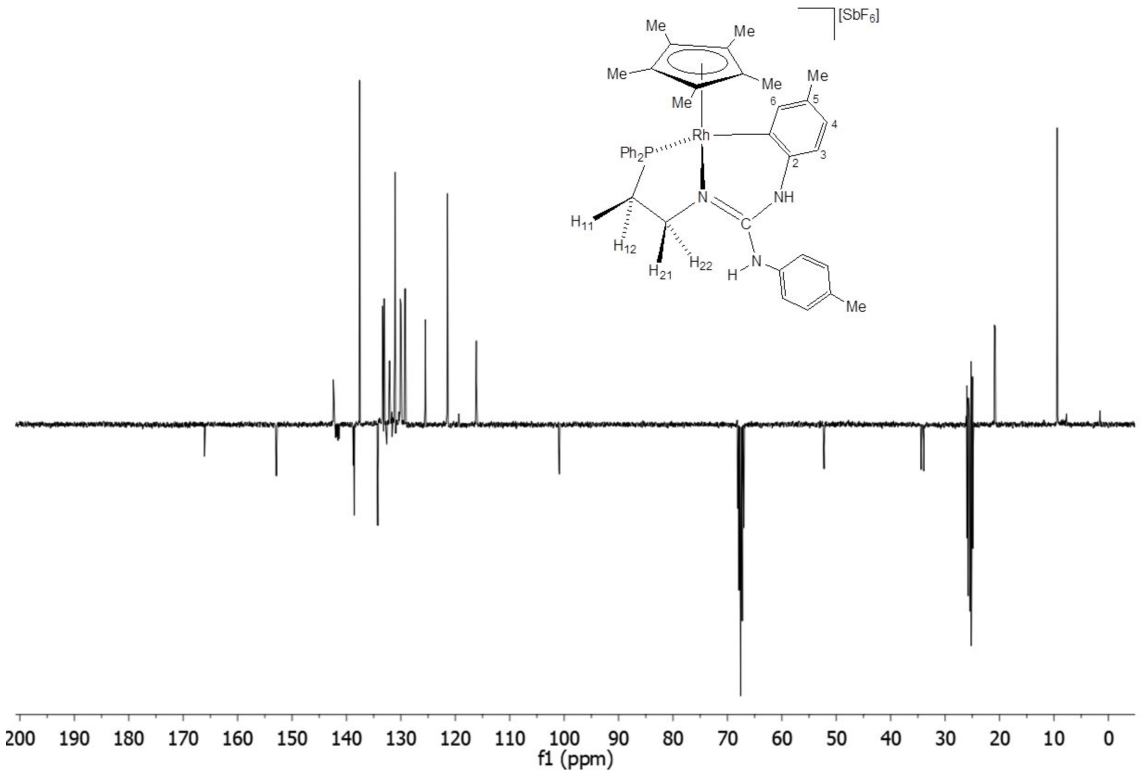

Figure S12. $^{13}C\{^{1}H\}$ (THF-d_8, RT) spectrum of **7.**

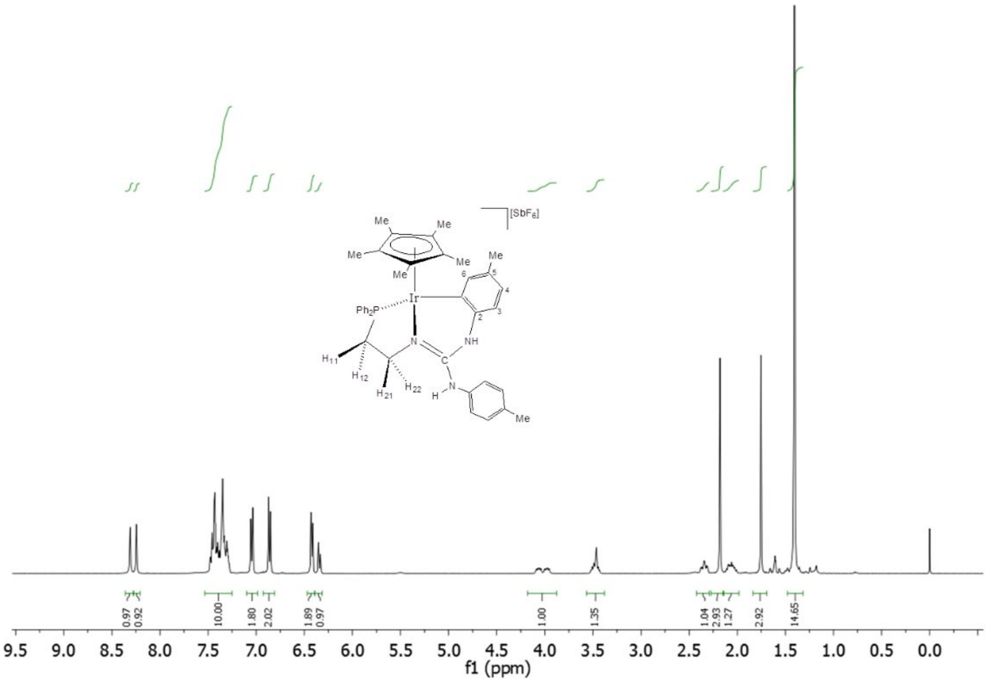

Figure S13. ^{1}H NMR (THF-d_8, RT) spectrum of **8.**

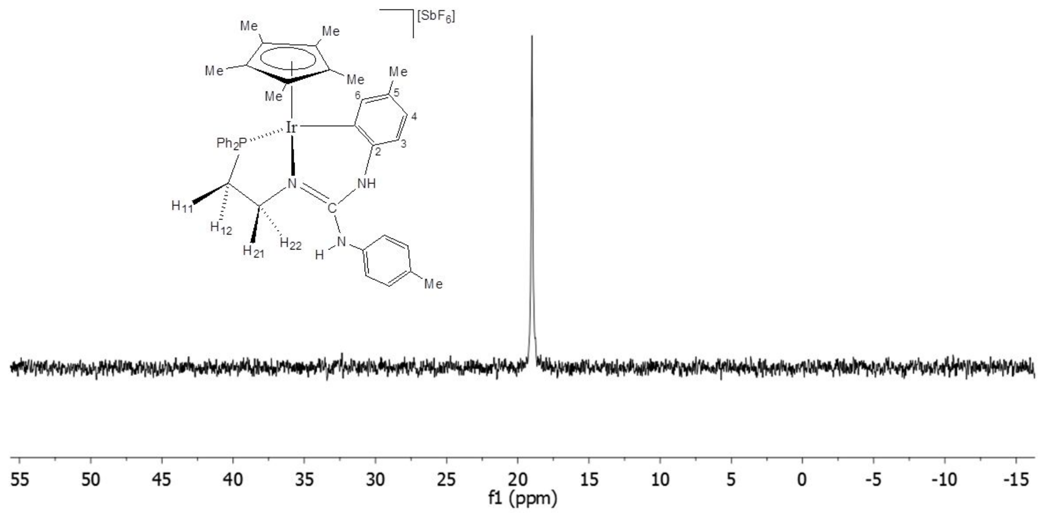

Figure S14. $^{31}P\{^{1}H\}$ (THF-d_8, RT) spectrum of **8.**

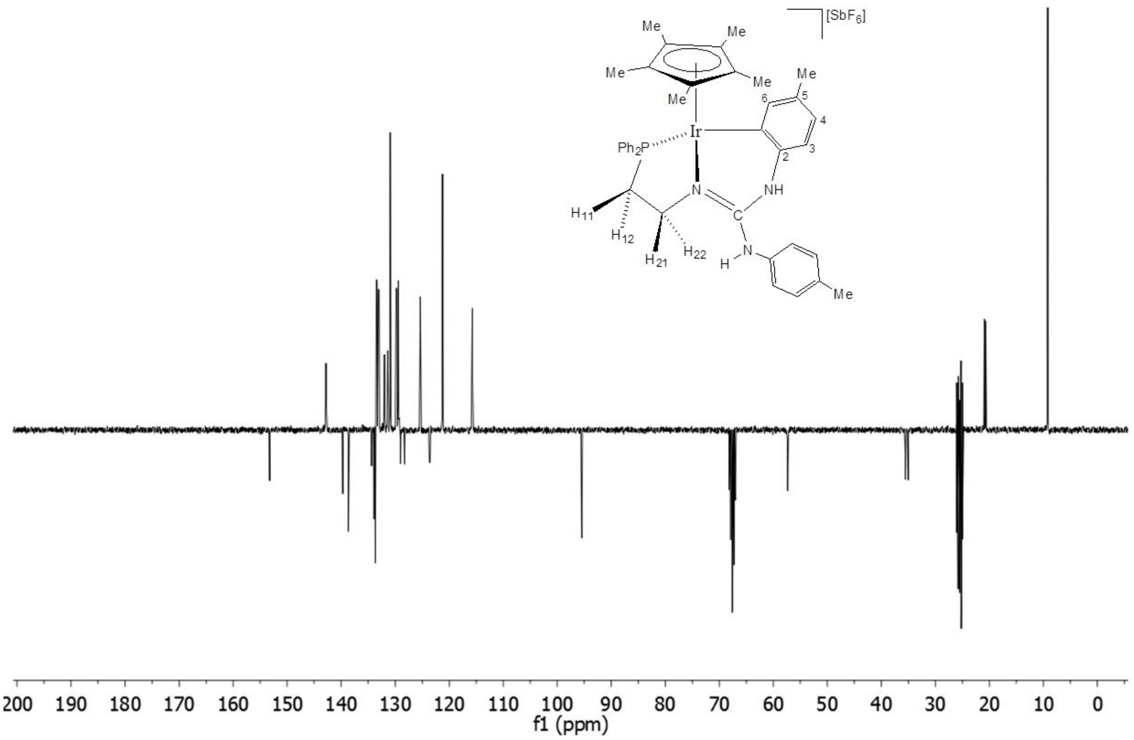

Figure S15. $^{13}C\{^{1}H\}$ (THF-d_8, RT) spectrum of **8.**

4. CD of complexes 1 and 2

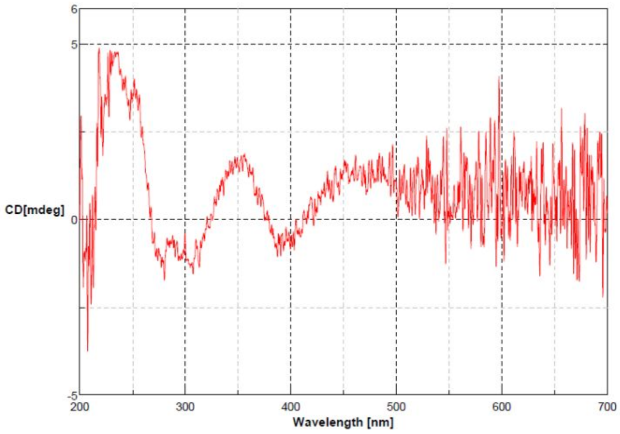

Figure S16. CD spectra of the measured crystal by X-ray diffraction corresponding to S_{Rh}-**1** in CH_2Cl_2.

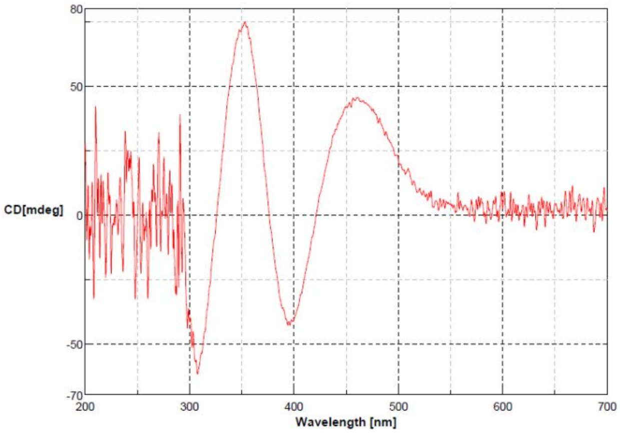

Figure S17. CD spectra of S_{Rh}-**1** (4.39×10^{-4} M) in CH_2Cl_2.

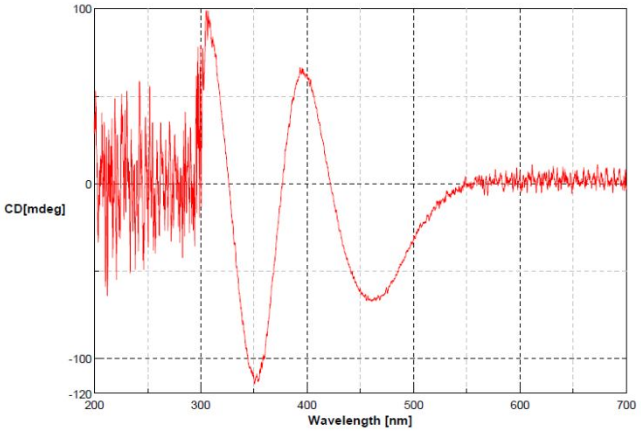

Figure S18. CD spectra of R_{Rh}-**1** (5.58×10^{-4} M) in CH_2Cl_2.

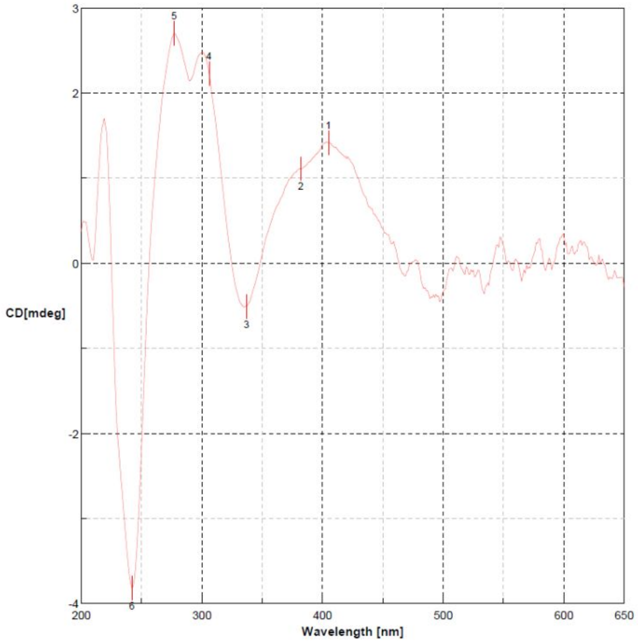

Figure S19. CD spectra of the measured crystal by X-ray diffraction corresponding to S_{Ir}-**2** in CH_2Cl_2.

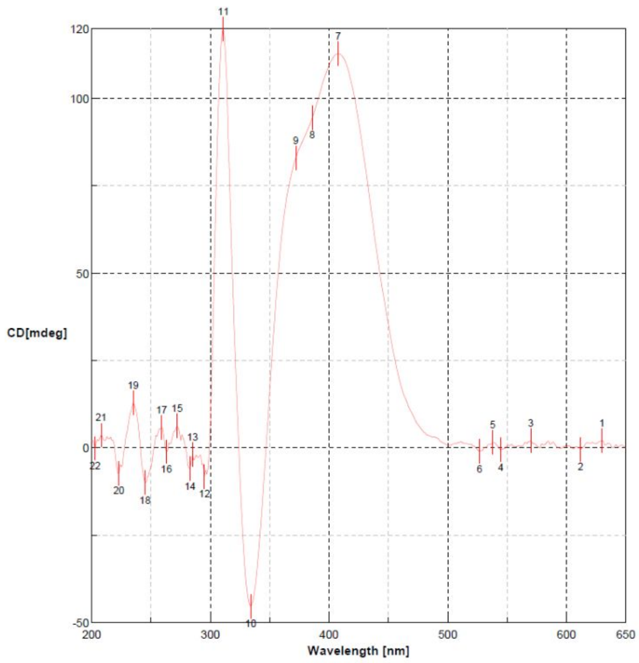

Figure S20. CD spectra of S_{Ir}-**2** (5.95×10^{-4} M) in CH_2Cl_2.

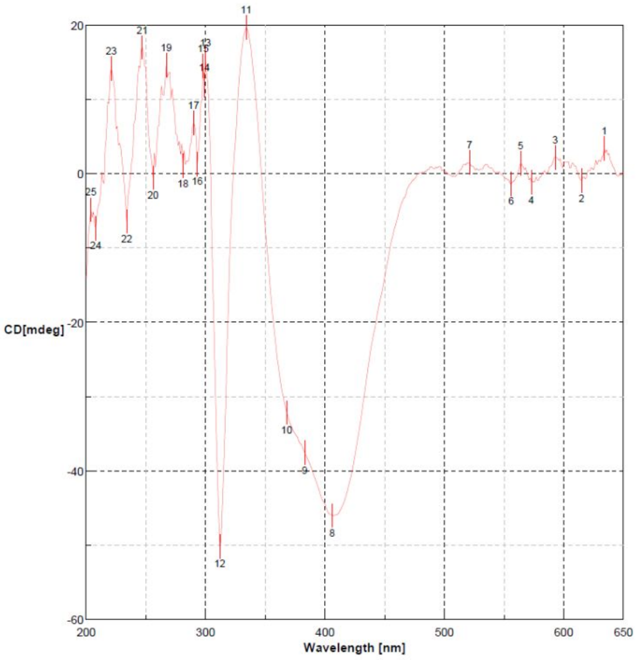

Figure S21. CD spectra of R_{Ir}-**2** (3.81×10^{-4} M) in CH_2Cl_2.

5. Dehydrogenation reaction of complexes 5 and 6

In a high-pressure NMR tube, solution of the complex **5** (10.1 mg, 0.011 mmol) and **6** (24.5 mg, 0.024 mmol) in THF-d_8 was monitorized by NMR. After heating the rhodium sample at 393 K for 30 min, the clean conversion to **3** was about 30 %. A clean conversion of about 50% to **4** was observed after heating the iridium sample at 393 K for 2.5 h.

6. Kinetic studies for the H/D exchange at Cp* of complex 3

$[H]_t$	t (s)	$\ln[H]_t$
258,1826967	886	5,55366746
252,413251	1596	5,53106763
245,9226245	2194	5,50501695
235,2852089	2797	5,46079843
230,5975343	3396	5,44067392
223,7463175	3996	5,4105129
214,7315585	4598	5,36938868
203,7335526	5203	5,31681303
199,9473538	5806	5,2980541
192,0143659	6413	5,25757019
187,5069864	7015	5,23381611
182,6390166	7618	5,20751162
177,4104564	8225	5,17846601
172,9030769	8830	5,15273119
163,3474324	9445	5,09587942

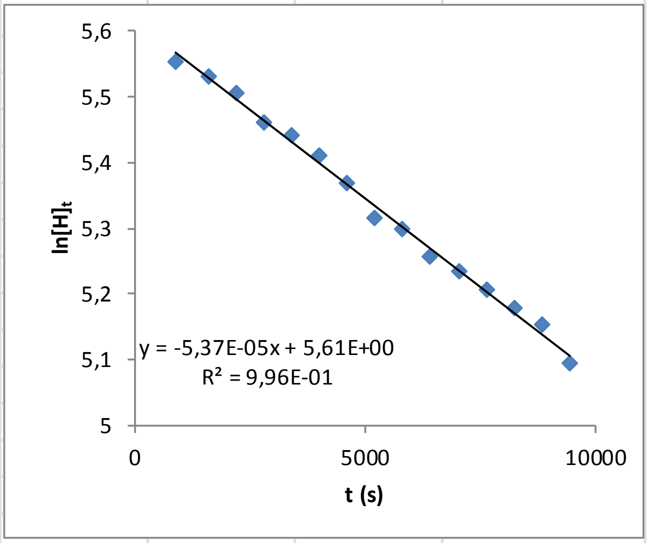

Figure S22. Kinetic experiment at 298 K: **3** (18.03 mM) in 0.35/0.10 mL of THF-d_8/CD_3OD. k_{obs} = $5.37 \pm 0.1 \times 10^{-5}$ s^{-1}.

$[H]_t$	t (s)	$\ln[H]_t$
252,605566	334	5,531829244
250,115088	670	5,521921166
244,066786	1003	5,497441902
238,018484	1334	5,472348334
231,258617	1667	5,443536637
222,897728	2001	5,406713048
218,450447	2333	5,386559202
212,402145	2670	5,358481387
206,531734	3006	5,330454075
203,507583	3339	5,315703265
198,348737	3676	5,290026777
191,410978	4014	5,254422834
185,540567	4352	5,223273548
181,626960	4689	5,201954911
176,646005	5024	5,174147757
173,977636	5358	5,158926763
170,419811	5695	5,138264871
164,905183	6035	5,105370659
161,169467	6371	5,082456399

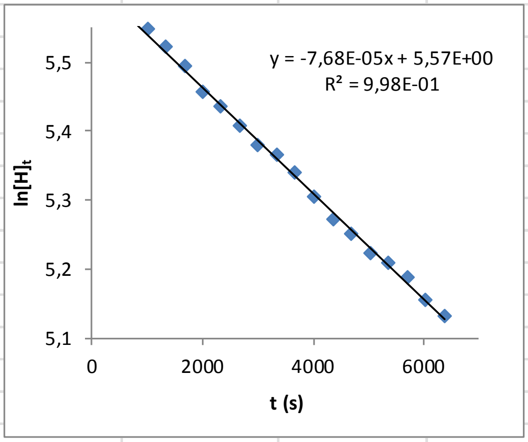

Figure S23. Kinetic experiment at 303 K: **3** (17.79 mM) in 0.35/0.10 mL of THF-d_8/CD_3OD. k_{obs} = $7.68 \pm 0.08 \times 10^{-5}$ s^{-1}.

$[H]_t$	t (s)	$\ln[H]_t$
267,288805	266	5,588329741
255,442210	603	5,5429962
244,150924	803	5,497786575
238,597832	1002	5,474779425
229,342680	1203	5,435217304
223,419382	1406	5,409050643
216,200363	1596	5,376205585
209,721756	1774	5,345781683
199,726192	1971	5,296947387
195,653925	2173	5,276347408
189,360421	2374	5,243652188
179,549959	2576	5,190453493
177,513826	2774	5,179048497
169,554394	2974	5,133173787

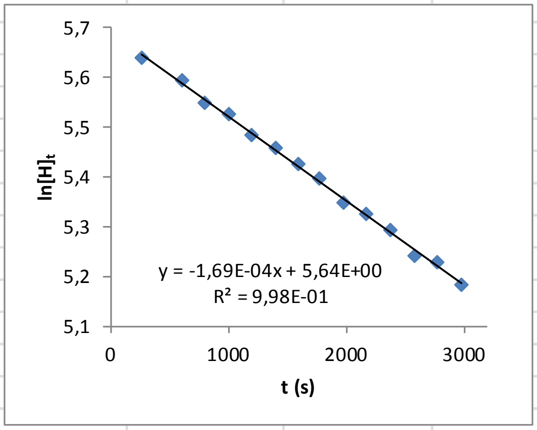

Figure S24. Kinetic experiment at 313 K: **3** (18.51 mM) in 0.35/0.10 mL of THF-d_8/CD_3OD. k_{obs} = $1.69 \pm 0.02 \times 10^{-4}$ s^{-1}.

$[H]_t$	t (s)	$\ln[H]_t$
268,807131	199	5,59399414
248,7813849	399	5,51657454
233,56952	599	5,45347976
215,4693264	797	5,37281856
199,10213	996	5,29381791
185,0455967	1194	5,22060226

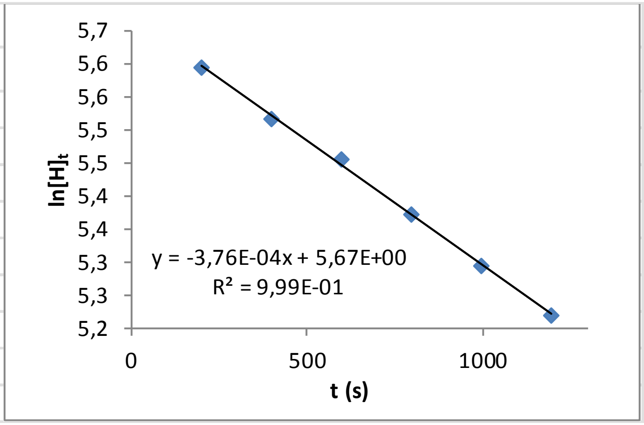

Figure S25. Kinetic experiment at 323 K: **3** (19.26 mM) in 0.35/0.10 mL of THF-d_8/CD_3OD. k_{obs} = $3.76 \pm 0.06 \times 10^{-4}$ s^{-1}.

T	k_{obs} (mM/s)	(1/T)	ln(k_{obs}/T)
298	5,37E-05	0,003355705	-15,529191
303	7,68E-05	0,00330033	-15,188039
313	1,69E-04	0,003194888	-14,431815
323	3,76E-04	0,003096	-13,663574

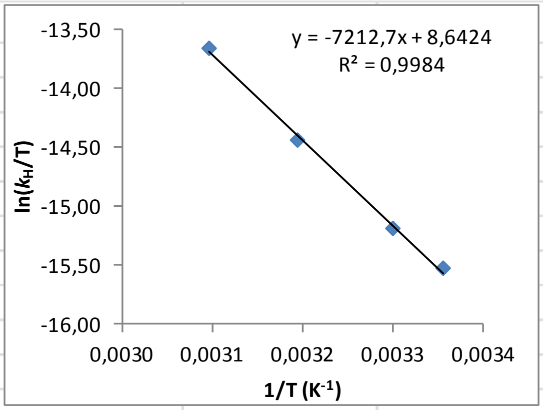

Figure S26. Eyring analysis for the H/D exchange at Cp* of complex **3** with CD_3OD. ΔG≠ = 23.1 ± 0.8 kcal/mol; ΔH≠ = 14.33 ± 0.40 kcal/mol; ΔS≠ = -30.04 ± 1.30 u.

$[H]_t$	t (s)	$ln[H]_t$
282,300422	475	5,642971831
276,001630	743	5,620406771
270,466327	1014	5,600147608
265,121898	1279	5,580189711
259,395723	1546	5,558354782
254,242165	1813	5,538287219
249,279480	2082	5,518574678
245,462030	2347	5,503142273
240,881091	2614	5,484303411
237,827131	2880	5,471544068
233,246191	3148	5,452094508
230,001358	3416	5,438085214
225,993036	3682	5,420504184
221,602968	3949	5,40088735
217,976391	4216	5,384386759
213,777196	4485	5,364934333
210,150619	4755	5,347824505

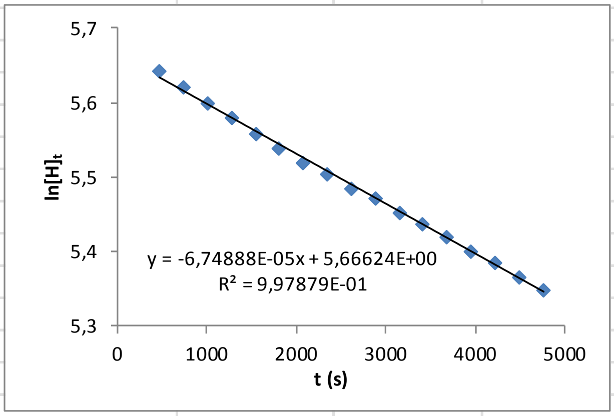

Figure S27. Kinetic experiment at 303 K: **3** (19.09 mM) in 0.35/0.10 mL of THF-d_8/CH_3OD. k_{obs} = 6.75 ± 0.08 × 10^{-5} s^{-1}.

$[H]_t$	t (s)	$\ln[H]_t$
273,735680	220	5,612162969
266,413532	354	5,585049734
258,715890	489	5,555730508
248,953026	622	5,517264228
238,251425	759	5,473326525
228,864056	892	5,433128186
221,729656	1025	5,401458873
211,966792	1160	5,356429621
203,330413	1294	5,314832304

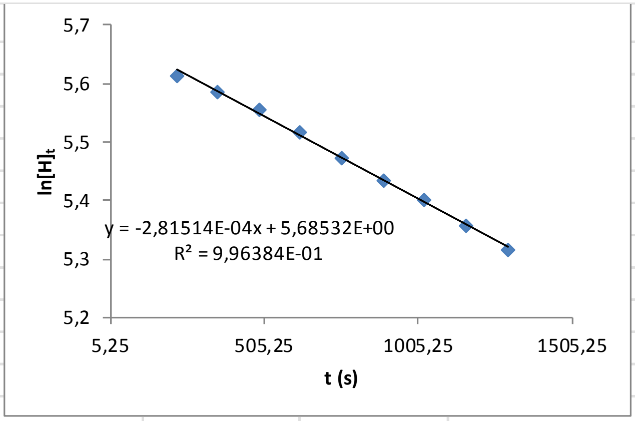

Figure S28. Kinetic experiment at 313 K: **3** (18.77 mM) in 0.35/0.10 mL of THF-d_8/CH_3OD. k_{obs} = $2.82 \pm 0.06 \times 10^{-4}$ s^{-1}.

$[H]_t$	t (s)	$\ln[H]_t$
269,229743	133	5,59556508
253,271216	232	5,53446091
237,688183	334	5,47095966
223,231635	434	5,40820995
209,338329	522	5,34395174
196,008265	634	5,27815683

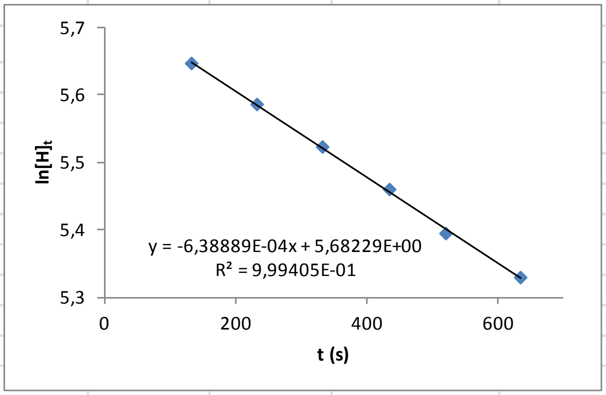

Figure S29. Kinetic experiment at 323 K: **3** (18.77 mM) in 0.35/0.10 mL of THF-d_8/CH_3OD. k_{obs} = $6.39 \pm 0.08 \times 10^{-4}$ s^{-1}.

T	k_{obs} (mM/s)	(1/T)	ln(k_{obs}/T)
303	6,75E-05	0,003300	-15,317282
313	2,82E-04	0,003195	-13,921532
323	6,39E-04	0,003096	-13,133432

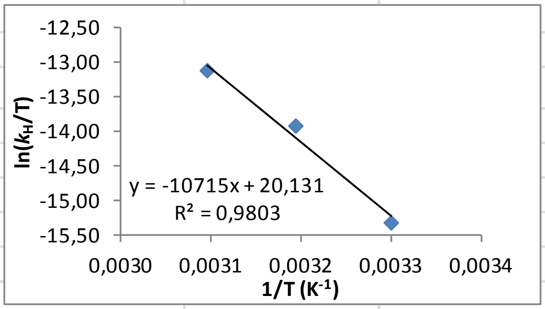

Figure S30. Eyring analysis for the H/D exchange at Cp* of complex **3** with CH_3OD. $\Delta G^{\neq} = 23.4 \pm 0.2$ kcal/mol; $\Delta H^{\neq} = 21 \pm 3$ kcal/mol; $\Delta S^{\neq} = -7 \pm 9$ cal/K·mol

$[H]_t$	t (s)	$\ln[H]_t$
252,713262	1457	5,532255495
244,161501	2805	5,497829897
236,656895	4044	5,46661139
228,105134	5391	5,429806636
218,855270	6739	5,388410643
210,652560	8079	5,350210143
201,577222	9422	5,306172545
192,850936	10767	5,261917536
183,950123	12101	5,214664651
175,747414	14362	5,169047815

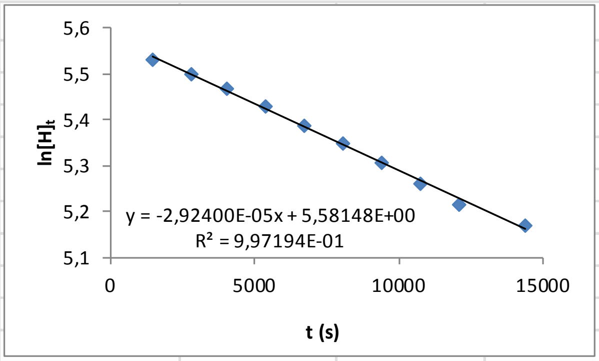

Figure S31. Kinetic experiment at 303 K: **3** (17.45 mM) in 0.35/0.10 mL of THF-d_8/EtOD. $k_{obs} = 2.92 \pm 0.05 \times 10^{-5}$ s^{-1}.

$[H]_t$	t (s)	$ln[H]_t$
259,873144	589	5,560193605
249,857387	1087	5,520890301
240,199334	1589	5,481469138
233,402927	2092	5,452766262
223,387169	2592	5,408906452
215,517645	3091	5,373042788
206,396152	3596	5,329797389
197,990069	4099	5,288216875
190,657104	4603	5,250476547
183,324138	5103	5,211255833

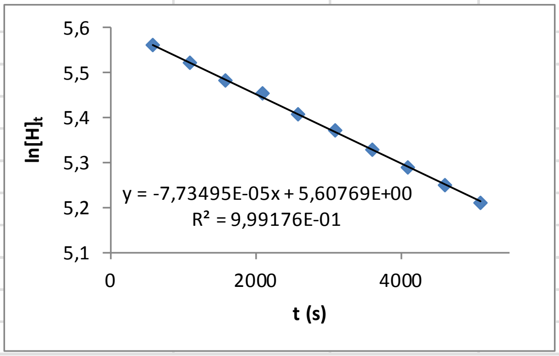

Figure S32. Kinetic experiment at 313 K: **3** (17.89 mM) in 0.35/0.10 mL of THF-d_8/EtOD. k_{obs} = 7.73 ± 0.09 × 10^{-5} s^{-1}.

$[H]_t$	t (s)	$ln[H]_t$
276,7651199	311	5,6231692
267,60324	544	5,58950544
256,9143802	777	5,54874288
246,7981379	1010	5,50857075
238,7814931	1242	5,47554888
230,1922307	1475	5,43891475
219,5033709	1715	5,39136759
211,2958536	1951	5,3532593
199,6526313	2286	5,29657901

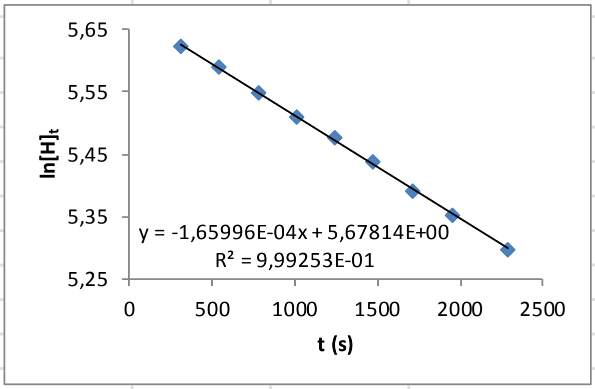

Figure S33. Kinetic experiment at 323 K: **3** (19.09 mM) in 0.35/0.10 mL of THF-d_8/EtOD. k_{obs} = 1.66 ± 0.02 × 10^{-4} s^{-1}.

T	k_{obs} (mM/s)	(1/T)	ln(k_{obs}/T)
303	2,92E-05	0,003300	-16,153706
313	7,73E-05	0,003195	-15,213380
323	1,66E-04	0,003096	-14,481199

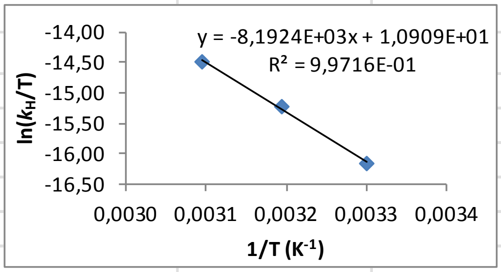

Figure S34. Eyring analysis for the H/D exchange at Cp* of complex **3** with EtOD. $\Delta G^{\neq}$ = 23 ± 2 kcal/mol; $\Delta H^{\neq}$ = 16.3 ± 0.9 kcal/mol; $\Delta S^{\neq}$ = -26 ± 3 cal/K·mol.

$[H]_t$	t (s)	$\ln[H]_t$
292,727253	7295	5,679241299
281,621070	13114	5,640562444
273,291433	18934	5,610538745
258,020431	24736	5,553038772
248,897495	30538	5,517041145
238,187961	36332	5,473060115
230,453298	42129	5,440048231

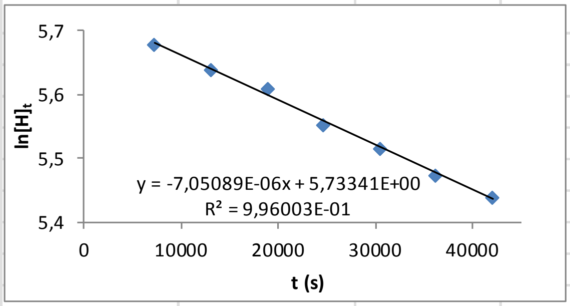

Figure S35. Kinetic experiment at 303 K: **3** (19.83 mM) in 0.35/0.10 mL of THF-d_8/*i*PrOD. k_{obs} = 7.1 ± 0.2 × 10^{-6} s^{-1}.

$[H]_t$	t (s)	$\ln[H]_t$
251,004545	3427	5,525471045
232,681747	6769	5,449671625
219,517795	10118	5,391433298
206,709625	13458	5,331315031
187,141588	18887	5,231865487
172,198724	22552	5,148649181

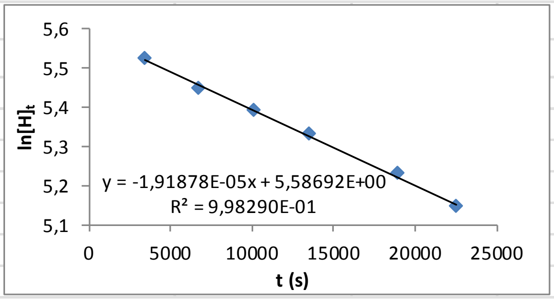

Figure S36. Kinetic experiment at 313 K: **3** (17.79 mM) in 0.35/0.10 mL of THF-d_8/*i*PrOD. k_{obs} = 1.92 ± 0.04 × 10^{-5} s^{-1}.

$[H]_t$	t (s)	$\ln[H]_t$
258,4913621	1430	5,55486228
245,0961514	4117	5,50165059
229,7097608	6804	5,4368166
216,4955665	9494	5,37757007
200,9281596	12162	5,30294743
189,7051453	14832	5,245471
186,0848181	15761	5,22620258

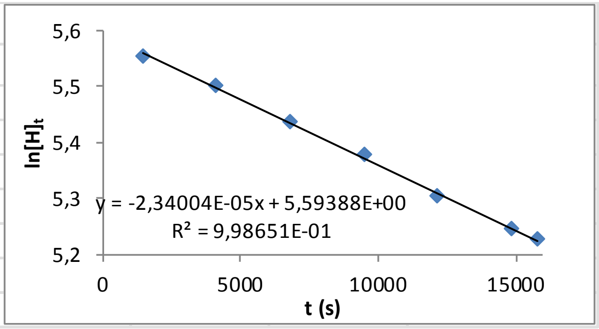

Figure S37. Kinetic experiment at 323 K: **3** (18.10 mM) in 0.35/0.10 mL of THF-d_8/*i*PrOD. k_{obs} = 2.34 ± 0.04 × 10^{-5} s^{-1}.

T	k_{obs} (mM/s)	(1/T)	$\ln(k_{obs}/T)$
303	7,05E-06	0,003300	-17,576090
313	1,92E-05	0,003195	-16,607439
323	2,34E-05	0,003096	-16,440410

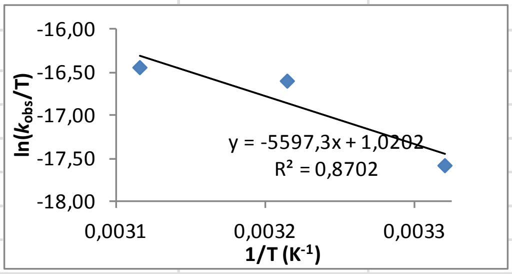

Figure S38. Eyring analysis for the H/D exchange at Cp* of complex **3** with *i*PrOD. ΔG≠ = 24 ± 8 kcal/mol; ΔH≠ = 11 ± 4 kcal/mol; ΔS≠ = -45 ± 14 cal/K·mol.

7. Kinetic studies for acceptorless alcohol dehydrogenation

$[M]_t$	t (s)	$ln[M]_t$
18,6306942	1160	2,92481044
18,5163149	1899	2,91865223
18,4075609	2636	2,9127615
18,2931816	3371	2,9065284
18,1713021	4108	2,89984354
18,0550477	4845	2,8934253
17,929418	5584	2,88644283
17,8262892	6318	2,88067429
17,7175351	7054	2,87455484
17,6012808	7791	2,86797167
17,5037772	8527	2,8624167
17,4025234	9261	2,85661522
17,2918943	9997	2,85023785
17,2112663	10733	2,84556419
17,1325134	11469	2,84097802
16,9956333	12202	2,83295644
16,9318809	12940	2,82919829
16,7931257	13677	2,82096962
16,7293733	14414	2,81716606
16,6449952	15149	2,81210958

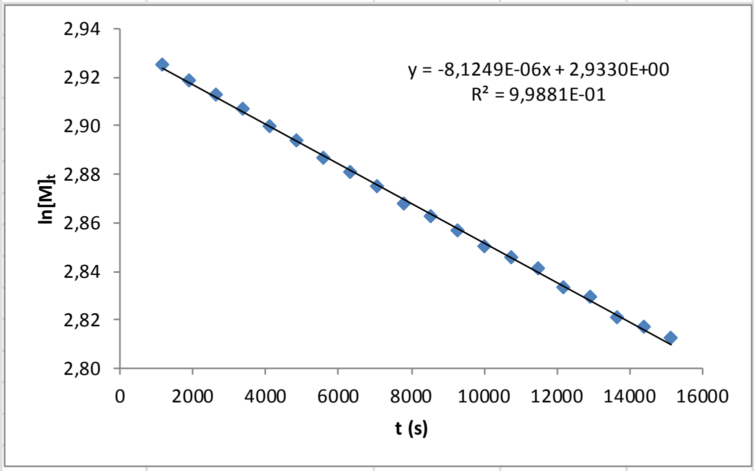

Figure S39. Kinetic experiment at 333 K: **3** (7.80 mg; 0.0084 mmol) and CH_3OH (0.283 mmol) in THF-d_8 (0.45 mL). $k_{obs} = 8.12 \pm 0.07 \times 10^{-6}$ s^{-1}.

$[M]_t$	t (s)	$ln[M]_t$
17,649095	10840	2,870684513
17,564017	11492	2,865852344
17,453597	11893	2,859545784
17,147680	24553	2,841862875
17,006487	30572	2,833594861
16,700569	42612	2,815442813
16,544895	48631	2,806077613
16,398272	54651	2,797175967
16,047100	66691	2,775528167
15,969263	72711	2,77066583
15,752044	78730	2,756970112
15,505861	90774	2,741218107
15,395441	96793	2,734071454

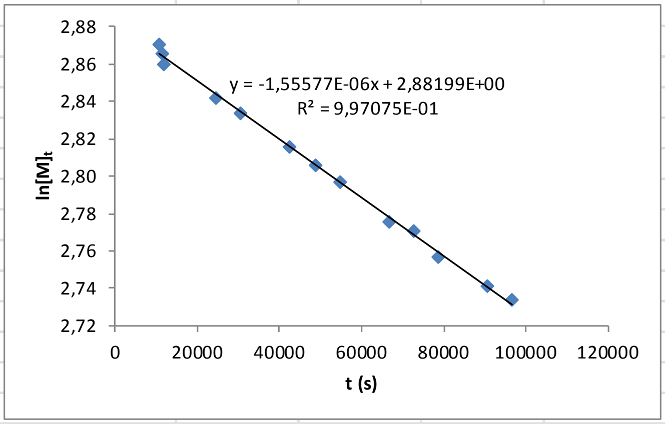

Figure S40. Kinetic experiment at 333 K: **3** (7.53 mg; 0.0081 mmol) and CH_3OD (0.285 mmol) in THF-d_8 (0.45 mL). $k_{obs} = 1.56 \pm 0.03 \times 10^{-6}$ s^{-1}.

$[M]_t$	t (s)	$ln[M]_t$
17,909321	0	2,885321311
17,749928	8611	2,87638147
17,726646	9429	2,875068935
17,697991	10246	2,873451139
17,601281	16551	2,867971673
17,522480	22532	2,863484617
17,420397	28552	2,857641742
17,312941	34573	2,851454242
17,218021	40593	2,845956588
17,128475	46613	2,840742268
16,909981	58654	2,827904041
16,677160	70693	2,814040109
16,508812	82733	2,803894313
16,302855	94774	2,791340248
16,127344	106812	2,780516197

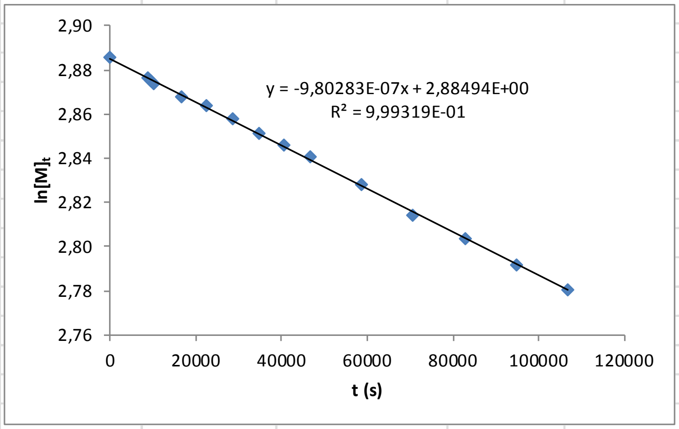

Figure S41. Kinetic experiment at 333 K: **3** (7.45 mg; 0.0081 mmol) and CD_3OH (0.285 mmol) in THF-d_8 (0.45 mL). $k_{obs} = 9.80 \pm 0.07 \times 10^{-7}$ s^{-1}.

$[M]_t$	t (s)	$ln[M]_t$
19,146959	895	2,952143883
19,039532	1534	2,946517424
18,909084	2177	2,93964246
18,820841	2817	2,934964803
18,667373	3451	2,926777263
18,531171	4084	2,919454246
18,393051	4718	2,911972914
18,274114	5357	2,9054855
18,164768	5996	2,899483902
17,986362	6629	2,88961383
17,884690	7265	2,883945063
17,662163	8357	2,871424662
17,566246	8997	2,865979212
17,339882	9487	2,853009146
17,226700	10123	2,84646048
17,088579	10761	2,838410347
17,000335	11400	2,83323307
16,871807	12038	2,825643976
16,756706	12678	2,818798541

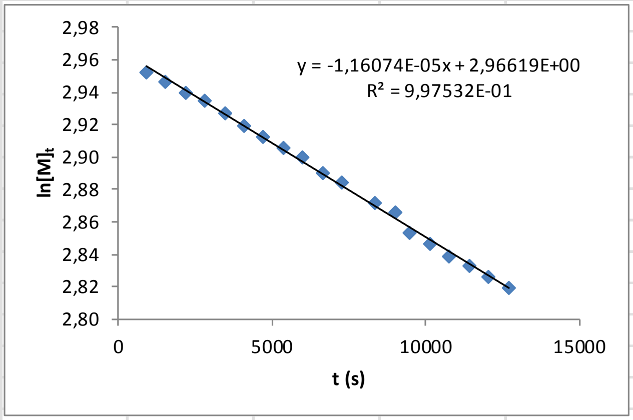

Figure S42. Kinetic experiment at 333 K: **3** (7.98 mg; 0.0086 mmol) and EtOH (0.283 mmol) in THF-d_8 (0.45 mL). $k_{obs} = 1.16 \pm 0.01 \times 10^{-5}$ s^{-1}.

$[M]_t$	t (s)	$\ln[M]_t$
18,503439	11730	2,91795663
18,387781	16758	2,911686383
18,231083	19032	2,903128002
17,954996	21315	2,8878684
17,781509	23599	2,878159079
17,600560	25875	2,867930699
17,029731	33891	2,83496068
16,934593	35036	2,829358424
16,781625	37321	2,820284554
16,723796	39603	2,816832627

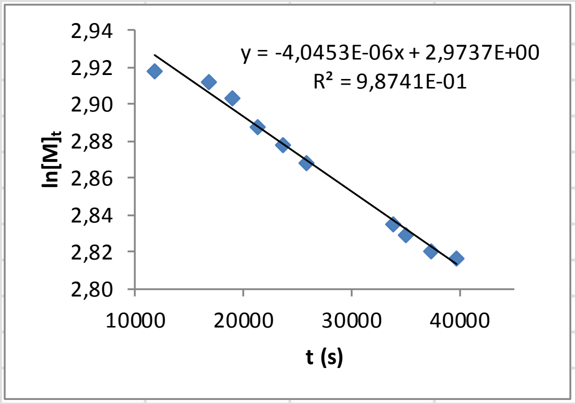

Figure S43. Kinetic experiment at 333 K: **3** (7.79 mg; 0.0084 mmol) and *n*PrOH (0.306 mmol) in THF-d_8 (0.45 mL). $k_{obs} = 4.0 \pm 0.2 \times 10^{-6}$ s^{-1}.

$[M]_t$	t (s)	$\ln[M]_t$
17,933360	0	2,886662693
17,633873	18473	2,869821676
17,569313	23075	2,866153817
17,476060	27678	2,860831933
17,397153	32300	2,856306573
17,318246	36912	2,851760641
17,239339	41529	2,847193949
17,112013	46140	2,839780707
17,025932	50590	2,83473762
16,914746	58102	2,828185763
16,773072	62711	2,819774748
16,591945	67325	2,808917345

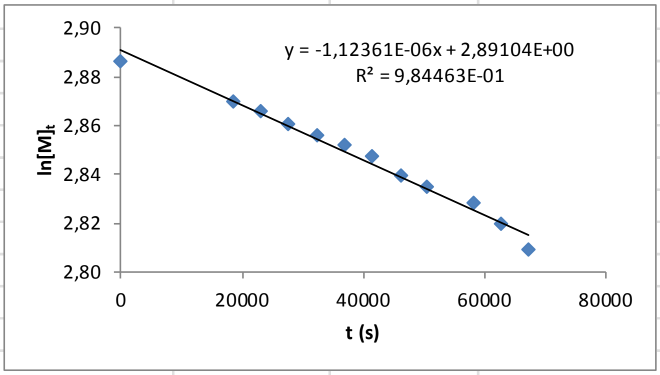

Figure S44. Kinetic experiment at 333 K: **3** (7.46 mg; 0.0081 mmol) and *i*PrOH (0.199 mmol) in THF-d_8 (0.45 mL). $k_{obs} = 1.12 \pm 0.04 \times 10^{-6}$ s^{-1}.

[M]$_t$	t (s)	ln[M]$_t$
17,332377	0	2,85257623
17,029060	633	2,834921295
16,963197	942	2,831046112
16,925066	1413	2,828795702
16,684146	1916	2,814458908
16,458825	2422	2,800861795
16,210972	2923	2,785688285
16,011649	3425	2,773316549
15,788062	3926	2,759254073
15,614738	4427	2,748215215
15,342620	4933	2,73063456
15,210894	5442	2,72201186
14,964774	5944	2,705699034
14,815715	6447	2,695688475

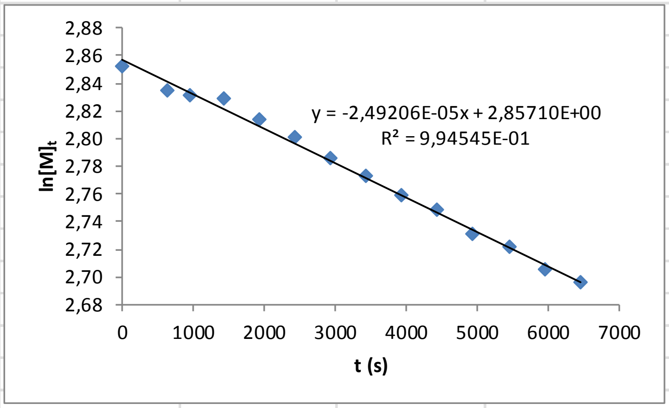

Figure S45. Kinetic experiment at 333 K: **3** (7.21 mg; 0.0078 mmol) and BnOH (0.206 mmol) in THF-d_8 (0.45 mL). $k_{obs} = 2.49 \pm 0.05 \times 10^{-5}$ s^{-1}.

8. Crystal structure determination of 1, 2, 4, 7, 8a and 8b

X-ray diffraction data were collected on a Smart APEX (S_{Rh}-**1**, R_{Ir}-**2,** S_{Ir}-**2·CH_2Cl_2**, *rac*-**2·CH_2Cl_2**) or APEX DUO (R_{Rh}-**1**, *rac*-**1, 4, 7**, **8a**, **8b**) Bruker diffractometers, using graphite-monochromated Mo κα radiation (λ = 0.71073 Å). Single crystals were mounted on a fiber or a MiTeGen support, coated with a protecting perfluoropolyether oil and cooled to 100(2) K (S_{Rh}-**1,** R_{Ir}-**2,** S_{Ir}-**2·CH_2Cl_2**, *rac*-**2·CH_2Cl_2**, **4**) or 150(2) K (R_{Rh}-**1,** *rac*-**1**) with an open-flow nitrogen gas. Data were collected using ω-scans and φ scans with narrow oscillation frame strategies. Diffracted intensities were integrated and corrected for absorption effects by using multi-scan method using SAINT[S2] and SADABS[S3] programs, included in APEX2 or APEX4 packages. Structures were solved by Patterson or direct methods with SHELXS[S4] and refined by full-matrix least squares on F^2 with SHELXL program[S5] included in Wingx program system.[S6] Hydrogen atoms have been observed in Fourier difference maps. Most of them have been included in the model in calculated positions and refined with a riding model. The NH hydrogen atoms have been included in observed positions (except for *rac*-**1** and *rac*-**2·CH_2Cl_2** structures), with geometrical restraints concerning N-H bond lengths (*rac*-**1**). Further details about specific problems encountered in data reduction or structure refinement (disorder or twinning) are described below.

Structural data for R_{Rh}-1: $C_{39}H_{45}ClF_6N_3PRhSb$; M_r = 960.86; red prism, 0.092 × 0.099 × 0.112 mm^3; orthorhombic $P2_12_12_1$; a = 11.9947(7) Å, b = 13.1691(7) Å, c = 25.9066(15) Å; V = 4092.2(4) Å^3, Z = 4, D_c = 1.560 g/cm^3; μ = 1.262 cm^{-1}; min. and max. absorption correction factors: 0.7378 and 0.9116, $2\theta_{max}$ = 59.240°; 43271 reflections measured, 10747 unique; R_{int} = 0.0731; number of data/restraint/parameters 10747/1/478; R_1 = 0.0493 [7471 ref. with $I > 2\sigma(I)$], w$R(F^2)$ = 0.1187 (all data); largest difference peak 1.075 e·Å^{-3}; Flack parameter: -0.019(15).

Structural data for S_{Rh}-1: $C_{39}H_{45}ClF_6N_3PRhSb$; M_r = 960.86; red prism, 0.290 × 0.320 × 0.340 mm^3; orthorhombic $P2_12_12_1$; a = 11.8200(6) Å, b = 13.0631(7) Å, c = 25.7405(14) Å; V = 3974.5(4) Å^3, Z = 4, D_c = 1.606 g/cm^3; μ = 1.262 cm^{-1}; min. and max. absorption correction factors: 0.5840 and 0.7468,

$2\theta_{max}$ = 56.374°; 35619 reflections measured, 9235 unique; R_{int} = 0.0365; number of data/restraint/parameters 9235/0/483; R_1 = 0.0394 [8570 ref. with $I > 2\sigma(I)$], w$R(F^2)$ = 0.0977 (all data); largest difference peak 2.343 e·Å^{-3}; Flack parameter: -0.021(11).

Structural data for *rac*-1: $C_{39}H_{45}ClF_6N_3PRhSb$; M_r = 960.86; orange needle, 0.085 × 0.091 × 0.313 mm^3; monoclinic *Cc*; *a* = 12.6541(10) Å, *b* = 24.6742(19) Å, *c* = 13.5627(10) Å; β = 97.7350(10) °; *V* = 4196.1(6) Å^3, *Z* = 4, D_c = 1.521 g/cm^3; μ = 1.169 cm^{-1}; min. and max. absorption correction factors: 0.6005 and 0.8648, $2\theta_{max}$ = 52.510°; 18121 reflections measured, 6700 unique; R_{int} = 0.0410; number of data/restraint/parameters 6700/3/477; R_1 = 0.0532 [5656 ref. with $I > 2\sigma(I)$], w$R(F^2)$ = 0.1436 (all data); largest difference peak 0.812 e·Å^{-3}; Flack parameter: -0.02(4).

Structural data for R_{Ir}-2: $C_{39}H_{45}ClF_6IrN_3PSb$; M_r = 1050.15; yellow prism, 0.080 × 0.110 × 0.180 mm^3; orthorhombic $P2_12_12_1$; *a* = 11.802(5) Å, *b* = 13.153(5) Å, *c* = 25.7260(12) Å; *V* = 3993(2) Å^3, *Z* = 4, D_c = 1.747 g/cm^3; μ = 4.172 cm^{-1}; min. and max. absorption correction factors: 0.5455 and 0.6876, $2\theta_{max}$ = 57.330°; 48763 reflections measured, 9669 unique; R_{int} = 0.0351; number of data/restraint/parameters 9669/0/476; R_1 = 0.0229 [9044 ref. with $I > 2\sigma(I)$], w$R(F^2)$ = 0.0456 (all data); largest difference peak 0.821 e·Å^{-3}; Flack parameter: -0.0160(19).

Structural data for S_{Ir}-2·CH_2Cl_2: $C_{39}H_{45}ClF_6IrN_3PSb·CH_2Cl_2$; M_r = 1135.07; yellow-orange prism, 0.085 × 0.150 × 0.174 mm^3; orthorhombic $P2_12_12_1$; *a* = 13.0839(6) Å, *b* = 13.1222(6) Å, *c* = 25.3504(11) Å; *V* = 4952.4(3) Å^3, *Z* = 4, D_c = 1.732 g/cm^3; μ = 3.954 cm^{-1}; min. and max. absorption correction factors: 0.5371 and 0.6789, $2\theta_{max}$ = 57.522°; 53697 reflections measured, 10564 unique; R_{int} = 0.0309; number of data/restraint/parameters 10564/0/511; R_1 = 0.0219 [10114 ref. with $I > 2\sigma(I)$], w$R(F^2)$ = 0.0520 (all data); largest difference peak 2.033 e·Å^{-3}; Flack parameter: -0.0147(17).

Structural data for *rac*-2·CH_2Cl_2: $C_{39}H_{45}ClF_6IrN_3PSb·2(CH_2Cl_2)$; M_r = 1220.0; yellow needle, 0.100 × 0.130 × 0.300 mm^3; monoclinic $P2_1$/c; *a* = 23.6453(12) Å, *b* = 13.5492(7) Å, *c* = 29.2459(15) Å; β =

100.2780(10) °; $V = 9219.3(8)$ Å^3, $Z = 8$, $D_c = 1.758$ g/cm^3; $\mu = 3.852$ cm^{-1}; min. and max. absorption correction factors: 0.4080 and 0.6119, $2\theta_{max} = 56.830°$; 168457 reflections measured, 22511 unique; $R_{int} = 0.0695$; number of data/restraint/parameters 22511/0/978; $R_1 = 0.0585$ [17676 ref. with $I > 2\sigma(I)$], w$R(F^2)$ = 0.1488 (all data); largest difference peak 3.146 e·Å^{-3}. Asymmetric unit of **rac-2·CH_2Cl_2** contains two iridium cation, two SbF_6 counterions and dichloromethane solvent. Interpretation of residual density peaks as solvent allows to locate three CH_2Cl_2 molecules. Model refinement is not stable for two of them, leading to unrealistic geometrical parameters. The solvent contribution to the diffracted intensities have been analysed with SQUEEZE program.[S7]

Structural data for 4·C_4H_8O: $C_{39}H_{44}F_6IrN_3PSb·C_4H_8O$; $M_r = 1085.79$; yellow prism, 0.195 × 0.265 × 0.360 mm^3; triclinic $P\bar{1}$; $a = 12.8032(6)$ Å, $b = 13.8442(6)$ Å, $c = 24.3349(11)$ Å; α =101.3316(8) °, β = 92.1614(7) °, ϒ = 90.7380(6) °; $V = 4225.4(3)$ Å^3, $Z = 4$, $D_c = 1.707$ g/cm^3; $\mu = 3.887$ cm^{-1}; min. and max. absorption correction factors: 0.5118 and 0.7457, $2\theta_{max} = 56.572°$; 51788 reflections measured, 35651 unique; $R_{int} = 0.0347$; number of data/restraint/parameters 35651/0/1032; $R_1 = 0.0295$ [32420 ref. with $I > 2\sigma(I)$], w$R(F^2)$ = 0.0880 (all data); largest difference peak 2.574 e·Å^{-3}. The sample cristallizes in transparent yellow prismatic crystals. In spite of their well-defined shape and transparency, all the tested crystals have been found to be twinned. Collected data have been refined as a 2-component twin. Both domains have been identified with CELL NOW[S8] and they have been found to be related by a 180 degree rotation around [1 0 0] axis. Difracction data have been corrected for absorption with TWINABS.[S8] Both domains contributions have been taken into account and single and composite reflections have been used in the structure refinement.

Structural data for 7: $C_{39}H_{44}F_6N_3PRhSb$; $M_r = 924.40$; yellow prism, 0.280 × 0.120 × 0.020 mm^3; monoclinic $P2_1/c$; $a = 19.910(4)$ Å, $b = 8.8909(17)$ Å, $c = 23.234(4)$ Å, $\beta = 112.681(3)°$; $V = 3794(13)$ Å^3, $Z = 4$, $D_c = 1.618$ g/cm^3; $\mu = 1.250$ cm^{-1}; min. and max. absorption correction factors: 0.7086 and 0.8826; $2\theta_{max} = 52.74°$; 26826 reflections measured, 7743 unique; $R_{int} = 0.0951$; number of

data/restraint/parameters 7743/2/477; R_1 = 0.0501 [$I > 2\sigma(I)$], w$R(F^2)$ = 0.1376 (all data); largest difference peak 1.876 e·Å^{-3}.

Structural data for 8a: $C_{39}H_{44}F_6IrN_3PSb$; M_r = 1013.69; yellow prism, 0.200 × 0.120 × 0.100 mm^3; monoclinic $P2_1/c$; a = 19.905(2) Å, b = 9.0017(9) Å, c = 23.224(2) Å, β = 112.5420(10)°; V = 3843.2(7) Å^3, Z = 4, D_c = 1.752 g/cm^3; μ = 4.265 cm^{-1}; min. and max. absorption correction factors: 0.4859 and 0.5481; $2\theta_{max}$ = 56.63°; 51322 reflections measured, 9545 unique; R_{int} = 0.0366; number of data/restraint/parameters 9545/2/479; R_1 = 0.0318 [$I > 2\sigma(I)$], w$R(F^2)$ = 0.0831 (all data); largest difference peak 2.988 e·Å^{-3}.

Structural data for 8b: $C_{39}H_{44}F_6IrN_3PSb$; M_r = 1013.69; yellow prism, 0.200 × 0.160 × 0.040 mm^3; monoclinic $P2_1/c$; a = 15.2688(13) Å, b = 18.2608(16) Å, c = 14.9258(13) Å, β = 116.1320(10)°; V = 3736.2(6) Å^3, Z = 4, D_c = 1.802 g/cm^3; μ = 4.387 cm^{-1}; min. and max. absorption correction factors: 0.4470 and 0.6035; $2\theta_{max}$ = 56.58°; 39673 reflections measured, 9263 unique; R_{int} = 0.0525; number of data/restraint/parameters 9263/2/475; R_1 = 0.0280 [$I > 2\sigma(I)$], w$R(F^2)$ = 0.0642 (all data); largest difference peak 1.322 e·Å^{-3}.

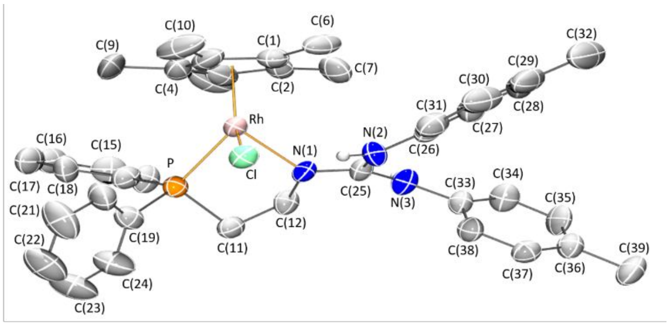

Figure S46. View of the cation of the R_{Rh} enantiomer of the rhodium complex [Cp*RhCl(κ^2*N,P*-**H$_2$L**)][SbF$_6$] (**1**) in *rac*-**1**.

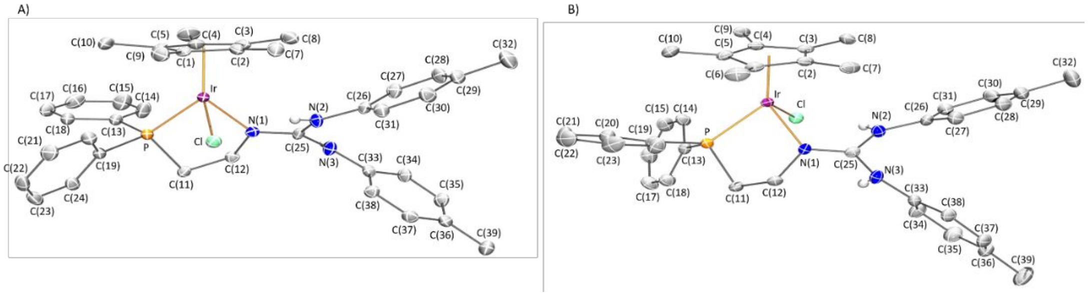

Figure S47. View of the cations of the R_{Ir} (A) and S_{Ir} (B) enantiomers of the iridium complex [Cp*IrCl(κ[2]*N,P*-**H_2L**)][SbF_6] (**2**) in S_{Ir}-**2** and R_{Ir}-**2·CH_2Cl_2** crystals.

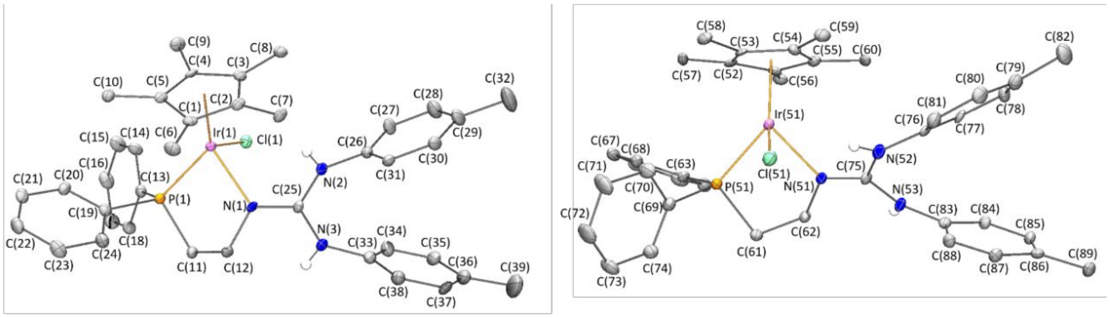

Figure S48. View of both independent cations of the asymmetric unit of the iridium complex [Cp*IrCl(κ[2]*N,P*-**H_2L**)][SbF_6] (**1**) in *rac* -**2**. They correspond to $S_{Ir(1)}$ $_{a}$nd $R_{Ir(51)}$. As the crystal is found to be centrosymmetric, the corresponding $R_{Ir(1)}$ and $S_{Ir(51)}$ are also present in the unit cell.

Table S1. Selected Bonds Lengths (Å) and Angles (º) for complex R_{Rh}-**1**, *rac*-**1**, R_{Ir}-**2**, ***S_{Ir}*-2·CH_2Cl_2** and ***rac*-2·CH_2Cl_2**.

	R_{Rh}-**1**	*rac*-**1**	R_{Ir}-**2**	***S_{Ir}*-2 CH_2Cl_2**	***rac*-2·CH_2Cl_2**	
					A[c]	***B***[c]
M-Cl	2.4264(18)	2.424(2)	2.4254(12)	2.4243(10)	2.4254(18)	2.428(2)
M-P	2.2990(19)	2.322(3)	2.2831(13)	2.2919(12)	2.2848(18)	2.2831(19)
M-N(1)	2.133(6)	2.130(8)	2.126(4)	2.115(4)	2.124(5)	2.132(6)
M-Ct[a]	1.8308(1)	1.8370(1)	1.834(2)	1.830(5)	1.836(3)	1.838(3)
N(1)-C(25)	1.303(8)	1.318(13)	1.317(6)	1.320(6)	1.325(9)	1.315(9)
N(2)-C(25)	1.341(9)	1.353(12)	1.344(6)	1.351(6)	1.342(9)	1.350(10)
N(3)-C(25)	1.370(8)	1.338(13)	1.366(6)	1.366(6)	1.361(9)	1.372(9)
Cl-M-P	90.40(7)	92.25(9)	89.60(5)	87.44(4)	89.64(6)	88.70(7)
Cl-M-N(1)	85.40(17)	90.9(2)	83.69(12)	86.64(11)	86.41(18)	85.69(18)
Cl-M-Ct[a]	121.9(2)	121.41(1)	122.28(8)	122.67(16)	122.09(13)	123.16(13)
P-M-N(1)	83.12(15)	81.3(2)	82.97(11)	82.31(10)	82.27(17)	82.52(17)
P-M-Ct[a]	130.6(2)	132.38(1)	131.52(8)	133.79(17)	132.87(12)	132.94(13)
N(1)-M-Ct[a]	131.0(2)	125.92(1)	131.39(13)	128.43(19)	128.86(18)	128.66(18)
M-N(1)-C(12)	117.6(4)	115.6(6)	118.2(3)	117.3(3)	117.0(4)	116.5(4)
M-N(1)-C(25)	122.9(5)	124.3(7)	123.2(3)	124.3(3)	124.3(5)	123.5(5)
C(12)-N(1)-C(25)	119.2(6)	119.7(9)	118.4(4)	118.3(4)	118.7(6)	120.0(6)
Σ°N(1)[b]	359.7(9)	359.6(13)	359.8(6)	359.9(6)	360.0(9)	360.0(9)

M =Rh (**1**) and Ir (**2**); [a] Ct represents the centroid of the η^5-C_5Me_5 ligand. [b] Σ°N(1) is the sum of bond angles around the N(1) atom. [c] Asymmetric unit of *rac*-**2**·CH_2Cl_2 contains two independent molecules.

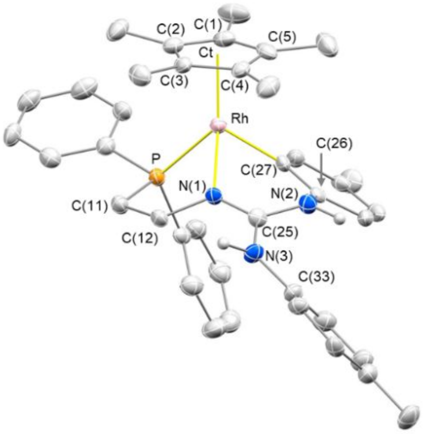

Figure S49. ORTEP plot of the cation of compound **7**. Thermal ellipsoids are at 50 % probability and most hydrogen atoms have been omitted for clarity.

Table S2. Selected Bonds Lengths (Å) and Angles (º) for **7**.

Rh-Ct*	1.8510(6)	Ct-Rh-C(27)	125.60(17)
Rh-P	2.2664(18)	P-Rh-N(1)	77.27(15)
Rh-N(1)	2.071(5)	P-Rh-C(27)	91.59(18)
Rh-C(27)	2.053(6)	N(1)-Rh-C(27)	85.3(2)
N(1)-C(25)	1.304(8)	Rh-N(1)-C(12)	117.8(4)
N(2)-C(25)	1.362(8)	Rh-N(1)-C(25)	121.6(4)
N(3)-C(25)	1.363(8)	C(12)-N(1)-C(25)	120.2(5)
Ct-Rh-P	133.55(5)	Σ°N(1)[b]	359.6(7)
Ct-Rh-N(1)	126.64(15)	N(1)-C(25)-N(3)-C(33)	157.3(6)

[a] Ct, centroid of the C(1)–C(2)−C(3)−C(4)−C(5) ring. [b]Σ°N(1) is the sum of bond angles around the N(1) atom

9. DFT calculations

Molecular structure optimizations and frequency calculations were carried out with the Gaussian09 program (revision D.01)[S9] using the method wB97XD.[S10] The def2-SVP[S11] basis and pseudo potential were used for all atoms and the "ultrafine" grid was employed in all calculations. Stationary points were characterized by vibrational analysis. The structures were optimized in dichloromethane (298 K, 1 atm) using the SMD method.[S12] In order to improve the accuracy of the calculated energies, single point energy calculations were carried out on the optimized structures of intermediates and transition states using the method wB97XD,[S10] the def2-TZVP[S11] basis and pseudo potentials, where appropriate, and the SMD model[S12] for the solvent (dichloromethane). Finally, a correction of +1.89 $kcal \cdot mol^{-1}$ to Gibbs free energy was also applied for the change of the standard state from gas phase (1 atm) to solution (1 M) at 298 K.[S13]

10. Energies and coordinates of calculated structures

Table S3. Energies (Hartree) of the calculated structures (298 K, 1 atm, if not stated otherwise).

	E_dz[a]	G_dz[b]	E_tz[c]	G_tz[d]
**3**	-2127.042073	-2126.372546	-2128.944564	-2128.275037
**3 (333 K)**	-2127.042073	-2126.387021	-2128.944564	-2128.289511
**3·H_2O**	-2203.397308	-2202.708317	-2205.392437	-2204.703446
**3·MeOH**	-2242.654835	-2241.940354	-2244.689829	-2243.975348
**4**	-2120.887653	-2120.218755	-2122.779324	-2122.110426
**4 (333 K)**	-2120.887653	-2120.233334	-2122.779324	-2122.125005
**4·H_2O**	-2197.243419	-2196.555708	-2199.227582	-2198.539871
**4·MeOH**	-2236.501024	-2235.785664	-2238.525102	-2237.809742
**5**	-2128.255008	-2127.566095	-2130.159219	-2129.470306
**5·CH_2O**	-2242.646051	-2241.929570	-2244.682391	-2243.965910
**6**	-2122.104235	-2121.415017	-2123.998654	-2123.309436
**6·CH_2O**	-2236.496154	-2235.780668	-2238.522589	-2237.807103
**7a**	-2127.053379	-2126.382775	-2128.952763	-2128.282159
**7b**	-2127.051181	-2126.379968	-2128.951076	-2128.279863
**8a**	-2120.901525	-2120.229637	-2122.790127	-2122.118239
**8b**	-2120.899639	-2120.228952	-2122.788787	-2122.118100
**CH_2O**	-114.379369	-114.374013	-114.515663	-114.510307
**H_2O**	-76.343172	-76.339320	-76.444355	-76.440503
**I^{Ir}**	-2120.870043	-2120.202708	-2122.763070	-2122.095735
**I^{Ir}·H_2O**	-2197.237030	-2196.549030	-2199.222197	-2198.534197
**I^{Ir}·MeOH**	-2236.497338	-2235.779488	-2238.521051	-2237.803201
**I^{Rh}**	-2127.019603	-2126.352571	-2128.924417	-2128.257385
**I^{Rh}·H_2O**	-2203.386675	-2202.696522	-2205.382615	-2204.692462
**I^{Rh}·MeOH**	-2242.646266	-2241.928865	-2244.681944	-2243.964543
**II^{Ir}**	-2197.254252	-2196.559150	-2199.236047	-2198.540945
**II^{Rh}**	-2203.410577	-2202.715293	-2205.403349	-2204.708065
**III^{Ir}**	-2197.231891	-2196.538604	-2199.212478	-2198.519191
**III^{Rh}**	-2203.393406	-2202.703106	-2205.388058	-2204.697758
**IV^{Ir}**	-2236.510215	-2235.787196	-2238.530839	-2237.807820
**IV^{Rh}**	-2242.666632	-2241.944188	-2244.698090	-2243.975646
**MeOH**	-115.600166	-115.571326	-115.739061	-115.710221
**MeOH (333 K)**	-115.600166	-115.574510	-115.739061	-115.713405
**TS_II^{Ir}-III^{Ir}**	-2197.212791	-2196.521748	-2199.191234	-2198.500191
**TS_I^{Ir}·MeOH-6·CH_2O**	-2236.474834	-2235.756630	-2238.496819	-2237.778615
**TS_I^{Ir}·MeOH-6·CH_2O (333 K)**	-2236.474834	-2235.771361	-2238.496819	-2237.793346
**TS_II^{Rh}-III^{Rh}**	-2203.376985	-2202.685547	-2205.367545	-2204.676107
**TS_I^{Rh}·MeOH-5·CH_2O**	-2242.630320	-2241.912518	-2244.663799	-2243.945997
**TS_I^{Rh}·MeOH-5·CH_2O (333 K)**	-2242.630320	-2241.927273	-2244.663799	-2243.960752
**TS_IV^{Ir}-V^{Ir}**	-2236.471174	-2235.753423	-2238.489089	-2237.771338
**TS_IV^{Rh}-V^{Rh}**	-2242.635224	-2241.917995	-2244.665132	-2243.947903
**V^{Ir}**	-2236.488816	-2235.772776	-2238.508975	-2237.792935
**V^{Rh}**	-2242.650968	-2241.934266	-2244.684243	-2243.967541

[a] E_dz: energy at the wB97XB/def2svp level (SMD); [b] G_dz: Gibbs free energy at the wB97XB/def2svp level (SMD); [c] E_tz: energy at the wB97XB/def2tzvp level (SMD); [d] G_tz = Gdz-E_dz+E_tz (SMD).

3

Rh	0.63434200	0.06903300	-1.03136000
P	1.51155600	-0.77403300	0.97355200
N	-0.92348700	-1.23362900	-0.45597600
N	-1.04785000	0.92422500	-0.04925500
N	-2.81396700	-0.40161000	0.79049700
C	1.99694000	-0.57464100	-2.59351000
C	0.70485500	-0.34588500	-3.18819900
C	0.34941000	1.01798400	-2.97956100
C	1.44968500	1.66200200	-2.28011800
C	2.46993900	0.69248200	-2.07364100
C	2.79924400	-1.82855400	-2.72013100
H	2.17729100	-2.72376500	-2.58194500
H	3.61429400	-1.86337900	-1.98695200
H	3.24647600	-1.88556400	-3.72586200
C	-0.11623000	-1.37827000	-3.88486600
H	-1.18717700	-1.14087700	-3.84163500
H	0.03460000	-2.37296700	-3.44421000
H	0.17739100	-1.43248800	-4.94553400
C	-0.89129400	1.70009700	-3.45438800
H	-0.73958600	2.10066600	-4.46959100
H	-1.16142400	2.53995100	-2.80019900
H	-1.74277300	1.00706500	-3.48956000
C	1.51734700	3.11270500	-1.93360800
H	2.23784100	3.30229200	-1.12635400
H	0.53764400	3.49657500	-1.61758400
H	1.83589500	3.69383800	-2.81412100
C	3.83351600	0.95646200	-1.52643800
H	4.49744600	1.26898600	-2.34856300
H	4.27021000	0.05974100	-1.07041100
H	3.82655300	1.76133200	-0.77929900
C	1.80534700	0.42566000	2.30948300
C	2.15996100	1.73500100	1.96684600
H	2.21342300	2.02162200	0.91308800
C	2.43680000	2.67158800	2.96196900
H	2.71127900	3.69233800	2.68693900
C	2.35236500	2.30435700	4.30439800
H	2.56121400	3.03844500	5.08585000
C	2.00125900	0.99855900	4.65147700
H	1.93815500	0.70973600	5.70290100
C	1.73484300	0.05756900	3.65895000
H	1.47490200	-0.96559700	3.94175300
C	3.06527000	-1.73302800	0.89085000
C	4.29103100	-1.15190700	1.23883400
H	4.33075900	-0.12442100	1.60668600
C	5.47369500	-1.88119200	1.12213800
H	6.42314500	-1.41634700	1.39677200
C	5.44393200	-3.19792400	0.66454400
H	6.37110700	-3.76871000	0.57710400
C	4.22521500	-3.78593200	0.32540200
H	4.19317900	-4.81854800	-0.02877700
C	3.04255400	-3.05779000	0.43380000
H	2.10068900	-3.53342900	0.15301600
C	0.22999700	-1.97125900	1.56546700
H	0.68678600	-2.81877400	2.09631200
H	-0.40309300	-1.42870400	2.28524200
C	-0.61522000	-2.39978400	0.35934000
H	-0.06849900	-3.11558100	-0.27144200
H	-1.52362300	-2.91628200	0.70890500

C	-1.65465600	-0.23898000	0.09312200
C	-1.62889100	2.19406700	0.07034200
C	-0.92177700	3.22221400	0.70681100
H	0.05755500	3.00377800	1.13375700
C	-1.45881500	4.50264000	0.79496200
H	-0.88585600	5.28724600	1.29737400
C	-2.71818600	4.80540400	0.25968400
C	-3.41608400	3.77314100	-0.37825500
H	-4.39593800	3.97812500	-0.81845800
C	-2.88458700	2.48931300	-0.48192400
H	-3.44419600	1.71132200	-1.00584000
C	-3.30328900	6.18601400	0.39122900
H	-4.12832200	6.34387700	-0.31811600
H	-2.54479100	6.96334100	0.21428600
H	-3.70345400	6.34842500	1.40572800
C	-3.76553300	-1.43610700	0.71429200
C	-4.70723900	-1.53494300	1.74595900
H	-4.66462500	-0.83158700	2.58159800
C	-5.69500700	-2.51405000	1.70878200
H	-6.42079500	-2.56420500	2.52508100
C	-5.77409900	-3.43252200	0.65488400
C	-4.82746500	-3.31858100	-0.36983000
H	-4.86320000	-4.01285400	-1.21399700
C	-3.83714900	-2.33893500	-0.35354000
H	-3.11902000	-2.28156100	-1.17197200
C	-6.81547600	-4.51950600	0.64101000
H	-6.43933500	-5.43114100	1.13440900
H	-7.09565800	-4.79544900	-0.38618700
H	-7.72617900	-4.21096900	1.17484800
H	-3.09512100	0.40077400	1.34664600

3·H_2O

Rh	0.53939100	-0.15348500	-1.04774400
P	1.29456100	-1.05144500	0.99529800
N	-1.17914800	-1.21178100	-0.44992300
N	-0.98418700	0.94116000	-0.07169500
N	-2.91514300	-0.09669600	0.80549200
C	2.02743500	-0.87434600	-2.47517700
C	0.73816300	-0.87393100	-3.09900200
C	0.23802600	0.47033900	-3.10415700
C	1.25414800	1.31401500	-2.51278600
C	2.34840700	0.49452300	-2.11411900
C	2.97228300	-2.02926500	-2.42853300
H	2.44607300	-2.98176300	-2.28405700
H	3.71451300	-1.92064700	-1.62798100
H	3.51741300	-2.08930800	-3.38471500
C	0.03143000	-2.06811500	-3.64709500
H	-1.05561800	-1.98568400	-3.51385800
H	0.37063200	-2.99230100	-3.16071900
H	0.23538400	-2.15991400	-4.72603400
C	-1.04352100	0.93808100	-3.71303800
H	-0.89877100	1.17566300	-4.77921700
H	-1.41356500	1.84464600	-3.21494100
H	-1.82405900	0.16779400	-3.64375800
C	1.16577500	2.79808000	-2.38831500
H	1.80986900	3.17494300	-1.58465300
H	0.13624300	3.12783500	-2.19302500
H	1.49121600	3.26253200	-3.33342900

C	3.65612600	0.95937600	-1.55905100
H	4.37764200	1.10232800	-2.38024200
H	4.08519200	0.21878800	-0.87021800
H	3.55571700	1.91476900	-1.02591500
C	1.71734300	0.09443900	2.34628100
C	2.28604900	1.33092600	2.02347800
H	2.42419900	1.61707900	0.97854200
C	2.67926100	2.21074400	3.03168800
H	3.12315300	3.16917400	2.75522500
C	2.49629900	1.86108500	4.36872900
H	2.79835900	2.55011100	5.16088200
C	1.92938600	0.62807600	4.69707100
H	1.78865900	0.35135900	5.74430800
C	1.54654200	-0.25753400	3.69160000
H	1.11869100	-1.22594400	3.96255300
C	2.72899800	-2.17818400	0.92005600
C	4.02148600	-1.69915200	1.16834400
H	4.17698100	-0.66496600	1.48360400
C	5.12281100	-2.54126900	1.02124800
H	6.12614500	-2.15706000	1.21776500
C	4.94435700	-3.86862900	0.63202600
H	5.80817300	-4.52763400	0.51990500
C	3.65874000	-4.35373600	0.39352800
H	3.51136800	-5.39349300	0.09328600
C	2.55627000	-3.51279800	0.53153400
H	1.55900800	-3.90719800	0.32402200
C	-0.13004500	-2.08088200	1.57941400
H	0.21153700	-2.97814700	2.11525500
H	-0.69264200	-1.46240200	2.29615200
C	-1.02357800	-2.40196000	0.37466900
H	-0.57208600	-3.18538100	-0.25110000
H	-1.99215300	-2.79396900	0.72531100
C	-1.75248600	-0.11660500	0.09820300
C	-1.36635600	2.28543500	0.02773600
C	-0.57220100	3.18519100	0.74777700
H	0.30836300	2.80649000	1.26938800
C	-0.90673400	4.53577100	0.80344600
H	-0.27317200	5.21933800	1.37635000
C	-2.04180700	5.03407600	0.15037600
C	-2.83297400	4.12410600	-0.56263800
H	-3.72357800	4.48180000	-1.08690800
C	-2.50456700	2.77218400	-0.63170000
H	-3.12918200	2.08528700	-1.20758700
C	-2.41860500	6.48853300	0.23965200
H	-2.92256900	6.83053100	-0.67611000
H	-1.53700900	7.12413500	0.40670400
H	-3.11242400	6.66352200	1.07867500
C	-3.99174800	-1.00343800	0.75310500
C	-4.87827800	-1.04052800	1.83445800
H	-4.70269200	-0.39205300	2.69660800
C	-5.97907500	-1.89258800	1.81283100
H	-6.66148500	-1.89822000	2.66710900
C	-6.22413500	-2.74082000	0.72699800
C	-5.32744600	-2.69010500	-0.34806300
H	-5.49583400	-3.33093300	-1.21813400
C	-4.22924200	-1.83454600	-0.34856100
H	-3.55198600	-1.81221000	-1.20367200
C	-7.39322700	-3.68898300	0.72002000
H	-7.08298600	-4.70092300	1.02941500

H	-7.83299500	-3.77742600	-0.28454500
H	-8.18177100	-3.36038000	1.41212000
H	-3.07462700	0.74246400	1.35555800
O	3.04523600	3.97240600	0.31194700
H	3.38927300	4.64075900	-0.29286800
H	2.12399000	4.23168900	0.43906100

3·MeOH

Rh	0.42595800	-0.36215400	-1.01710500
P	1.11395300	-1.31635800	1.02069800
N	-1.39359000	-1.19684100	-0.36671100
N	-0.93037600	0.92007400	-0.02497400
N	-2.97036200	0.14619500	0.87806200
C	1.83837500	-1.22937300	-2.45186200
C	0.54052500	-1.15358100	-3.05097200
C	0.13799700	0.22486700	-3.08677900
C	1.22076400	1.00668300	-2.53416100
C	2.25919000	0.12048400	-2.12508600
C	2.70268100	-2.44475600	-2.39221300
H	2.11563700	-3.35840000	-2.23418700
H	3.45536300	-2.37598800	-1.59708800
H	3.23730000	-2.55194300	-3.35022200
C	-0.26135800	-2.30515900	-3.55880200
H	-1.33572500	-2.14588700	-3.39492600
H	0.02823600	-3.24379300	-3.06792600
H	-0.09788700	-2.42622100	-4.64172100
C	-1.11981100	0.76336200	-3.68685000
H	-0.99901000	0.90761800	-4.77249100
H	-1.38459200	1.73463500	-3.24795000
H	-1.96350100	0.07675700	-3.53143600
C	1.24494200	2.49414000	-2.42818400
H	1.83288800	2.81921800	-1.56114900
H	0.23314100	2.91142900	-2.33887200
H	1.70662200	2.91829900	-3.33452300
C	3.60062500	0.50873600	-1.59130800
H	4.32831800	0.57413400	-2.41679900
H	3.97818000	-0.23506100	-0.87587800
H	3.56658700	1.48560600	-1.08948000
C	1.69242000	-0.20789600	2.34326400
C	2.34296500	0.97886200	1.99192900
H	2.45670100	1.25756600	0.94189700
C	2.84401400	1.82646500	2.97985400
H	3.34035800	2.75090700	2.67836100
C	2.69196300	1.49242200	4.32449000
H	3.07852100	2.15714100	5.10058800
C	2.04465800	0.30745800	4.68128300
H	1.92654700	0.04333700	5.73456600
C	1.55073100	-0.54487900	3.69601300
H	1.05616600	-1.47515600	3.98699100
C	2.40842100	-2.59940200	0.93362100
C	3.74934100	-2.27550800	1.17626400
H	4.02587600	-1.26757000	1.49456200
C	4.74355500	-3.24000200	1.01789700
H	5.78616400	-2.97723100	1.21003600
C	4.40844900	-4.53504500	0.62294300
H	5.18854000	-5.29007100	0.50243600
C	3.07357100	-4.86494500	0.38908800
H	2.80399000	-5.87840900	0.08381200

C	2.07791100	-3.90165600	0.53844000
H	1.03983200	-4.17323100	0.33370600
C	-0.40837300	-2.16620100	1.65123300
H	-0.15972700	-3.09604500	2.18285200
H	-0.87054400	-1.48344000	2.38140100
C	-1.37038200	-2.38421000	0.47566400
H	-1.04367600	-3.23119700	-0.14531600
H	-2.37195700	-2.63919400	0.86004700
C	-1.82103400	-0.03143700	0.16874800
C	-1.14186900	2.30365700	0.06414800
C	-0.28572300	3.09169200	0.84266000
H	0.50027500	2.60200800	1.41928000
C	-0.43971200	4.47454300	0.88477300
H	0.23922000	5.06918600	1.50414500
C	-1.45433400	5.11801500	0.16202000
C	-2.31321900	4.31930700	-0.60234100
H	-3.11469100	4.79036600	-1.17814100
C	-2.16257300	2.93472400	-0.65918800
H	-2.83371800	2.33714000	-1.27989900
C	-1.63470700	6.61018100	0.23923800
H	-2.16777100	6.99739000	-0.64088700
H	-0.66774900	7.12928600	0.31358400
H	-2.22260100	6.88779000	1.12980200
C	-4.17643600	-0.57554800	0.78690700
C	-5.13433600	-0.38778600	1.79090700
H	-4.91739600	0.28673800	2.62334900
C	-6.35763400	-1.04749300	1.72946400
H	-7.09004100	-0.87967000	2.52386400
C	-6.66539200	-1.92056900	0.67844300
C	-5.69845300	-2.09422800	-0.31826800
H	-5.90931800	-2.75919600	-1.16047900
C	-4.47255700	-1.43355800	-0.27856400
H	-3.74470700	-1.58239300	-1.07707000
C	-7.97400500	-2.66333900	0.63863000
H	-7.91330900	-3.60125700	1.21535400
H	-8.25381000	-2.92947700	-0.39101600
H	-8.78921200	-2.06613800	1.07291000
H	-3.02397900	1.01985700	1.39441700
O	3.09113700	3.61711000	0.25240900
H	2.20007000	3.97337800	0.34685400
C	3.86879700	4.53509000	-0.48083800
H	4.88025800	4.11605900	-0.57811800
H	3.47126400	4.70488000	-1.49765900
H	3.95275000	5.51098200	0.02877400

4

P	1.46920900	-0.79189100	1.09198500
N	-0.97747900	-1.25832100	-0.29810500
N	-1.09090600	0.88900200	0.09844100
N	-2.87412700	-0.42023600	0.94100400
C	1.92530600	-0.61072700	-2.53524500
C	0.62023600	-0.35686000	-3.09952800
C	0.29223500	1.01560900	-2.88873800
C	1.41501100	1.63814300	-2.20009200
C	2.42396800	0.64687800	-2.01622400
C	2.71012500	-1.87277700	-2.69572500
H	2.07496100	-2.76131800	-2.57586800
H	3.52388800	-1.93646200	-1.96261300
H	3.15701800	-1.91337900	-3.70225800

C	-0.23357200	-1.37768700	-3.77489700
H	-1.29829700	-1.11709300	-3.71457000
H	-0.09570800	-2.37052100	-3.32542400
H	0.04059900	-1.44839800	-4.83955700
C	-0.94241900	1.72016000	-3.34923200
H	-0.79779000	2.11889500	-4.36605700
H	-1.18949700	2.56339400	-2.69021000
H	-1.80667600	1.04246900	-3.37187000
C	1.52517800	3.09064300	-1.86861100
H	2.24581700	3.26528200	-1.05790200
H	0.55576700	3.50589800	-1.56091000
H	1.86726400	3.65360800	-2.75200100
C	3.79878600	0.88770300	-1.48526500
H	4.45690800	1.20137500	-2.31137100
H	4.22791500	-0.01966300	-1.04322400
H	3.80870100	1.68325500	-0.72814600
C	1.78059900	0.40366900	2.42884900
C	2.13751300	1.71325400	2.08923700
H	2.18268200	2.00407200	1.03621200
C	2.42721700	2.64406900	3.08581500
H	2.70275900	3.66504000	2.81269700
C	2.35521300	2.27070200	4.42732700
H	2.57434700	3.00033800	5.21014400
C	2.00384000	0.96431100	4.77146200
H	1.95082400	0.67041300	5.82201900
C	1.72376500	0.02920100	3.77702100
H	1.46339500	-0.99441900	4.05772400
C	3.02424300	-1.74417100	0.97512500
C	4.25229800	-1.17000700	1.32388600
H	4.29556500	-0.14923500	1.70950000
C	5.43345100	-1.89762300	1.18267200
H	6.38580300	-1.43857200	1.45696400
C	5.39837600	-3.20541800	0.70077600
H	6.32434500	-3.77491300	0.59421100
C	4.17636300	-3.78654400	0.36103100
H	4.14072900	-4.81226500	-0.01229400
C	2.99541300	-3.05977900	0.49337000
H	2.04994700	-3.52848700	0.21196000
C	0.20268400	-1.99487300	1.70535700
H	0.67389200	-2.84216700	2.22332300
H	-0.41802900	-1.45763100	2.43932400
C	-0.66725300	-2.42512800	0.51669100
H	-0.13844900	-3.14885800	-0.12023400
H	-1.57614200	-2.92694800	0.88471100
C	-1.71381200	-0.26708700	0.24740000
C	-1.65605300	2.17182300	0.17804200
C	-0.92380000	3.21236700	0.76293100
H	0.05783100	2.99453700	1.18496100
C	-1.43856700	4.50360800	0.80252000
H	-0.84669000	5.29961600	1.26335300
C	-2.69966900	4.80401000	0.26834800
C	-3.42388000	3.75714800	-0.31235300
H	-4.40628500	3.95911900	-0.74798700
C	-2.91462500	2.46087800	-0.36702500
H	-3.49409600	1.67181200	-0.85101000
C	-3.24801500	6.20467500	0.32684000
H	-4.18103200	6.29555800	-0.24713800
H	-2.52744000	6.93333200	-0.07571300
H	-3.46279700	6.50298300	1.36569000

C	-3.86068300	-1.41754600	0.82149800
C	-4.88996000	-1.42894800	1.77229400
H	-4.88324800	-0.69362200	2.58133400
C	-5.92033200	-2.35826100	1.68577700
H	-6.71379000	-2.33947100	2.43807700
C	-5.96196100	-3.31040000	0.65882500
C	-4.92736400	-3.28581100	-0.28191600
H	-4.93013700	-4.00873000	-1.10239400
C	-3.88973100	-2.35718600	-0.21520800
H	-3.10829400	-2.36389600	-0.97550700
C	-7.06518300	-4.33206500	0.58984300
H	-6.86505300	-5.17759200	1.26869400
H	-7.17072800	-4.74214000	-0.42490400
H	-8.03180800	-3.89924900	0.88738600
H	-3.15142700	0.38821500	1.49044800
Ir	0.58989900	0.04638100	-0.92371400

4·H_2O

P	1.26979100	-1.00367300	1.12786600
N	-1.18153200	-1.26338600	-0.33110200
N	-1.05160800	0.89107000	-0.00654000
N	-2.98769500	-0.15687000	0.84585300
C	2.14980200	-0.79869800	-2.32296500
C	0.88118900	-0.95722600	-2.97092200
C	0.25295900	0.33641800	-3.07529000
C	1.16332000	1.30046900	-2.50215700
C	2.31740300	0.61216200	-2.01529700
C	3.21113000	-1.84261700	-2.21248200
H	2.78661100	-2.84702800	-2.08992000
H	3.88911800	-1.65121600	-1.37171000
H	3.81266400	-1.84145700	-3.13604200
C	0.30979200	-2.23867800	-3.48048800
H	-0.78273400	-2.25843900	-3.36854700
H	0.72764500	-3.10286600	-2.94745900
H	0.54311500	-2.35459200	-4.55094000
C	-1.03557000	0.63502000	-3.77248700
H	-0.87577400	0.72710000	-4.85868700
H	-1.47201900	1.57786800	-3.41648000
H	-1.77206400	-0.16403600	-3.60758900
C	0.93251700	2.77392100	-2.45319800
H	1.48322900	3.23823900	-1.62680300
H	-0.13354200	3.01254300	-2.33787800
H	1.27936100	3.22897700	-3.39486300
C	3.55044000	1.23493100	-1.44141300
H	4.28746900	1.42003100	-2.23986000
H	4.02147900	0.57288700	-0.70155700
H	3.32831900	2.19368800	-0.95244700
C	1.64991700	0.18155500	2.45698600
C	2.12473900	1.45063000	2.11171700
H	2.21615200	1.73546000	1.06118200
C	2.47311200	2.36686300	3.10392400
H	2.83140800	3.35507000	2.80878200
C	2.34464700	2.01760400	4.44723600
H	2.61168800	2.73446300	5.22718900
C	1.87437800	0.75025700	4.79810400
H	1.77572800	0.47477400	5.85045100
C	1.53250500	-0.16901300	3.80848500
H	1.17656800	-1.16179800	4.09549300

C	2.73521000	-2.09003000	1.08149900
C	4.00575700	-1.58907400	1.38926500
H	4.12738700	-0.55684300	1.72518300
C	5.12883200	-2.40758800	1.27407500
H	6.11573700	-2.00741700	1.51650400
C	4.99216500	-3.73166000	0.85845400
H	5.87269400	-4.37214500	0.77202300
C	3.72719000	-4.23766100	0.55904000
H	3.61353200	-5.27482100	0.23611100
C	2.60381600	-3.42042300	0.66425200
H	1.62347800	-3.82878500	0.40768100
C	-0.12670600	-2.06196100	1.72641900
H	0.24247100	-2.94183600	2.27234900
H	-0.70712500	-1.45163100	2.43546100
C	-1.01086200	-2.42880300	0.52712500
H	-0.54699800	-3.22305800	-0.07527200
H	-1.97483600	-2.82284300	0.88626500
C	-1.80788300	-0.17527000	0.17137400
C	-1.44834600	2.23749500	0.06639100
C	-0.77036500	3.11785000	0.91736400
H	0.01529600	2.72330000	1.56366300
C	-1.10221200	4.46973700	0.94436300
H	-0.56177000	5.14016100	1.61974500
C	-2.12417500	4.98478500	0.13392700
C	-2.80928300	4.09065200	-0.69685300
H	-3.61386200	4.46160200	-1.33773900
C	-2.47955400	2.73671700	-0.73782400
H	-3.01743400	2.06345000	-1.40870600
C	-2.49325100	6.44251400	0.18763200
H	-3.00994000	6.76082200	-0.72897000
H	-1.60514600	7.07712300	0.32300000
H	-3.16913800	6.64435500	1.03500300
C	-4.06422200	-1.06285100	0.79000600
C	-5.07079700	-0.93747000	1.75384000
H	-4.98437400	-0.16804300	2.52544300
C	-6.17893800	-1.77978200	1.73153100
H	-6.95219300	-1.65723700	2.49472400
C	-6.31977700	-2.77495200	0.75825800
C	-5.30469100	-2.88657000	-0.20027500
H	-5.38541100	-3.64856500	-0.98078700
C	-4.19360000	-2.04776800	-0.19817800
H	-3.42950400	-2.15655700	-0.96859600
C	-7.50351800	-3.70477700	0.74415400
H	-7.21367300	-4.71990300	1.06133500
H	-7.93241600	-3.79182800	-0.26580100
H	-8.29561400	-3.35743300	1.42265800
H	-3.17475600	0.70338400	1.35347600
O	2.44885700	4.16642800	0.35530800
H	2.65685500	4.89761600	-0.23853200
H	1.48513300	4.18602600	0.41466000
Ir	0.53627100	-0.16260800	-0.95291800

4·MeOH

P	1.02914600	-1.27900200	1.14882700
N	-1.46650700	-1.13826700	-0.24297500
N	-0.96513800	0.96273400	0.05111800
N	-3.04266500	0.26185200	0.94132000
C	1.79292900	-1.28629900	-2.35631700

C	0.49718000	-1.17532800	-2.96132100
C	0.14080000	0.21910100	-3.03026000
C	1.24780300	0.97195500	-2.47957900
C	2.25710900	0.05740900	-2.05326400
C	2.62317700	-2.52520500	-2.28090600
H	2.00753800	-3.42066600	-2.12809500
H	3.36139600	-2.47352100	-1.47090600
H	3.17272600	-2.65146700	-3.22789700
C	-0.33927500	-2.30803800	-3.45733300
H	-1.40935800	-2.10389400	-3.31678600
H	-0.09517300	-3.24369200	-2.93678800
H	-0.16117900	-2.46226200	-4.53354100
C	-1.08450600	0.78935300	-3.67025400
H	-0.93834900	0.90486900	-4.75605900
H	-1.32282600	1.77877000	-3.25690900
H	-1.95596300	0.13804700	-3.51475300
C	1.32252000	2.45997400	-2.39632300
H	1.91875100	2.77602100	-1.53183400
H	0.32483300	2.91142600	-2.31249400
H	1.79829000	2.85643200	-3.30765700
C	3.60913700	0.41283600	-1.52270100
H	4.33840800	0.45828600	-2.34796800
H	3.96594200	-0.33886200	-0.80506600
H	3.59842900	1.39059800	-1.02139900
C	1.63280600	-0.16629700	2.45670900
C	2.33264600	0.98788700	2.09124600
H	2.46742400	1.24232800	1.03756700
C	2.85356200	1.83456300	3.06929000
H	3.38674500	2.73468100	2.75726300
C	2.67428900	1.53159900	4.41809100
H	3.07637800	2.19612200	5.18641800
C	1.97961800	0.37832900	4.78874200
H	1.84013700	0.13820600	5.84516200
C	1.46476100	-0.47317400	3.81330900
H	0.93287900	-1.37880100	4.11581700
C	2.30005700	-2.58650200	1.06886800
C	3.64726100	-2.28621200	1.30376000
H	3.94493100	-1.28088600	1.61056700
C	4.62225700	-3.27103400	1.15015600
H	5.67057200	-3.02622900	1.33478900
C	4.26054100	-4.56303400	0.76942000
H	5.02551600	-5.33398400	0.65276600
C	2.91856900	-4.86934500	0.54404500
H	2.62836500	-5.88009100	0.24882300
C	1.94260100	-3.88544500	0.68751100
H	0.89895800	-4.13806500	0.48723100
C	-0.50885500	-2.08886500	1.79339700
H	-0.27698400	-3.01288100	2.34212100
H	-0.96021500	-1.38362500	2.50875900
C	-1.47517400	-2.31085600	0.62109300
H	-1.16917400	-3.17692700	0.01657800
H	-2.48336400	-2.53086600	1.00772400
C	-1.88738200	0.04340300	0.26026100
C	-1.12566000	2.35705100	0.11357700
C	-0.24919200	3.12257900	0.89093700
H	0.51056600	2.61188200	1.48433700
C	-0.35174400	4.51034500	0.91012200
H	0.34018000	5.08885600	1.53024200
C	-1.33239900	5.17923100	0.16324200

C	-2.21219200	4.40182300	-0.59881000
H	-2.98864800	4.89371000	-1.19113700
C	-2.11352000	3.01142400	-0.63178400
H	-2.80075900	2.42862200	-1.24903700
C	-1.45298200	6.67856700	0.21205800
H	-2.00019800	7.06672900	-0.65873200
H	-0.46479000	7.16090300	0.24208500
H	-1.99804100	6.99833900	1.11550800
C	-4.25623900	-0.44852000	0.84353400
C	-5.22315100	-0.24469200	1.83484100
H	-5.00935000	0.43370400	2.66488400
C	-6.45172100	-0.89403800	1.76294600
H	-7.19223900	-0.71425700	2.54708800
C	-6.75419700	-1.77191000	0.71449900
C	-5.77707000	-1.96155500	-0.26949500
H	-5.98387200	-2.63062100	-1.10944000
C	-4.54614900	-1.31161300	-0.21933400
H	-3.80939400	-1.47351400	-1.00705000
C	-8.06919700	-2.50244500	0.66260900
H	-8.02328300	-3.43994700	1.24134300
H	-8.34073600	-2.76782000	-0.36944700
H	-8.88316300	-1.89682000	1.08732200
H	-3.09152600	1.14890300	1.43491200
O	3.14860700	3.55213600	0.30116100
H	2.25708500	3.90889700	0.38923900
C	3.93295400	4.47131300	-0.42324800
H	4.94406800	4.05038200	-0.51564800
H	3.54256300	4.64586900	-1.44203500
H	4.01555600	5.44501400	0.09079700
Ir	0.39003400	-0.35602900	-0.92748800

5

Rh	0.73469000	-0.75246400	-0.46411100
P	2.33322700	0.24623000	0.81443300
N	-0.57223000	0.46342700	0.70106900
N	-2.17131900	-1.18603900	0.80037800
N	-2.73456600	0.94883900	1.52206600
C	-0.09196300	-2.20732500	-1.93013100
C	-0.52591500	-0.90290900	-2.31568100
C	0.62511300	-0.09434800	-2.62975400
C	1.77962800	-0.90716000	-2.42160400
C	1.35027200	-2.20504800	-1.95531400
C	-0.96193500	-3.39804200	-1.67084700
H	-1.97074700	-3.10435700	-1.35018800
H	-0.53398600	-4.05355800	-0.89988300
H	-1.07241700	-3.99542200	-2.59060700
C	-1.94101100	-0.45945100	-2.49382500
H	-2.19547100	-0.47102900	-3.56609800
H	-2.09339400	0.56842300	-2.13738700
H	-2.64846800	-1.11840700	-1.97612300
C	0.58282600	1.28537900	-3.20880600
H	1.54082100	1.80648600	-3.07737000
H	-0.20159700	1.89580900	-2.73786300
H	0.36744700	1.25140800	-4.28982900
C	3.18903900	-0.54276000	-2.75700400
H	3.42297400	-0.90083100	-3.77287800
H	3.90742500	-1.00714100	-2.06809500
H	3.34931600	0.54296900	-2.73822700

C	2.22701200	-3.40505500	-1.77813400
H	1.81234700	-4.10135100	-1.03639500
H	3.23839200	-3.12457100	-1.45389100
H	2.32431400	-3.94987700	-2.73172900
C	1.34196500	1.29138800	1.96455100
H	1.16025800	0.67136700	2.85634300
H	1.89693300	2.18826700	2.27499900
C	0.01068000	1.65250600	1.31113100
H	-0.65223500	2.07513100	2.07942100
H	0.15209000	2.43226500	0.54510600
C	3.48417800	1.37430200	-0.05041900
C	2.97508400	2.52391200	-0.67158500
H	1.91455400	2.77257500	-0.59466700
C	3.81237600	3.35747900	-1.40861500
H	3.40317300	4.25195600	-1.88353200
C	5.16649200	3.04686200	-1.54503400
H	5.82186400	3.69782600	-2.12803100
C	5.67848700	1.90362700	-0.93455900
H	6.73677100	1.65355700	-1.03731800
C	4.84325000	1.07081300	-0.18947500
H	5.26035400	0.17636900	0.27804200
C	3.41724100	-0.76040500	1.87794500
C	3.55326300	-2.13223900	1.64731500
H	2.98982900	-2.60346700	0.83949800
C	4.39976500	-2.89830600	2.44977400
H	4.50237600	-3.96929100	2.26164100
C	5.10642800	-2.29809200	3.49031200
H	5.76524500	-2.89876300	4.12168300
C	4.97165000	-0.92841500	3.72758300
H	5.52337000	-0.45589700	4.54324000
C	4.13456800	-0.16003800	2.92283400
H	4.04479100	0.91355300	3.10869900
C	-1.79481600	0.10442200	0.99308700
C	-3.46500700	-1.72292800	0.65160100
C	-4.52393600	-1.01382200	0.07298500
H	-4.38687400	0.01227900	-0.27110100
C	-5.76576500	-1.62718700	-0.08721600
H	-6.57919800	-1.05513000	-0.54135600
C	-5.98701300	-2.95426000	0.29464800
C	-4.91072200	-3.65507100	0.85601700
H	-5.04585900	-4.69640200	1.16087100
C	-3.67191200	-3.05225500	1.04153300
H	-2.84722200	-3.61350500	1.48757700
C	-7.33077300	-3.61029600	0.12494100
H	-7.83528100	-3.73301800	1.09718400
H	-7.23298200	-4.61322400	-0.31753600
H	-7.99122300	-3.01378100	-0.52021800
C	-3.01971700	2.26665400	1.11168400
C	-2.52436500	2.81562500	-0.07440200
H	-1.87193700	2.22903400	-0.72250600
C	-2.86471900	4.11894200	-0.43603800
H	-2.46346000	4.52727300	-1.36767100
C	-3.70701200	4.90582700	0.35409700
C	-4.20584800	4.33433400	1.53354500
H	-4.87138600	4.91841200	2.17531300
C	-3.86763000	3.04116200	1.91408800
H	-4.26249700	2.62016400	2.84222800
C	-4.06558200	6.31602400	-0.03148600
H	-3.65804500	6.57913500	-1.01788000

H	-3.67053800	7.03946300	0.69972700
H	-5.15716500	6.45635000	-0.06616700
H	-3.47011300	0.50038400	2.05997900
H	-1.38543100	-1.82760400	0.69985600
H	0.70818600	-1.73308200	0.76856900

5·CH_2O

Rh	-0.74968300	-0.51481500	0.60399200
P	-2.33365000	0.36790500	-0.78073000
N	0.56871300	0.57846000	-0.66533000
N	2.15629300	-1.08686900	-0.61931300
N	2.72535700	0.96222100	-1.55288700
C	0.08741400	-1.79835900	2.20808600
C	0.48142200	-0.44955300	2.46976600
C	-0.69441000	0.35189800	2.69301100
C	-1.82520800	-0.51169700	2.54766800
C	-1.35368300	-1.83530400	2.21863600
C	0.98194600	-2.99101100	2.08464700
H	2.01476000	-2.70375100	1.84532600
H	0.63417600	-3.68573300	1.30733500
H	1.00799900	-3.54242500	3.03879300
C	1.88330700	0.04805000	2.60621300
H	2.15922200	0.08028500	3.67272200
H	1.99274100	1.06658300	2.21021200
H	2.60291400	-0.60388400	2.09565700
C	-0.69803700	1.77602700	3.15268400
H	-1.67517100	2.25035000	2.99104400
H	0.06124400	2.37287800	2.62667900
H	-0.47368700	1.83714800	4.23057300
C	-3.24921600	-0.15221200	2.82072400
H	-3.49289600	-0.40695800	3.86508600
H	-3.94187300	-0.70632200	2.17303200
H	-3.43586100	0.92053700	2.68338600
C	-2.18398300	-3.07768500	2.14043100
H	-1.77486100	-3.78883500	1.40924000
H	-3.22389900	-2.85612500	1.86474600
H	-2.20306800	-3.58202100	3.12065200
C	-1.33100000	1.31953500	-2.00106600
H	-1.14170200	0.63786900	-2.84490500
H	-1.88100000	2.19313800	-2.37884700
C	-0.00535000	1.72433200	-1.36177500
H	0.66558700	2.09362400	-2.15007700
H	-0.15414600	2.55401800	-0.65168100
C	-3.50436500	1.55710600	-0.03432600
C	-2.99844500	2.73234300	0.53916300
H	-1.92887400	2.94974600	0.49920900
C	-3.84956800	3.62514100	1.18479300
H	-3.44333300	4.53883200	1.62432200
C	-5.21461500	3.34741800	1.27779400
H	-5.88168300	4.04455900	1.78978700
C	-5.72307000	2.17821400	0.71533800
H	-6.78990900	1.95457200	0.78516600
C	-4.87383300	1.28616200	0.05965000
H	-5.28820400	0.37330900	-0.37355900
C	-3.39363600	-0.73985900	-1.76403500
C	-3.64016200	-2.04246100	-1.31984600
H	-3.16994500	-2.39857700	-0.40106500
C	-4.47725600	-2.88696400	-2.04953900

H	-4.66608000	-3.90281900	-1.69539700
C	-5.06523100	-2.43538800	-3.23018000
H	-5.71653200	-3.09791600	-3.80482800
C	-4.82218600	-1.13580600	-3.67884700
H	-5.28270800	-0.77947400	-4.60291400
C	-3.99418700	-0.28767700	-2.94717500
H	-3.82138000	0.73225200	-3.29999500
C	1.78396100	0.17976300	-0.93536500
C	3.45163000	-1.61263600	-0.42801100
C	4.52500900	-0.84835900	0.04377000
H	4.40038800	0.21101900	0.27140600
C	5.76639300	-1.44850700	0.25001400
H	6.59212300	-0.83376300	0.61824000
C	5.97165800	-2.81305900	0.02165800
C	4.87873800	-3.56660100	-0.42773600
H	4.99981200	-4.63867900	-0.60551200
C	3.63915600	-2.98094300	-0.65976000
H	2.79363600	-3.58254000	-1.00389800
C	7.31717400	-3.45226500	0.23656500
H	7.84497900	-3.59154100	-0.72127400
H	7.21905600	-4.44527100	0.70004800
H	7.95831700	-2.83351300	0.88064300
C	3.04593300	2.30076800	-1.25015100
C	2.57049400	2.95307700	-0.10938700
H	1.90704800	2.43377500	0.58315200
C	2.94899000	4.26996700	0.15034800
H	2.56442900	4.75948600	1.04926200
C	3.81024600	4.96928800	-0.69971500
C	4.28724000	4.29513700	-1.83299400
H	4.96760700	4.80850300	-2.51812300
C	3.91178900	2.98639900	-2.11199400
H	4.29342400	2.48210700	-3.00357700
C	4.21143600	6.39370400	-0.42386300
H	3.85906100	6.72762700	0.56243900
H	3.79226800	7.07830200	-1.17888100
H	5.30548400	6.51355900	-0.45228600
H	3.44538300	0.45662300	-2.06027300
H	1.37375700	-1.70874000	-0.42258100
C	-0.36828700	-3.73143000	-1.65563700
O	0.51617600	-4.29386300	-1.06022800
H	-0.73179800	-1.60404900	-0.53217600
H	-0.16504900	-3.01217300	-2.48200300
H	-1.44248300	-3.92259600	-1.43579600

6

P	2.27152700	0.35398400	0.85688300
N	-0.62819000	0.52875200	0.77150800
N	-2.22901800	-1.12026100	0.90311700
N	-2.79060200	1.02905500	1.57647300
C	-0.17092300	-2.19757000	-1.85970300
C	-0.60725100	-0.89943400	-2.26015800
C	0.54236600	-0.09361400	-2.58727600
C	1.70257000	-0.90318800	-2.36563400
C	1.27764600	-2.19722500	-1.88846000
C	-1.03523200	-3.38627100	-1.57572900
H	-2.04454700	-3.08895800	-1.26004200
H	-0.60269900	-4.02076300	-0.78984900
H	-1.14165300	-4.00434400	-2.48204100

C	-2.02407100	-0.45902000	-2.43540600
H	-2.28683900	-0.48680400	-3.50513000
H	-2.17460700	0.57347500	-2.09206100
H	-2.72592600	-1.11085600	-1.90123000
C	0.49961100	1.27157100	-3.19990800
H	1.44909700	1.80520900	-3.05802100
H	-0.30093000	1.88441300	-2.76071700
H	0.31010100	1.20993200	-4.28456100
C	3.11083600	-0.53311100	-2.70285200
H	3.35175000	-0.89988900	-3.71368900
H	3.82886400	-0.98260400	-2.00364200
H	3.26143200	0.55402500	-2.69351800
C	2.14952700	-3.40120700	-1.71254200
H	1.75591400	-4.07209600	-0.93658800
H	3.17361400	-3.12021300	-1.43186400
H	2.20731500	-3.97277800	-2.65344700
C	1.28741800	1.40157500	2.01009600
H	1.12578100	0.79330600	2.91376500
H	1.83175100	2.31178000	2.29881000
C	-0.05527100	1.73363100	1.36564400
H	-0.72076300	2.15010300	2.13424000
H	0.06546100	2.50672500	0.58948200
C	3.39673000	1.47879800	-0.04597400
C	2.86071000	2.59694700	-0.70066900
H	1.79450200	2.82246700	-0.63020800
C	3.67762400	3.42636900	-1.46446300
H	3.24758000	4.29641100	-1.96567000
C	5.03812700	3.14173600	-1.59468300
H	5.67765200	3.78870200	-2.19934600
C	5.57679600	2.02975900	-0.95052900
H	6.64017900	1.80020700	-1.04849600
C	4.76184200	1.20216000	-0.17742300
H	5.19933000	0.33213300	0.31660500
C	3.38565100	-0.62653000	1.91487000
C	3.57276900	-1.98978300	1.66862400
H	3.02785100	-2.47268400	0.85480800
C	4.44700300	-2.73286400	2.46240900
H	4.58918600	-3.79711900	2.26217600
C	5.13140700	-2.11883000	3.50992900
H	5.81207500	-2.70187500	4.13462700
C	4.94640200	-0.75793200	3.76218800
H	5.48099600	-0.27433400	4.58273700
C	4.08092600	-0.01207100	2.96594600
H	3.95336200	1.05541000	3.16393700
C	-1.85368200	0.17214900	1.06664700
C	-3.51976900	-1.65942800	0.74121300
C	-4.57430400	-0.95531900	0.14898100
H	-4.43873400	0.07144200	-0.19346000
C	-5.80991300	-1.57609600	-0.03000100
H	-6.61962300	-1.00857400	-0.49629700
C	-6.02932200	-2.90459400	0.34772000
C	-4.95793500	-3.59905400	0.92617100
H	-5.09152800	-4.64119400	1.22892100
C	-3.72535500	-2.98946900	1.12945200
H	-2.90371700	-3.54636600	1.58645400
C	-7.36531100	-3.56961600	0.15319300
H	-7.88005900	-3.71181200	1.11730200
H	-7.25350000	-4.56467800	-0.30367200
H	-8.02337700	-2.96918000	-0.49076000

C	-3.07673700	2.33370000	1.12604000
C	-2.58653100	2.84091100	-0.08031400
H	-1.93787200	2.23046800	-0.71021300
C	-2.92724000	4.13165900	-0.48417500
H	-2.53138100	4.50783800	-1.43146600
C	-3.76395600	4.94577100	0.28417800
C	-4.25592100	4.41598800	1.48577600
H	-4.91591200	5.02303700	2.11170800
C	-3.91768500	3.13580500	1.90781200
H	-4.30684300	2.74732400	2.85225500
C	-4.12540200	6.34170800	-0.14773600
H	-3.71184600	6.57528800	-1.13896700
H	-3.73883900	7.08906700	0.56354600
H	-5.21735200	6.47591800	-0.19434600
H	-3.52516700	0.59361700	2.12647000
H	-1.44412200	-1.76609300	0.82704600
H	0.63416500	-1.65112700	0.91810200
Ir	0.67788000	-0.71343600	-0.39096300

6·CH_2O

P	2.28142900	0.42397700	0.83760200
N	-0.61407000	0.60635000	0.78099100
N	-2.22434800	-1.03450400	0.70591700
N	-2.78049900	1.03338400	1.61467500
C	-0.20135700	-1.79270700	-2.13763700
C	-0.58932300	-0.44455800	-2.41242000
C	0.58743700	0.35030000	-2.65610500
C	1.71935700	-0.52020100	-2.50365300
C	1.24618800	-1.84083700	-2.16720200
C	-1.09774400	-2.98106100	-1.99129000
H	-2.12772300	-2.68718200	-1.74838700
H	-0.74504200	-3.66358900	-1.20537200
H	-1.13108900	-3.54622600	-2.93694000
C	-1.99065700	0.05860100	-2.53635800
H	-2.27503400	0.09673400	-3.60019700
H	-2.09350800	1.07498600	-2.13363100
H	-2.70758000	-0.59331600	-2.02229200
C	0.59497300	1.76606200	-3.14046900
H	1.56793000	2.24556400	-2.96894700
H	-0.17470100	2.36872500	-2.63657400
H	0.38981100	1.80825000	-4.22315000
C	3.14274500	-0.16077200	-2.78461600
H	3.37774600	-0.39283100	-3.83597300
H	3.83756600	-0.73008800	-2.15254600
H	3.33234200	0.90828900	-2.62395900
C	2.06678700	-3.09020000	-2.09554000
H	1.67158100	-3.78567500	-1.34187700
H	3.11498000	-2.87268400	-1.84953700
H	2.05481200	-3.60936000	-3.06791800
C	1.30276400	1.34937000	2.09614400
H	1.14143800	0.65698600	2.93705800
H	1.85033600	2.22620100	2.46915500
C	-0.03893200	1.74662500	1.48863900
H	-0.70415900	2.08947400	2.29319800
H	0.08811100	2.59113600	0.79228100
C	3.42043300	1.62828100	0.06673500
C	2.88221900	2.78154000	-0.52180100
H	1.80730100	2.97185700	-0.48308600

C	3.70798000	3.68648000	-1.18309900
H	3.27684700	4.58282400	-1.63455100
C	5.07943700	3.44249100	-1.27747100
H	5.72648700	4.14893200	-1.80232000
C	5.61962900	2.29472700	-0.70093600
H	6.69137300	2.09665800	-0.77275000
C	4.79567600	1.39106700	-0.02870200
H	5.23427900	0.49480800	0.41500000
C	3.37335600	-0.68349300	1.78666400
C	3.61964600	-1.98193200	1.33025000
H	3.13176600	-2.33650300	0.41999500
C	4.47678500	-2.82451200	2.03820800
H	4.66471100	-3.83727300	1.67485000
C	5.08622200	-2.37524500	3.20893300
H	5.75359700	-3.03648300	3.76640700
C	4.84341000	-1.07999300	3.66975200
H	5.32004100	-0.72552400	4.58634600
C	3.99411400	-0.23403500	2.96009200
H	3.81991700	0.78194000	3.32367100
C	-1.84357100	0.22579900	1.02662200
C	-3.51950000	-1.55777600	0.50960100
C	-4.59136700	-0.79378800	0.03462900
H	-4.46751700	0.26628100	-0.18985200
C	-5.82997700	-1.39624100	-0.18204900
H	-6.65449900	-0.78221200	-0.55409600
C	-6.03345600	-2.76209800	0.03967100
C	-4.94215600	-3.51470200	0.49482900
H	-5.06198200	-4.58764500	0.66806900
C	-3.70558400	-2.92710700	0.73669800
H	-2.86072900	-3.52827500	1.08354400
C	-7.37508900	-3.40451100	-0.18929000
H	-7.90884900	-3.55347600	0.76374400
H	-7.26991900	-4.39342100	-0.65997500
H	-8.01412000	-2.78320300	-0.83297600
C	-3.06705600	2.37337700	1.28089100
C	-2.55642900	2.99511600	0.13825300
H	-1.88772500	2.45299400	-0.53146500
C	-2.90622800	4.31305400	-0.15430300
H	-2.49463800	4.77805900	-1.05431600
C	-3.77238500	5.04354500	0.66389300
C	-4.28340200	4.40033000	1.80025700
H	-4.96802500	4.93877800	2.46154600
C	-3.93665300	3.09104000	2.11202200
H	-4.34493500	2.61178500	3.00553600
C	-4.14359600	6.46855400	0.35182600
H	-3.76473400	6.77720900	-0.63285100
H	-3.72914300	7.16074600	1.10249200
H	-5.23582600	6.60686100	0.35427300
H	-3.52622100	0.55115100	2.10757800
H	-1.44105100	-1.65662700	0.50473500
C	0.30857500	-3.70426700	1.73415300
O	-0.58439800	-4.26473800	1.14992600
H	0.67114300	-1.57277600	0.66372000
H	0.11726700	-2.98691000	2.56481600
H	1.38002300	-3.89356600	1.49825300
Ir	0.68433600	-0.49647700	-0.53078200

7a

N	-0.69718000	-0.97493200	-1.02097000
N	-2.13271100	-0.75323100	0.78855900
N	-3.01028400	-1.10532300	-1.32735300
C	0.70339800	0.14843300	-2.72565600
C	-0.47048900	-0.82018700	-2.45740200
H	-0.25254800	-1.79711800	-2.91437200
H	-1.37750000	-0.43371800	-2.94657500
C	-1.90895500	-0.94932400	-0.53093300
C	-1.30751500	-0.04613100	1.69672800
C	0.08858100	-0.00995300	1.57310600
C	0.78854700	0.74151500	2.52309500
H	1.87909300	0.80112400	2.44458900
C	0.17027300	1.42356900	3.57836100
C	-1.22201000	1.34568800	3.67895900
H	-1.74364700	1.86077300	4.48991300
C	-1.95462200	0.62268100	2.74276300
H	-3.04471900	0.57207200	2.82109500
C	0.98941400	2.23791500	4.54467300
H	1.89417700	1.69657600	4.86153300
H	1.32227900	3.18156400	4.08077600
H	0.41511900	2.49810900	5.44553800
C	-4.32904800	-0.68428800	-1.04315000
C	-4.59277300	0.53783100	-0.41501200
H	-3.77203500	1.19621000	-0.12309000
C	-5.90921700	0.92217300	-0.16645100
H	-6.09351900	1.87774500	0.33167800
C	-6.99198100	0.12565400	-0.55423200
C	-6.70939400	-1.08486800	-1.20180700
H	-7.53251300	-1.73052400	-1.52019200
C	-5.40070400	-1.49414500	-1.43521000
H	-5.20305900	-2.45082100	-1.92525600
C	-8.41403000	0.54743300	-0.29871800
H	-8.96015500	-0.22028100	0.27130300
H	-8.45942900	1.48785400	0.26826000
H	-8.95915000	0.69580600	-1.24439400
H	-3.08611600	-0.90099000	1.09965500
H	-2.89395600	-1.68558600	-2.15057400
H	1.56879400	-0.38737100	-3.14350200
H	0.42911600	0.94175400	-3.43609300
P	1.28137100	0.82705200	-1.12069300
C	2.90840800	1.58836600	-1.40321800
C	3.38105800	1.89703300	-2.68442000
C	3.71131300	1.85989800	-0.28774300
C	4.64429000	2.46560700	-2.84331000
H	2.77130300	1.69530400	-3.56746700
C	4.96781700	2.43945900	-0.44947800
H	3.35482500	1.60743800	0.71385800
C	5.43721900	2.73845000	-1.72862100
H	5.00913800	2.69946200	-3.84585400
H	5.58518600	2.64969800	0.42655300
H	6.42549500	3.18553300	-1.85756400
C	0.16115200	2.21964200	-0.72379200
C	-1.10555200	2.31968000	-1.31197100
C	0.52730700	3.15110700	0.25709500
C	-1.98835400	3.32807300	-0.92371800
H	-1.42859400	1.61697500	-2.08139600
C	-0.35750200	4.15251100	0.64762900
H	1.50864500	3.09853300	0.73158500
C	-1.62038600	4.24142400	0.06164600

H	-2.97034700	3.39525500	-1.39726700
H	-0.05750500	4.86561400	1.41852700
H	-2.31441000	5.02640000	0.37016900
Rh	1.05207500	-1.08978000	0.14397600
C	2.89433500	-2.21295500	-0.47116100
C	2.97818400	-1.78270500	0.91861100
C	1.94459200	-2.45783800	1.64736600
C	1.78606300	-3.08083000	-0.59009100
C	1.16810600	-3.21285400	0.72086200
C	3.87693000	-1.85660200	-1.54078000
C	4.11917000	-1.03536200	1.53289400
C	1.75124400	-2.42920900	3.12761800
C	1.33691300	-3.80693500	-1.81712500
C	0.04141300	-4.13132600	1.07457300
H	3.43139800	-1.92138500	-2.54333300
H	4.73315500	-2.54992200	-1.51189500
H	4.27069200	-0.83963100	-1.40730100
H	3.78508300	-0.36799500	2.34003100
H	4.65730600	-0.43357000	0.78985400
H	4.84011700	-1.74578400	1.97016600
H	2.15435400	-1.51084400	3.57388600
H	2.27656100	-3.28392600	3.58431500
H	0.68934400	-2.50139300	3.39895300
H	1.69657100	-4.84841600	-1.79495600
H	1.72562600	-3.34026600	-2.73228900
H	0.24002400	-3.84133600	-1.88433900
H	-0.59639300	-3.69893800	1.85826500
H	0.43207800	-5.09107400	1.45069300
H	-0.59174500	-4.35196500	0.20445300

7b

N	1.30815000	0.27611900	0.06061000
N	1.21287300	2.12060400	-1.35049800
N	3.07618800	1.84192200	-0.04817300
C	0.57846800	-0.71749100	2.21201800
C	1.77030200	-0.28699000	1.32909200
H	2.41925300	-1.15571100	1.14419800
H	2.37904500	0.45036900	1.87171300
C	1.85147400	1.35836300	-0.43156300
C	-0.18235200	2.20785800	-1.57771200
C	-1.05144500	1.13094000	-1.35474900
C	-2.41032400	1.35312700	-1.61023200
H	-3.11814700	0.53831200	-1.42921700
C	-2.92118400	2.56522500	-2.08719800
C	-2.01819000	3.60932400	-2.31479200
H	-2.37507500	4.57247200	-2.68922800
C	-0.66400000	3.43358800	-2.05596500
H	0.03661100	4.25653800	-2.22597200
C	-4.40029100	2.74735100	-2.30313100
H	-4.88563200	1.80360700	-2.59405200
H	-4.89271500	3.09691000	-1.37985400
H	-4.60720900	3.49319900	-3.08466300
C	4.24092400	1.10311900	0.25072200
C	4.46440300	-0.17516600	-0.26939400
H	3.72312700	-0.63225000	-0.92538500
C	5.62933400	-0.86607100	0.05065600
H	5.77904000	-1.86783500	-0.36171800
C	6.61196900	-0.30497100	0.87574000

C	6.38085500	0.98318100	1.37276100
H	7.12668900	1.45241000	2.01997600
C	5.21127700	1.67867800	1.07601200
H	5.04424400	2.67524000	1.49221100
C	7.87820100	-1.05485600	1.19157300
H	8.39351700	-0.62484500	2.06225500
H	7.67380700	-2.11517900	1.40291000
H	8.57951900	-1.02228600	0.34168400
H	1.77498200	2.84305800	-1.78669600
H	3.20311500	2.84509300	-0.14047400
H	0.52758800	-1.81350400	2.29551100
H	0.65599800	-0.31257800	3.23154800
P	-0.97900200	-0.21800700	1.38627100
C	-2.34408600	-1.10406800	2.19605500
C	-2.20752100	-1.70589600	3.45242300
C	-3.56887700	-1.18529800	1.52079400
C	-3.28438000	-2.38686100	4.01938700
H	-1.26272000	-1.65147100	3.99755300
C	-4.64576100	-1.85697800	2.09499300
H	-3.67624300	-0.73019700	0.53322700
C	-4.50223300	-2.46280600	3.34356400
H	-3.17022300	-2.85994400	4.99721500
H	-5.59677100	-1.91524900	1.56115500
H	-5.34285300	-2.99696100	3.79211100
C	-1.23987200	1.54175100	1.81049400
C	-0.18632200	2.34267600	2.26669400
C	-2.49116400	2.12968900	1.58295600
C	-0.37835600	3.70854100	2.47680800
H	0.80043800	1.91939700	2.46170000
C	-2.68053200	3.49244300	1.79398800
H	-3.32878300	1.52494100	1.23066000
C	-1.62223800	4.28738200	2.23532200
H	0.45326000	4.32107000	2.83240600
H	-3.66065500	3.93671400	1.60724000
H	-1.77033100	5.35764000	2.39573300
C	-0.73277800	-2.91732900	-0.84363700
C	-1.61923400	-2.20487900	-1.75051000
C	-0.80429900	-1.59604000	-2.76480100
C	0.59850800	-2.68168000	-1.25392500
C	0.56073700	-1.83604300	-2.43784900
C	-1.17347500	-3.80911700	0.27277600
C	-3.10602100	-2.35839400	-1.81661600
C	-1.30642100	-0.88911900	-3.98063300
C	1.83288200	-3.26817400	-0.64614200
C	1.73973700	-1.43605900	-3.26661900
H	-0.42247900	-3.86377200	1.07288400
H	-1.33080300	-4.83207800	-0.10612000
H	-2.11902500	-3.47065900	0.71660200
H	-3.59946400	-1.43677900	-2.15557200
H	-3.53084200	-2.64145700	-0.84518600
H	-3.36818500	-3.15298300	-2.53472700
H	-2.27197100	-0.40012300	-3.79556500
H	-1.44687900	-1.61352400	-4.79947600
H	-0.59883600	-0.12320800	-4.32579700
H	2.10078300	-4.20887200	-1.15416800
H	1.69240700	-3.49554300	0.41958200
H	2.69231100	-2.59114300	-0.74106000
H	1.60756400	-0.43299900	-3.69611200
H	1.88139800	-2.14278300	-4.10032900

H	2.66606600	-1.43415300	-2.67719500
Rh	-0.35948200	-0.69695600	-0.78478400

8a

N	-0.78786500	-0.87554300	-1.00646600
N	-2.21935700	-0.66738100	0.80969700
N	-3.09206700	-1.10373400	-1.29310700
C	0.54063900	0.29059900	-2.74626100
C	-0.58549200	-0.72580600	-2.44912600
H	-0.33077300	-1.69927200	-2.89432600
H	-1.51474400	-0.38394100	-2.92759700
C	-1.99741600	-0.88743300	-0.50312300
C	-1.39680800	0.05502200	1.71039700
C	0.00087200	0.09592300	1.58076600
C	0.69245300	0.85223400	2.53680100
H	1.78367500	0.91492500	2.46165100
C	0.07030300	1.53505800	3.58836800
C	-1.32195400	1.45530600	3.68621800
H	-1.84698900	1.97206000	4.49389900
C	-2.04908100	0.72534900	2.75115900
H	-3.13888500	0.66936100	2.82822100
C	0.88543300	2.35402000	4.55413100
H	1.79305400	1.81735300	4.87079800
H	1.21337100	3.29906900	4.08952800
H	0.31021600	2.61197900	5.45507400
C	-4.42016400	-0.69819500	-1.03364200
C	-4.71029700	0.53488700	-0.44009100
H	-3.90280300	1.21196100	-0.15477300
C	-6.03528500	0.90429500	-0.21642600
H	-6.24050000	1.86850200	0.25629600
C	-7.10085900	0.08166800	-0.59744300
C	-6.79155700	-1.13965400	-1.21167500
H	-7.60085500	-1.80531500	-1.52432800
C	-5.47392700	-1.53386200	-1.41963100
H	-5.25494600	-2.49829600	-1.88487400
C	-8.53224000	0.48721400	-0.36893700
H	-9.07924300	-0.28467000	0.19443300
H	-8.59872700	1.42948200	0.19289500
H	-9.06237700	0.62566800	-1.32465800
H	-3.16037900	-0.85513900	1.13667400
H	-2.95619100	-1.70978600	-2.09445200
H	1.40316400	-0.20520400	-3.21535100
H	0.20774300	1.08956800	-3.42412300
P	1.16046200	0.95030300	-1.15032000
C	2.77179900	1.73218000	-1.46000000
C	3.19861400	2.08379500	-2.74633700
C	3.60104200	1.99428800	-0.36191700
C	4.44404100	2.68364700	-2.92787900
H	2.56649500	1.89196300	-3.61577500
C	4.83963000	2.60496700	-0.54596300
H	3.27796300	1.71234400	0.64305900
C	5.26387000	2.94609900	-1.83021600
H	4.77334700	2.95082900	-3.93439200
H	5.47814200	2.80748200	0.31660300
H	6.23777100	3.41862400	-1.97666900
C	0.03536400	2.32452400	-0.70661600
C	-1.25408800	2.40537400	-1.24636900
C	0.42119600	3.25294100	0.26895800

C	-2.14104300	3.39119100	-0.81284200
H	-1.59016200	1.70471000	-2.01266400
C	-0.46774100	4.23185700	0.70479300
H	1.42201000	3.21578700	0.70267600
C	-1.75385900	4.30049200	0.16908700
H	-3.14165600	3.44433200	-1.24775100
H	-0.15266100	4.94295500	1.47150500
H	-2.45165400	5.06687600	0.51374600
Ir	0.99700200	-0.98553400	0.13936700
C	2.80629700	-2.14938800	-0.57251600
C	2.99263000	-1.67358800	0.79470000
C	2.00852600	-2.31596100	1.61692200
C	1.68980900	-3.01950300	-0.58043600
C	1.16348200	-3.10667300	0.77501300
C	3.71248400	-1.82400400	-1.71770200
C	4.17967300	-0.91679200	1.30084200
C	1.92136400	-2.22680300	3.10474100
C	1.14318000	-3.77890300	-1.74639200
C	0.08035000	-4.02904900	1.24080000
H	3.23310300	-2.02468000	-2.68552800
H	4.62671700	-2.43670800	-1.66399700
H	4.02135000	-0.76910400	-1.69975100
H	3.90883300	-0.23888300	2.12296900
H	4.65047000	-0.32470800	0.50606100
H	4.93698500	-1.61951300	1.68587700
H	2.30754400	-1.26890700	3.47648000
H	2.52238500	-3.03223900	3.55681600
H	0.88667200	-2.33616400	3.45608800
H	1.47450800	-4.82875300	-1.70397000
H	1.48350100	-3.35836600	-2.70197800
H	0.04349100	-3.78050100	-1.74064600
H	-0.47125900	-3.59841600	2.08801700
H	0.50519400	-4.99219100	1.56772800
H	-0.64143300	-4.24080200	0.44018900

8b

N	1.34100600	0.37024000	0.13843600
N	1.24540500	2.11031200	-1.40219500
N	3.11907100	1.90807300	-0.10571600
C	0.60291000	-0.40908300	2.37967100
C	1.79764600	-0.07967800	1.45597700
H	2.43256700	-0.97182500	1.34827300
H	2.41736400	0.69812500	1.92399000
C	1.88699200	1.41147400	-0.44013900
C	-0.15044500	2.18011100	-1.63903700
C	-1.01304800	1.10878400	-1.35877000
C	-2.36925700	1.30955800	-1.65485500
H	-3.07623100	0.50258100	-1.43782700
C	-2.87988200	2.48828600	-2.20864300
C	-1.98265000	3.52734300	-2.47573600
H	-2.34076800	4.46646400	-2.90584800
C	-0.63136700	3.37507900	-2.18710400
H	0.06679300	4.19246300	-2.38996300
C	-4.35638600	2.64352600	-2.45939000
H	-4.81902100	1.68905900	-2.75247500
H	-4.87475700	2.99155400	-1.54984800
H	-4.55824700	3.37903600	-3.25185000
C	4.28168100	1.18487100	0.23370000
C	4.46580100	-0.15019600	-0.13659200
H	3.69130000	-0.66963100	-0.70099900

C	5.63388100	-0.81906700	0.21872100
H	5.75209300	-1.86584100	-0.07479200
C	6.65706400	-0.18196800	0.93096500
C	6.46329700	1.16048600	1.27895500
H	7.24118800	1.69000300	1.83550500
C	5.29329200	1.83706300	0.94530400
H	5.15842200	2.88041100	1.24127800
C	7.92857500	-0.90708700	1.28206800
H	8.43625400	-0.43685500	2.13655400
H	7.73400100	-1.96011200	1.53391300
H	8.63407700	-0.89944900	0.43482000
H	1.81425100	2.77891500	-1.90916400
H	3.25233000	2.90060900	-0.27485400
H	0.56609400	-1.48594300	2.60134400
H	0.66370700	0.12535000	3.33853300
P	-0.94811500	-0.03649700	1.47852600
C	-2.31928700	-0.87656500	2.32256400
C	-2.22154300	-1.33178600	3.64197800
C	-3.51153800	-1.06407500	1.61149000
C	-3.30458100	-1.97898600	4.23629300
H	-1.30313100	-1.18613800	4.21497200
C	-4.59501600	-1.70073300	2.21195300
H	-3.58439000	-0.72114500	0.57635200
C	-4.48959900	-2.16380600	3.52397100
H	-3.22200200	-2.33903000	5.26414400
H	-5.52087600	-1.84398200	1.65058100
H	-5.33509000	-2.67110500	3.99403300
C	-1.26023000	1.74778000	1.73193900
C	-0.22681900	2.61461400	2.10415200
C	-2.52537300	2.27961800	1.45166200
C	-0.45083600	3.98936300	2.18057500
H	0.76842500	2.23291100	2.33818600
C	-2.74723500	3.65172500	1.52795500
H	-3.34786500	1.62326000	1.16171200
C	-1.70855000	4.51136100	1.88662000
H	0.36585200	4.65334000	2.47269600
H	-3.73771300	4.05178000	1.30029700
H	-1.88288700	5.58827500	1.94099000
Ir	-0.32065700	-0.70145100	-0.65626400
C	-0.69797100	-2.93423600	-0.56095400
C	-1.57056900	-2.29483900	-1.53514200
C	-0.73200600	-1.76510100	-2.58000100
C	0.64366500	-2.73618400	-0.96911300
C	0.62856900	-1.99067100	-2.22014500
C	-1.15800500	-3.74120800	0.61075500
C	-3.05491100	-2.46607500	-1.62723700
C	-1.21588000	-1.14110400	-3.84742400
C	1.86759600	-3.28580400	-0.30755900
C	1.82232900	-1.67388800	-3.06454200
H	-0.41887400	-3.73934900	1.42342000
H	-1.31342300	-4.78801400	0.30318700
H	-2.10910900	-3.36854000	1.01245800
H	-3.54512300	-1.56957400	-2.03161600
H	-3.49908600	-2.69003900	-0.64894500
H	-3.29619100	-3.30534800	-2.30022300
H	-2.16948200	-0.61708500	-3.69944300
H	-1.37287300	-1.92304900	-4.60803000
H	-0.49035400	-0.41962200	-4.24647600
H	2.13787500	-4.25840400	-0.74971300

H	1.71316800	-3.44039100	0.76898800
H	2.72874200	-2.61669500	-0.43705300
H	1.70236600	-0.71519400	-3.58807000
H	1.97348000	-2.45716000	-3.82485200
H	2.73919000	-1.61927300	-2.46270900

H_2O

O	0.00000000	0.00000000	0.12021700
H	0.00000000	0.75355100	-0.48086700
H	0.00000000	-0.75355100	-0.48086700

I^{Ir}

P	-2.27861600	0.15450300	-0.87102700
N	0.54201600	0.00420000	-1.21850000
N	2.50783200	-1.19294600	-0.94254900
N	2.62774200	1.07772000	-1.56559300
C	0.53346800	-2.38155300	1.57964400
C	0.69581900	-1.10700600	2.15372400
C	-0.63698900	-0.54343800	2.36834000
C	-1.60890900	-1.54873500	2.02951500
C	-0.90014100	-2.63881000	1.44884600
C	1.58833900	-3.37094500	1.22008100
H	2.59001900	-2.92636100	1.23304900
H	1.41525700	-3.79097100	0.22001200
H	1.56572800	-4.20210300	1.94349000
C	1.97371500	-0.43922500	2.52985300
H	2.10353100	-0.48271400	3.62310300
H	1.97873200	0.62156800	2.24391900
H	2.83513900	-0.93073700	2.06313000
C	-0.91016600	0.76861300	3.02560200
H	-1.89839800	1.15949400	2.74743600
H	-0.15229300	1.51440000	2.74783100
H	-0.88290400	0.65348300	4.12121800
C	-3.08079900	-1.48390100	2.27831200
H	-3.31912600	-2.04468100	3.19577900
H	-3.65835100	-1.92633300	1.45535100
H	-3.41584600	-0.45001100	2.42504700
C	-1.47054900	-3.94169200	0.99647000
H	-0.99882400	-4.28385200	0.06453300
H	-2.55567200	-3.88263700	0.84638100
H	-1.27962400	-4.70854600	1.76482600
C	-1.50502000	0.63570600	-2.45804600
H	-1.64419600	-0.24817800	-3.10008300
H	-2.01843400	1.48535600	-2.93063300
C	-0.02471800	0.90297800	-2.24235000
H	0.51016500	0.76356200	-3.19352000
H	0.11785100	1.95847300	-1.96283600
C	-2.87067300	1.63973500	0.00191000
C	-2.07689600	2.79520600	-0.00945900
H	-1.16269200	2.84018300	-0.60388100
C	-2.43631700	3.90198400	0.75579000
H	-1.81154900	4.79767300	0.73383100
C	-3.58561400	3.86477600	1.54574400
H	-3.86514000	4.73253500	2.14727200
C	-4.37838500	2.71787900	1.56225000
H	-5.28149500	2.68301000	2.17530700
C	-4.02451200	1.60866100	0.79501700
H	-4.65495300	0.71793400	0.82109400

C	-3.73293500	-0.82667400	-1.34556800
C	-3.61700600	-2.22007200	-1.39214600
H	-2.68439000	-2.69594200	-1.08335400
C	-4.68362400	-2.99935900	-1.83636700
H	-4.58586900	-4.08669400	-1.86506400
C	-5.86920600	-2.38869900	-2.24352300
H	-6.70732400	-2.99835400	-2.58861200
C	-5.98326500	-0.99798900	-2.21717800
H	-6.90680800	-0.51728800	-2.54683600
C	-4.91877600	-0.21535300	-1.77466500
H	-5.01849200	0.87258400	-1.76618400
C	1.93782500	-0.07219000	-1.21013600
C	3.88623600	-1.30855200	-0.73632600
C	4.63634100	-0.43502700	0.07595000
H	4.14803300	0.41604700	0.55512000
C	5.99384700	-0.65224600	0.28869200
H	6.54771600	0.04126900	0.92852100
C	6.66526800	-1.73676400	-0.29301600
C	5.91462700	-2.60704200	-1.09122800
H	6.40426900	-3.46751400	-1.55620900
C	4.55200000	-2.40544900	-1.30242600
H	3.97848800	-3.10153800	-1.91893600
C	8.13545600	-1.95227700	-0.04928500
H	8.72085300	-1.06002800	-0.32205800
H	8.52015200	-2.80092900	-0.63253100
H	8.33748300	-2.15691200	1.01452700
C	2.40901300	2.36696100	-1.04851400
C	1.61835200	2.60452100	0.08137900
H	1.13175600	1.77270000	0.59213600
C	1.43014700	3.90472300	0.54874900
H	0.80017000	4.06083100	1.42892900
C	2.03077000	5.00349900	-0.07191200
C	2.83664100	4.74913000	-1.19141000
H	3.32569300	5.58321200	-1.70250600
C	3.02067700	3.46011500	-1.67857600
H	3.64078800	3.28983600	-2.56228700
C	1.83362600	6.40660700	0.43736000
H	1.10166600	6.43730900	1.25718500
H	1.47639000	7.07510500	-0.36152500
H	2.77811600	6.83092600	0.81398500
H	3.57137000	0.91674000	-1.90520000
Ir	-0.50554400	-0.83572500	0.25200700

I^Ir^·H_2O

P	-2.26579800	0.01736600	-0.89681800
N	0.56731200	0.12594500	-1.08327000
N	2.57487000	-0.98726900	-0.83412600
N	2.61293400	1.31357500	-1.30594800
C	0.68154200	-1.98430700	1.89123600
C	0.61887700	-0.66303900	2.37228900
C	-0.79284800	-0.28625500	2.46637900
C	-1.58520400	-1.44542600	2.15159700
C	-0.68914900	-2.45576400	1.69437000
C	1.89006700	-2.83437400	1.69845000
H	2.81245100	-2.24111500	1.68808800
H	1.82398100	-3.39587500	0.75807000
H	1.95496200	-3.55724800	2.52814400
C	1.76669600	0.20563100	2.75921600
H	1.91103100	0.16112200	3.85063000

H	1.59086500	1.25657600	2.49415800
H	2.69732400	-0.12475100	2.28165400
C	-1.29404200	1.00991800	3.01137800
H	-2.30939800	1.23250000	2.65733400
H	-0.63748400	1.84272900	2.72337600
H	-1.31697800	0.96831600	4.11246500
C	-3.06483900	-1.57675600	2.31081500
H	-3.28587600	-2.01509600	3.29673700
H	-3.50325200	-2.23114800	1.54594300
H	-3.56343300	-0.60119900	2.26234200
C	-1.01967100	-3.84748600	1.26901400
H	-0.46082000	-4.11131700	0.35875100
H	-2.09430900	-3.97726700	1.08838000
H	-0.72390900	-4.55306700	2.06203400
C	-1.44908500	0.47969500	-2.47004300
H	-1.46716800	-0.44700100	-3.06503300
H	-2.01225500	1.24814800	-3.01875500
C	-0.01376600	0.90124900	-2.19525400
H	0.58861100	0.74504200	-3.10335000
H	0.01953500	1.98170400	-1.98303400
C	-3.06209800	1.48676100	-0.17233400
C	-2.39752300	2.71930500	-0.23088800
H	-1.46307700	2.82410300	-0.78517900
C	-2.91602200	3.82956400	0.43139200
H	-2.39020100	4.78517300	0.37364600
C	-4.09892200	3.72011700	1.16289700
H	-4.50334300	4.59068600	1.68395600
C	-4.76657700	2.49752700	1.22182400
H	-5.69627400	2.40598900	1.78747000
C	-4.25232600	1.38408500	0.55841700
H	-4.78775600	0.43418800	0.61491500
C	-3.58630600	-1.13894500	-1.36340100
C	-3.35955400	-2.51322700	-1.23849900
H	-2.42083000	-2.86916700	-0.81095600
C	-4.32762500	-3.42548700	-1.65482800
H	-4.14439200	-4.49688700	-1.54860300
C	-5.52483300	-2.96666400	-2.20266000
H	-6.28695500	-3.67967000	-2.52495300
C	-5.74903100	-1.59618100	-2.34496300
H	-6.68292000	-1.23590600	-2.78164300
C	-4.78296600	-0.68146500	-1.93183600
H	-4.96799200	0.38885000	-2.05064100
C	1.96266500	0.12997700	-1.04083000
C	3.95872400	-1.09909400	-0.64593500
C	4.71651200	-0.25978500	0.19154300
H	4.23668400	0.56914100	0.71615700
C	6.07965200	-0.48028400	0.36718400
H	6.64357200	0.18634000	1.02629600
C	6.74357400	-1.53321500	-0.27702400
C	5.98107300	-2.37227100	-1.09750900
H	6.46436300	-3.20983000	-1.60838100
C	4.61488500	-2.16789100	-1.27369700
H	4.02940800	-2.84118300	-1.90402800
C	8.22013500	-1.74993500	-0.07787400
H	8.79646600	-0.85405400	-0.35821900
H	8.58794900	-2.59135200	-0.68207100
H	8.45245500	-1.96603400	0.97720900
C	2.23895300	2.62051500	-0.94655400
C	1.29499200	2.89874800	0.04778800

H	0.79778700	2.08111000	0.57036700
C	0.97659300	4.21872700	0.36322300
H	0.23218500	4.40725500	1.14192900
C	1.59062300	5.29813100	-0.27948000
C	2.54789400	5.00371200	-1.26048800
H	3.05341700	5.82152600	-1.78152800
C	2.86510900	3.69249600	-1.59674400
H	3.60669100	3.48894700	-2.37353700
C	1.23369900	6.72307900	0.04894900
H	0.63510600	6.78660000	0.96898700
H	0.64796400	7.18218800	-0.76417800
H	2.13493000	7.33991400	0.18511300
H	3.58425100	1.21531300	-1.58708200
Ir	-0.46971300	-0.70578600	0.39282400
O	1.20958300	-3.36189500	-1.53086900
H	1.65145800	-2.52266800	-1.26126400
H	0.45835000	-3.05552200	-2.04927800

$\mathbf{I}^{\mathbf{Ir}}$·MeOH

P	-2.30255700	0.03783800	-0.84164600
N	0.52322100	0.17689600	-1.06208500
N	2.45934900	-1.03853300	-0.78573200
N	2.62741000	1.20844400	-1.45652500
C	0.69442400	-1.63301300	2.10924000
C	0.61902100	-0.27256400	2.46170800
C	-0.79756400	0.09275600	2.53715600
C	-1.57667200	-1.10108900	2.34048800
C	-0.67044200	-2.13916900	1.97482700
C	1.91158300	-2.48514600	2.00013100
H	2.82474900	-1.88851600	1.89055800
H	1.83838200	-3.17662600	1.15120000
H	2.00586600	-3.08749600	2.91865100
C	1.76232300	0.63359800	2.76645000
H	1.94083400	0.64060600	3.85391500
H	1.56151500	1.66809700	2.45970300
H	2.68405400	0.29506100	2.27710300
C	-1.31802600	1.42319300	2.97090900
H	-2.33868600	1.59644100	2.60341100
H	-0.67862700	2.23922700	2.60660900
H	-1.33621900	1.47605500	4.07152800
C	-3.05405600	-1.23399900	2.51938700
H	-3.26666800	-1.57357300	3.54528400
H	-3.48800500	-1.96621000	1.82569300
H	-3.56489700	-0.27404400	2.37579000
C	-0.98347000	-3.56492700	1.66901300
H	-0.45457900	-3.88040600	0.75631600
H	-2.06085300	-3.72930000	1.54496100
H	-0.63884100	-4.20307100	2.49855200
C	-1.50031600	0.47108800	-2.43324300
H	-1.48658500	-0.46894400	-3.00612800
H	-2.08535000	1.21013300	-2.99881700
C	-0.07925100	0.94209300	-2.16707900
H	0.52137000	0.81425600	-3.07984900
H	-0.07803900	2.02223100	-1.94676700
C	-3.17649900	1.50397100	-0.20261500
C	-2.51412700	2.74003500	-0.21516900
H	-1.52970500	2.83912000	-0.67814400
C	-3.09809200	3.85547900	0.37860500

H	-2.57512300	4.81406400	0.35858400
C	-4.34315300	3.74680400	0.99998400
H	-4.79857400	4.62114300	1.47014100
C	-5.00515900	2.52028200	1.01699600
H	-5.98073100	2.42939300	1.49945100
C	-4.42682200	1.40113100	0.41734300
H	-4.95672500	0.44662600	0.44092200
C	-3.56522800	-1.19133000	-1.27827200
C	-3.38459400	-2.53503100	-0.93944400
H	-2.51469000	-2.83476900	-0.35428900
C	-4.30814500	-3.49296900	-1.35688200
H	-4.16074200	-4.54101200	-1.08766200
C	-5.41106700	-3.11098100	-2.11858300
H	-6.13429700	-3.86068500	-2.44716700
C	-5.59026400	-1.77095700	-2.46837200
H	-6.45077900	-1.47175500	-3.07063500
C	-4.67047900	-0.81161800	-2.05343900
H	-4.81648200	0.23461000	-2.33409300
C	1.91215700	0.09738600	-1.06995400
C	3.83307000	-1.22465700	-0.58629000
C	4.64978900	-0.35738500	0.16305300
H	4.23279400	0.55813100	0.58736800
C	5.98798700	-0.66525900	0.38986900
H	6.59685700	0.02307100	0.98348600
C	6.56923300	-1.83623500	-0.11583700
C	5.74902000	-2.69876200	-0.85206600
H	6.16597900	-3.62746700	-1.25171500
C	4.40604700	-2.40666800	-1.07720600
H	3.77000700	-3.10414900	-1.62701100
C	8.02038800	-2.14764600	0.13707800
H	8.67622900	-1.38539300	-0.31328500
H	8.30115700	-3.12359600	-0.28368300
H	8.24263700	-2.16729400	1.21569700
C	2.38536800	2.55863800	-1.14747800
C	1.45577500	2.97615400	-0.18783200
H	0.85953900	2.24167200	0.35372700
C	1.28771200	4.33356700	0.08363100
H	0.55382200	4.63114700	0.83802800
C	2.03849900	5.31484600	-0.56983600
C	2.97400100	4.88155500	-1.52000900
H	3.58343200	5.61863800	-2.05035600
C	3.14416200	3.53309100	-1.81144600
H	3.87650800	3.22239800	-2.56121700
C	1.86099300	6.77961800	-0.27149600
H	1.10971200	6.94138100	0.51462900
H	1.53770000	7.33264900	-1.16774600
H	2.80549600	7.23538600	0.06508700
H	3.57191600	1.01570400	-1.77703500
O	1.01619900	-3.41146800	-1.15785000
H	1.47137100	-2.55687600	-0.96944500
C	0.15401200	-3.20562700	-2.23746900
H	-0.26312900	-4.17477700	-2.55436600
H	-0.69937200	-2.54502800	-1.98910900
H	0.66814800	-2.76433400	-3.11357600
Ir	-0.48774400	-0.50632400	0.51218800

I^{Rh}

Rh	-0.58337300	-0.89781000	0.30907900

P	-2.35611500	0.03247500	-0.86722900
N	0.47529300	-0.11897100	-1.19146100
N	2.42277600	-1.32519500	-0.87351600
N	2.57308700	0.92563200	-1.56415200
C	0.48753800	-2.26011900	1.75254400
C	0.63737900	-0.92857600	2.17849600
C	-0.69835800	-0.36487200	2.35221700
C	-1.66115500	-1.41569700	2.13608900
C	-0.94178400	-2.55273400	1.68309000
C	1.55144300	-3.26810100	1.48627000
H	2.54971300	-2.81641300	1.47581500
H	1.39301900	-3.76897500	0.52156300
H	1.52441300	-4.03901100	2.27359400
C	1.90937400	-0.20696100	2.46073500
H	2.05442400	-0.13865800	3.55098900
H	1.89404300	0.81867000	2.06812000
H	2.77019700	-0.73437400	2.03456000
C	-0.98316400	1.00387600	2.86902000
H	-1.97980800	1.35427900	2.56906700
H	-0.23510900	1.72521300	2.51272500
H	-0.94205100	0.99608700	3.97059700
C	-3.13018900	-1.35080300	2.39806500
H	-3.34451300	-1.81773700	3.37234600
H	-3.70785700	-1.88797800	1.63343000
H	-3.48777500	-0.31536700	2.44035700
C	-1.49475500	-3.89843500	1.35820100
H	-1.03285800	-4.31323000	0.45080900
H	-2.58358500	-3.87574500	1.22675000
H	-1.26936200	-4.59070400	2.18588200
C	-1.55755400	0.45361500	-2.46093500
H	-1.66540500	-0.46100400	-3.06530300
H	-2.07990800	1.27060800	-2.97996400
C	-0.08685700	0.75945900	-2.22865200
H	0.46040400	0.62338200	-3.17382600
H	0.03046200	1.82325300	-1.96444900
C	-2.98940600	1.55386400	-0.08922600
C	-2.21064100	2.71928300	-0.13111100
H	-1.27879100	2.74836000	-0.69755200
C	-2.61011900	3.85821500	0.56397500
H	-1.99571000	4.76009600	0.51867900
C	-3.78662900	3.84574700	1.31355000
H	-4.09683100	4.73819200	1.86153600
C	-4.56794700	2.69175000	1.35629100
H	-5.49395000	2.67625600	1.93505900
C	-4.17389600	1.55052000	0.65871100
H	-4.79757100	0.65553500	0.70300800
C	-3.79293700	-0.99013900	-1.30809300
C	-3.69083500	-2.38025500	-1.18742700
H	-2.77873600	-2.82296600	-0.78209700
C	-4.74472200	-3.19994700	-1.58886200
H	-4.65795800	-4.28391000	-1.48801600
C	-5.90341700	-2.63355800	-2.11818800
H	-6.73135400	-3.27407600	-2.43045400
C	-6.00462200	-1.24798800	-2.25513800
H	-6.90775200	-0.80346800	-2.67880000
C	-4.95356300	-0.42575900	-1.85564200
H	-5.04311200	0.65703100	-1.97236800
C	1.85960100	-0.20507500	-1.17406500
C	3.80169400	-1.46299200	-0.68636500

C	4.60371500	-0.57094100	0.04893100
H	4.16268500	0.32420300	0.49156500
C	5.96254300	-0.82202500	0.23657600
H	6.55550400	-0.11034800	0.81828200
C	6.58047200	-1.95797000	-0.29644000
C	5.77584200	-2.85180900	-1.01776800
H	6.22370000	-3.75590600	-1.44094800
C	4.41796500	-2.61710700	-1.20157900
H	3.80294200	-3.32974600	-1.75621700
C	8.05303300	-2.21598500	-0.11673900
H	8.50193800	-1.51009800	0.59664000
H	8.59262700	-2.11381700	-1.07257300
H	8.24002400	-3.23695300	0.25058700
C	2.42682500	2.22531600	-1.05672200
C	1.49765700	2.56781300	-0.06607200
H	0.82406200	1.81129200	0.33721100
C	1.41847100	3.87804700	0.40560900
H	0.68094900	4.11165800	1.17909600
C	2.25610600	4.88804800	-0.07436400
C	3.18907500	4.53076600	-1.05841700
H	3.86650000	5.29068700	-1.45826800
C	3.27542100	3.23157000	-1.54475800
H	4.01171500	2.98468400	-2.31419600
C	2.16618100	6.30350700	0.43071000
H	1.44325000	6.39157600	1.25440000
H	1.84955800	6.99282300	-0.36857800
H	3.14160900	6.66080600	0.79623100
H	3.50494900	0.72859900	-1.91661900

I^{Rh}·H_2O

P	-2.36719300	0.04022100	-0.81255900
N	0.47278100	0.12828700	-1.06475100
N	2.40120500	-1.10143700	-0.79339800
N	2.57425900	1.14935000	-1.46178300
C	0.60250200	-2.02018700	1.89086100
C	0.57729500	-0.68726400	2.33167100
C	-0.82235700	-0.28234400	2.45115800
C	-1.64739400	-1.42946200	2.16861900
C	-0.77972200	-2.46615100	1.72891800
C	1.78600400	-2.90810500	1.72313700
H	2.72741700	-2.34709100	1.74702600
H	1.73512600	-3.47109700	0.78283800
H	1.79909400	-3.63591500	2.55132700
C	1.75261100	0.16218300	2.67313800
H	1.93865100	0.11280800	3.75827700
H	1.58403400	1.21640900	2.41737400
H	2.65769100	-0.18331800	2.15834000
C	-1.28401700	1.03148200	2.98232400
H	-2.32237800	1.24477900	2.69775400
H	-0.64716500	1.85243700	2.62459600
H	-1.22726900	1.02079000	4.08322200
C	-3.12559700	-1.52747800	2.35650600
H	-3.33672400	-1.92973800	3.35993000
H	-3.58925200	-2.19773200	1.62080400
H	-3.60768100	-0.54511200	2.28540100
C	-1.14280000	-3.84565900	1.29953100
H	-0.63258000	-4.09592900	0.35643400
H	-2.22596700	-3.96888500	1.17610800

H	-0.80303900	-4.56463800	2.06265900
C	-1.56042300	0.56338500	-2.37372800
H	-1.56381700	-0.34597000	-2.99532600
H	-2.13828700	1.34032400	-2.89533000
C	-0.13062100	0.99432600	-2.08607400
H	0.45452100	0.94266900	-3.01689100
H	-0.11896100	2.05429800	-1.78320100
C	-3.15006400	1.48848500	-0.03122300
C	-2.44348300	2.69935300	0.00912500
H	-1.47880700	2.79910900	-0.49131600
C	-2.95856700	3.79249700	0.70115300
H	-2.40035800	4.73104500	0.71910100
C	-4.17872100	3.68770200	1.36951600
H	-4.58007200	4.54453700	1.91514300
C	-4.88542600	2.48666000	1.33572300
H	-5.84262000	2.39752700	1.85392400
C	-4.37612000	1.39111800	0.63900700
H	-4.94312200	0.45829500	0.62466300
C	-3.69019600	-1.09715600	-1.31766100
C	-3.46526600	-2.47361700	-1.21126800
H	-2.53147900	-2.83774700	-0.77896600
C	-4.42592500	-3.37889400	-1.65986200
H	-4.24358000	-4.45190400	-1.56979600
C	-5.61298700	-2.91102300	-2.22159000
H	-6.36852300	-3.61867000	-2.57025600
C	-5.83478600	-1.53823300	-2.34604700
H	-6.75993800	-1.17095300	-2.79539700
C	-4.87653600	-0.63046500	-1.90096700
H	-5.05828400	0.44170800	-2.00855500
C	1.84827200	0.03913300	-1.07322700
C	3.77368700	-1.27812600	-0.58541300
C	4.59844300	-0.38883100	0.13138500
H	4.18719600	0.53810600	0.53589300
C	5.94155000	-0.68392100	0.34681500
H	6.55509900	0.02544900	0.91024000
C	6.52354600	-1.86454800	-0.13467300
C	5.69593000	-2.75361000	-0.82974200
H	6.11090600	-3.69249300	-1.20732900
C	4.34887100	-2.47360600	-1.04433400
H	3.70766300	-3.19067000	-1.56183700
C	7.98203700	-2.15634300	0.09848700
H	8.62136100	-1.39462900	-0.37592200
H	8.26645100	-3.13600000	-0.31122500
H	8.22380400	-2.15589200	1.17316000
C	2.37214100	2.48435000	-1.07410500
C	1.49053600	2.85832800	-0.05246000
H	0.90797200	2.09755600	0.46855400
C	1.34382000	4.20071400	0.29355500
H	0.64692800	4.46344100	1.09457600
C	2.06958100	5.21000700	-0.34639400
C	2.96090200	4.81984400	-1.35558300
H	3.55046200	5.57990800	-1.87594600
C	3.10988600	3.48630300	-1.72033000
H	3.80547300	3.21015600	-2.51716800
C	1.89672600	6.66072200	0.01660600
H	1.30840100	6.77662100	0.93818000
H	1.37579500	7.21073800	-0.78411700
H	2.86834600	7.15551500	0.16779000
H	3.50682900	0.95068200	-1.81152100

O	1.02753200	-3.48477700	-1.41351800
H	1.44309400	-2.61762300	-1.18491200
H	0.99001600	-3.47510000	-2.37552600
Rh	-0.55783300	-0.74812700	0.41039900

I$^{\mathbf{Rh}}$·MeOH

P	-2.35868900	0.00506400	-0.78114900
N	0.47778400	0.12106400	-1.01188800
N	2.40944100	-1.08958400	-0.69647300
N	2.57917400	1.13948200	-1.43319300
C	0.63956600	-1.68044800	2.13116600
C	0.58527400	-0.31137300	2.44380200
C	-0.82383400	0.06932800	2.53528000
C	-1.62157700	-1.11833800	2.37049500
C	-0.73030000	-2.17375900	2.03344300
C	1.84472600	-2.54938200	2.03527500
H	2.76639100	-1.96616600	1.92652000
H	1.76694900	-3.25015600	1.19426300
H	1.92341400	-3.14358700	2.96089900
C	1.75185800	0.57999500	2.69713000
H	2.07056300	0.47329400	3.74668900
H	1.51091900	1.63695500	2.53007800
H	2.60467600	0.30824000	2.06197600
C	-1.32276400	1.41978600	2.92133400
H	-2.36437300	1.57230800	2.60955300
H	-0.70910400	2.21510800	2.47620200
H	-1.27245300	1.53271800	4.01661900
C	-3.09684800	-1.23554000	2.57466900
H	-3.29213800	-1.60364300	3.59421100
H	-3.55550400	-1.94119800	1.86905400
H	-3.59645500	-0.26471100	2.47215300
C	-1.05892100	-3.59738500	1.74410200
H	-0.57531600	-3.91401200	0.80636900
H	-2.14054500	-3.76826700	1.68281800
H	-0.65962700	-4.23342500	2.55070500
C	-1.54560000	0.44766000	-2.36489200
H	-1.52648800	-0.48985500	-2.94198600
H	-2.13062500	1.18730100	-2.93058400
C	-0.12758200	0.92054900	-2.08629300
H	0.46892300	0.83319200	-3.00703100
H	-0.13714800	1.99407000	-1.83483200
C	-3.22527800	1.47180900	-0.13587700
C	-2.57024300	2.71162700	-0.16176800
H	-1.59549200	2.81666700	-0.64253500
C	-3.15099400	3.82606200	0.43781500
H	-2.63266100	4.78690100	0.40708100
C	-4.38607900	3.71362000	1.07747000
H	-4.83897900	4.58706700	1.55169200
C	-5.04260100	2.48408600	1.10585400
H	-6.01162700	2.39019900	1.60080200
C	-4.46742600	1.36616000	0.50166100
H	-4.99402300	0.41010700	0.53362100
C	-3.62702600	-1.21712600	-1.22325200
C	-3.42908200	-2.56288700	-0.89984400
H	-2.54590500	-2.86077200	-0.33303400
C	-4.35074200	-3.52588900	-1.30973600
H	-4.18924700	-4.57491900	-1.05266000
C	-5.47057600	-3.14678600	-2.04783300

H	-6.19315100	-3.89995000	-2.36986800
C	-5.66801900	-1.80520100	-2.38153200
H	-6.54228200	-1.50869900	-2.96505500
C	-4.74967700	-0.84079500	-1.97454600
H	-4.91163400	0.20656900	-2.24149600
C	1.85664900	0.03968500	-1.01438100
C	3.78446200	-1.26688100	-0.50102800
C	4.62195700	-0.36195900	0.17950600
H	4.22164600	0.58104100	0.55703800
C	5.96507800	-0.66005000	0.39016100
H	6.58966400	0.06091800	0.92602900
C	6.53326500	-1.85871100	-0.06238900
C	5.69339400	-2.76055900	-0.72560100
H	6.09891500	-3.71152000	-1.08250700
C	4.34593400	-2.47768600	-0.93445300
H	3.69584500	-3.20162900	-1.43133800
C	7.99072200	-2.15611400	0.16981500
H	8.63258200	-1.39105900	-0.29531800
H	8.27354600	-3.13265800	-0.24823500
H	8.22988000	-2.16681600	1.24516600
C	2.36099200	2.49217800	-1.12383400
C	1.43677700	2.92499000	-0.16516300
H	0.82836900	2.19769700	0.37264000
C	1.28435100	4.28526400	0.10119200
H	0.55149900	4.59520000	0.85173400
C	2.04635400	5.25546300	-0.55599300
C	2.97870900	4.80722800	-1.50230200
H	3.59706200	5.53498200	-2.03531900
C	3.13364400	3.45575000	-1.78828900
H	3.86263900	3.13374300	-2.53655200
C	1.88101700	6.72391400	-0.26819300
H	1.13210700	6.89749700	0.51771500
H	1.56010600	7.27268300	-1.16800800
H	2.82932200	7.17540600	0.06338300
H	3.51533400	0.93408500	-1.76948800
O	0.98314600	-3.46268100	-1.11701700
H	1.43307500	-2.60680800	-0.91508800
Rh	-0.54468300	-0.58456000	0.54964100
C	0.12732100	-3.24871500	-2.20021100
H	-0.28078600	-4.21682400	-2.53216700
H	-0.73323900	-2.59728100	-1.95116600
H	0.64519700	-2.79349200	-3.06688700

II^{Ir}

P	2.24742400	0.33311100	0.87491600
N	-0.63735000	0.56508400	0.74793700
N	-2.17498100	-1.12873400	0.87048500
N	-2.82091600	1.00919900	1.53607000
C	-0.18747500	-1.88649400	-2.14305300
C	-0.63291700	-0.55911600	-2.36868000
C	0.52766200	0.30121900	-2.52232400
C	1.69362000	-0.54285400	-2.45226200
C	1.26752300	-1.88418200	-2.16054700
C	-1.02091900	-3.09457600	-1.87400100
H	-2.08657800	-2.84355600	-1.79325100
H	-0.70264300	-3.56481700	-0.93086100
H	-0.89967700	-3.83145500	-2.68283700
C	-2.04993200	-0.10627600	-2.47640800

H	-2.34092600	-0.06647600	-3.53819700
H	-2.18305700	0.90058100	-2.06229700
H	-2.73644800	-0.79053200	-1.96296000
C	0.48325800	1.73963500	-2.92963200
H	1.42722400	2.25029800	-2.69919000
H	-0.32852500	2.27415700	-2.41536500
H	0.30636300	1.82888900	-4.01374800
C	3.09712200	-0.13316300	-2.75229600
H	3.31164000	-0.36093700	-3.80881900
H	3.82193100	-0.68189100	-2.13658300
H	3.25491800	0.94142600	-2.59984600
C	2.12089800	-3.10783100	-2.07958100
H	1.75402700	-3.78820100	-1.29846800
H	3.16615100	-2.85608300	-1.85629800
H	2.10315400	-3.65180300	-3.03781400
C	1.25898400	1.34755500	2.05314600
H	1.05435300	0.70400100	2.92295100
H	1.82120500	2.22746400	2.39654400
C	-0.05779900	1.73943800	1.38647100
H	-0.73018600	2.15282900	2.15102100
H	0.10834000	2.53328300	0.63922800
C	3.45680100	1.46421500	0.11316800
C	2.99854900	2.67018500	-0.43437900
H	1.94213700	2.94026500	-0.36639700
C	3.88175900	3.52630500	-1.08649100
H	3.51514100	4.46497100	-1.50766200
C	5.22918000	3.18097700	-1.20632300
H	5.92144200	3.84975300	-1.72266800
C	5.68975700	1.98310300	-0.66267300
H	6.74354200	1.70997900	-0.75123100
C	4.80895200	1.12675100	-0.00154600
H	5.18317700	0.19043900	0.41821600
C	3.24493900	-0.80290700	1.88764700
C	3.46819800	-2.11115700	1.44360400
H	3.01021600	-2.45469700	0.51384700
C	4.26109700	-2.97849800	2.19526300
H	4.42924300	-3.99935400	1.84560400
C	4.83124800	-2.54365900	3.39050200
H	5.44969200	-3.22424600	3.98013400
C	4.61181700	-1.23877800	3.83691300
H	5.05937600	-0.89660200	4.77257800
C	3.82334800	-0.36862900	3.08861400
H	3.66675100	0.65448400	3.43997400
C	-1.86047100	0.17490900	1.03648900
C	-3.43304300	-1.71795900	0.67975600
C	-4.52412300	-1.05386500	0.10433200
H	-4.44277000	-0.01060700	-0.20355200
C	-5.72596800	-1.73133900	-0.10068600
H	-6.56216700	-1.19123700	-0.55312500
C	-5.88083200	-3.07880100	0.23860700
C	-4.77582100	-3.73419600	0.79989700
H	-4.85695300	-4.79027400	1.07232400
C	-3.57529100	-3.07066200	1.02302700
H	-2.72737200	-3.60081800	1.46348500
C	-7.18274000	-3.80321300	0.02342300
H	-7.87312500	-3.21296000	-0.59579600
H	-7.68615800	-4.00741800	0.98246800
H	-7.02482800	-4.77366800	-0.47161600
C	-3.09695300	2.33958800	1.16357500

C	-2.56482300	2.93531100	0.01647400
H	-1.88649400	2.37812000	-0.62960200
C	-2.90396200	4.24754800	-0.31092900
H	-2.47480700	4.69070500	-1.21386900
C	-3.77925200	5.00040700	0.47673500
C	-4.31248400	4.38413700	1.61773400
H	-5.00468300	4.93989500	2.25642900
C	-3.97754300	3.08010100	1.96311600
H	-4.40238200	2.62326200	2.86071400
C	-4.13484300	6.42098400	0.12849500
H	-3.73357300	6.70696900	-0.85414800
H	-3.73082100	7.12535000	0.87352000
H	-5.22588300	6.56653200	0.10588700
H	-3.55349600	0.55359100	2.07196700
H	-1.32353800	-1.74351000	0.92055600
O	0.27423700	-2.13721400	0.85386800
H	0.80457500	-2.10168800	1.65612800
Ir	0.64577700	-0.60347400	-0.52343600

II^{Rh}

Rh	0.70377800	-0.65491000	-0.57190300
P	2.31414800	0.27474100	0.81111400
N	-0.57466000	0.52802200	0.65937800
N	-2.08948200	-1.18255100	0.75164700
N	-2.76045900	0.93116100	1.47347400
C	-0.10640800	-1.91112400	-2.19236800
C	-0.56809300	-0.58502000	-2.38599300
C	0.58397100	0.28428900	-2.52878700
C	1.75638500	-0.54442100	-2.49719100
C	1.34439100	-1.88940200	-2.21973500
C	-0.91967500	-3.13586900	-1.94586000
H	-1.99319000	-2.91195700	-1.89767200
H	-0.61663100	-3.59698500	-0.99292200
H	-0.75464300	-3.87139900	-2.74812200
C	-1.98719300	-0.13612000	-2.48243400
H	-2.22858000	0.08776900	-3.53378900
H	-2.16223400	0.78069200	-1.90505400
H	-2.68363000	-0.90627500	-2.12961000
C	0.52148000	1.73716500	-2.87356900
H	1.46634500	2.24654800	-2.64395100
H	-0.28487000	2.24249000	-2.32366500
H	0.32095800	1.86869900	-3.94930600
C	3.15067200	-0.11554500	-2.80758900
H	3.35013600	-0.32356700	-3.87135200
H	3.89128700	-0.66856600	-2.21507100
H	3.30119200	0.95806000	-2.64072300
C	2.21094300	-3.10271000	-2.14554400
H	1.85150700	-3.79153300	-1.36849300
H	3.25479500	-2.84289600	-1.92584800
H	2.19432300	-3.64137200	-3.10693600
C	1.31424700	1.28362300	1.98598900
H	1.09678900	0.63027700	2.84539800
H	1.87952800	2.15466200	2.34746500
C	0.00652200	1.69153400	1.31087800
H	-0.66799500	2.10921600	2.07215300
H	0.18734500	2.48803500	0.57005200
C	3.52992900	1.41506300	0.07148700
C	3.09281600	2.65084800	-0.42533800

H	2.04696400	2.94970700	-0.32693300
C	3.98613700	3.50863300	-1.06217000
H	3.63503300	4.47030600	-1.44270200
C	5.32292800	3.13783200	-1.21622700
H	6.02256400	3.80893900	-1.71939400
C	5.76360400	1.91151500	-0.72143400
H	6.80957200	1.61825100	-0.83477200
C	4.87301600	1.05240700	-0.07797800
H	5.23289500	0.09430800	0.30342100
C	3.30860400	-0.86373600	1.82194100
C	3.52483500	-2.17170500	1.37421700
H	3.05858000	-2.51108500	0.44745900
C	4.31929400	-3.04268100	2.12011100
H	4.48309500	-4.06326800	1.76760800
C	4.89595800	-2.61190300	3.31372200
H	5.51549800	-3.29539400	3.89887200
C	4.68196500	-1.30750300	3.76450000
H	5.13467400	-0.96887800	4.69898600
C	3.89320900	-0.43321600	3.02140000
H	3.74119400	0.58987200	3.37497800
C	-1.78941800	0.12123300	0.94800600
C	-3.34557000	-1.78111200	0.58116900
C	-4.44593800	-1.12479400	0.01416100
H	-4.36753900	-0.08405000	-0.30376700
C	-5.64950400	-1.80575600	-0.16721700
H	-6.49363900	-1.27143100	-0.61179200
C	-5.79619600	-3.15074400	0.18618100
C	-4.68132400	-3.79955700	0.73545300
H	-4.75627700	-4.85357000	1.01763400
C	-3.47918000	-3.13160100	0.93640000
H	-2.62407300	-3.65587800	1.36987400
C	-7.10079400	-3.87880500	0.00087900
H	-7.79929900	-3.30019900	-0.62020000
H	-7.59163400	-4.06434300	0.97024800
H	-6.94937000	-4.85856400	-0.47747300
C	-3.07557400	2.25951100	1.13046000
C	-2.52583200	2.91857300	0.02692200
H	-1.79670400	2.41563800	-0.60734000
C	-2.91169500	4.22501500	-0.27149200
H	-2.46754500	4.71669300	-1.14148100
C	-3.84992600	4.91193100	0.50368400
C	-4.39927100	4.23345400	1.60078900
H	-5.14126600	4.73545600	2.22796700
C	-4.02073000	2.93389800	1.91581200
H	-4.46295300	2.42768800	2.77805700
C	-4.25097500	6.32867100	0.19098200
H	-3.88098400	6.64395900	-0.79506100
H	-3.84630700	7.02989000	0.93894300
H	-5.34536800	6.44629500	0.19766500
H	-3.48449400	0.44883900	1.99732100
H	-1.22830200	-1.79029500	0.78927100
O	0.35945800	-2.20918800	0.76049700
H	0.80189800	-2.09511900	1.60822100

III^{Ir}

P	-2.27878800	0.35887800	0.88314900
N	0.60278000	0.49408700	0.91534000
N	2.27431500	-1.09642900	0.87963800

N	2.78207100	1.03980700	1.63133100
C	0.56725200	-1.46999200	-2.21642300
C	-0.53283200	-2.45064600	-2.06594400
C	-1.74530600	-1.60682900	-1.99590600
C	-1.42610300	-0.30912800	-2.51570400
C	0.02906800	-0.24246700	-2.64988400
C	2.00609900	-1.86887900	-2.26558000
H	2.24468300	-2.61798200	-1.49871500
H	2.67949600	-1.01405400	-2.12448200
H	2.23556300	-2.31899800	-3.24582200
C	-0.42867100	-3.78482200	-1.87947000
C	-3.13130400	-2.16103500	-1.87843900
H	-3.48198600	-2.53404200	-2.85513400
H	-3.84186400	-1.39732100	-1.53272800
H	-3.16856800	-3.00067900	-1.17057500
C	-2.38299700	0.70232500	-3.05654900
H	-2.01734700	1.72712700	-2.90394000
H	-3.36953900	0.61746200	-2.58290600
H	-2.51295600	0.54618000	-4.14009800
C	0.78338300	0.92479600	-3.19443200
H	0.78123800	0.88577800	-4.29624100
H	1.82945100	0.92065800	-2.86091800
H	0.32138800	1.87565900	-2.89473000
C	-3.27967400	-0.82367900	1.84653600
C	-3.09160100	-2.19926400	1.68611700
H	-2.36702300	-2.56310200	0.95339300
C	-3.82363600	-3.10572400	2.45401300
H	-3.67126200	-4.17891500	2.31972500
C	-4.74716600	-2.63908900	3.38737000
H	-5.32193300	-3.34724600	3.98858500
C	-4.93808400	-1.26555300	3.55532300
H	-5.66051000	-0.89831400	4.28752500
C	-4.20854700	-0.36025100	2.78932600
H	-4.36717900	0.71346400	2.92294800
C	-3.48700600	1.49356800	0.12172800
C	-4.77686200	1.06493000	-0.21137100
H	-5.11558200	0.06452600	0.06788300
C	-5.63980900	1.91322000	-0.90543000
H	-6.64475900	1.57033600	-1.16145700
C	-5.22255100	3.19256100	-1.26972400
H	-5.89988600	3.85555500	-1.81269400
C	-3.93793700	3.62537400	-0.93724400
H	-3.60643800	4.62709400	-1.21966000
C	-3.07158300	2.77920500	-0.24953300
H	-2.06141500	3.12306000	-0.01391800
C	-1.34311100	1.31669500	2.15186500
H	-1.88910800	2.21509100	2.47311000
H	-1.23686500	0.65217500	3.02288200
C	0.03546700	1.66200800	1.59507700
H	-0.03700900	2.50751200	0.89150000
H	0.68240700	1.98857100	2.42204300
C	1.86114600	0.16582600	1.12969300
C	3.59676700	-1.54239400	0.67204100
C	3.92044300	-2.84806800	1.05699300
H	3.15939300	-3.46825100	1.53526500
C	5.19122700	-3.35915700	0.80791300
H	5.41762400	-4.38556500	1.10910500
C	6.18002300	-2.58850200	0.18472300
C	5.83865800	-1.28565700	-0.19912700

H	6.58008600	-0.66204700	-0.70601500
C	4.56719600	-0.76559200	0.02618200
H	4.33094600	0.24065300	-0.32430300
C	7.56400800	-3.13232300	-0.04724600
H	8.03499500	-2.67195800	-0.92800600
H	7.54813300	-4.22191400	-0.19549300
H	8.21672000	-2.92804700	0.81765400
C	2.98170500	2.37090300	1.20485200
C	3.75866700	3.21833200	2.00262100
H	4.16554500	2.84670700	2.94612000
C	4.01427400	4.52288700	1.59501200
H	4.62864700	5.16651400	2.23063700
C	3.49721700	5.02939200	0.39451300
C	2.72470900	4.16799000	-0.39088300
H	2.31606500	4.52563100	-1.33994200
C	2.47069700	2.85290000	-0.00396900
H	1.87453600	2.20684900	-0.65144800
C	3.75477100	6.45410300	-0.01694100
H	3.15152100	7.15336800	0.58495200
H	3.50164600	6.62040900	-1.07366900
H	4.81027800	6.72848300	0.13012100
H	3.56971800	0.61979600	2.11687500
H	-1.31910700	-4.40740000	-1.76168900
H	0.54165200	-4.28695100	-1.91287400
H	1.55511700	-1.83377200	0.91403800
O	0.56613900	-3.38620300	1.09296800
H	0.15289200	-3.49099000	0.21717000
H	-0.17797100	-3.24868900	1.69300400
Ir	-0.66611500	-0.41254500	-0.55434800

III^{Rh}

Rh	-0.72119300	-0.41095400	-0.64387200
P	-2.32907700	0.29383300	0.82027600
N	0.55377200	0.46358900	0.83566200
N	2.19138000	-1.14859900	0.80016600
N	2.73800000	0.97406000	1.57615800
C	0.48959000	-1.40343000	-2.32310700
C	-0.66479900	-2.31833300	-2.18118000
C	-1.84216500	-1.42892200	-2.17119700
C	-1.43407400	-0.13561800	-2.60795600
C	0.02358600	-0.13555700	-2.70448300
C	1.90399200	-1.87921200	-2.35162800
H	2.09017100	-2.64448100	-1.58624300
H	2.62129200	-1.06314300	-2.19848100
H	2.12043600	-2.33691200	-3.33130900
C	-0.62939500	-3.65228200	-1.95704600
C	-3.25493800	-1.91863300	-2.11463500
H	-3.56438400	-2.29999000	-3.10193200
H	-3.95033300	-1.11588900	-1.83275500
H	-3.37172400	-2.73863100	-1.39293600
C	-2.31926100	0.97260600	-3.07484400
H	-1.90802000	1.95623400	-2.80901500
H	-3.32682300	0.89488400	-2.64588900
H	-2.41426800	0.93323200	-4.17253500
C	0.84609500	1.01863500	-3.17371400
H	0.86564800	1.03794100	-4.27585900
H	1.88358600	0.94575900	-2.82179700
H	0.42616900	1.97541400	-2.83350200

C	-3.25325000	-0.96758300	1.76100200
C	-3.07990800	-2.32251800	1.46167500
H	-2.41707900	-2.61301600	0.64248300
C	-3.74899400	-3.30048300	2.19897700
H	-3.60735300	-4.35608900	1.95674900
C	-4.59701300	-2.92629200	3.23968200
H	-5.12189100	-3.68985500	3.81826200
C	-4.77657700	-1.57478100	3.54335800
H	-5.44079100	-1.28056000	4.35896300
C	-4.10939500	-0.59825800	2.80817700
H	-4.25841500	0.45768900	3.05005500
C	-3.60284100	1.41196500	0.14775600
C	-4.88762400	0.94889500	-0.15930500
H	-5.18109100	-0.07284100	0.09278300
C	-5.80417400	1.79153300	-0.78882100
H	-6.80481100	1.42208400	-1.02414100
C	-5.44700000	3.09979000	-1.11277900
H	-6.16691100	3.75828400	-1.60394200
C	-4.16836800	3.56711300	-0.80516000
H	-3.88374100	4.59149500	-1.05547600
C	-3.24827000	2.72659800	-0.18344400
H	-2.24341100	3.09853000	0.03222900
C	-1.38514000	1.24499200	2.09149400
H	-1.94107400	2.12749800	2.43949000
H	-1.25061600	0.56682300	2.94810700
C	-0.02193900	1.62233200	1.51565400
H	-0.12649400	2.46308100	0.80920400
H	0.62650300	1.97018800	2.33383400
C	1.80045800	0.12362200	1.05858200
C	3.49832400	-1.62599600	0.58530100
C	3.77996900	-2.95263400	0.93228500
H	2.99719300	-3.56124800	1.39059300
C	5.03479900	-3.49634900	0.67314000
H	5.22678200	-4.53793500	0.94486500
C	6.05083400	-2.73958900	0.07680600
C	5.75243800	-1.41587600	-0.26889000
H	6.51596900	-0.80166100	-0.75407800
C	4.49683300	-0.86191100	-0.03308700
H	4.29335600	0.16106100	-0.35466100
C	7.41798100	-3.32038000	-0.16583900
H	7.91298600	-2.84011500	-1.02247800
H	7.36753200	-4.40188000	-0.35935100
H	8.06884400	-3.17482800	0.71220900
C	2.95695300	2.31480000	1.19702800
C	3.76495400	3.11468700	2.01389000
H	4.18056400	2.69652200	2.93413500
C	4.04163100	4.42912400	1.65487500
H	4.67983900	5.03334700	2.30553800
C	3.51669900	4.99461900	0.48450000
C	2.71425000	4.18089300	-0.32122000
H	2.29792900	4.58429000	-1.24848200
C	2.43730600	2.85711600	0.01752300
H	1.81763500	2.25030800	-0.64487900
C	3.79573800	6.42989500	0.12657800
H	3.20185300	7.11512900	0.75352800
H	3.54553000	6.63926000	-0.92329800
H	4.85497700	6.68335500	0.28377600
H	3.51276700	0.53352000	2.06345200
H	-1.55099700	-4.23020300	-1.85190200

H	0.31679900	-4.19938700	-1.94846800
H	1.45314700	-1.86351200	0.85694600
O	0.35358200	-3.32792800	1.09586500
H	-0.07607000	-3.43935100	0.23080900
H	-0.37587600	-3.19634500	1.71497500

IV^{Ir}

P	2.26410900	0.36239100	0.82060700
N	-0.64555800	0.52480500	0.77170300
N	-2.25726800	-1.10621900	0.74238200
N	-2.83536900	1.01860600	1.50736500
C	-0.20064000	-1.95575200	-2.11200200
C	-0.65939500	-0.63516600	-2.34520600
C	0.49362200	0.23276000	-2.51329000
C	1.66709100	-0.59925500	-2.44392200
C	1.25443800	-1.94008100	-2.13146300
C	-1.01528300	-3.17105300	-1.82502700
H	-2.09070300	-2.95094400	-1.81709900
H	-0.73385800	-3.57442400	-0.83892800
H	-0.82487600	-3.94761900	-2.58125200
C	-2.07604300	-0.18580000	-2.47339800
H	-2.29998800	0.00914400	-3.53425900
H	-2.25592500	0.74822500	-1.92542400
H	-2.78085400	-0.94284200	-2.11022700
C	0.42390200	1.65927000	-2.95513500
H	1.37771300	2.17685800	-2.79618400
H	-0.35936200	2.20715700	-2.41225900
H	0.18557900	1.71365300	-4.02981400
C	3.06324200	-0.18487900	-2.77122600
H	3.27522500	-0.46449300	-3.81573200
H	3.80072300	-0.68938900	-2.13310800
H	3.20531700	0.89848000	-2.67716500
C	2.11598400	-3.15848600	-2.05836300
H	1.79044200	-3.82090000	-1.24414700
H	3.17015200	-2.89801400	-1.89494000
H	2.05496800	-3.72548200	-3.00139700
C	1.28544000	1.30458100	2.06271400
H	1.14644800	0.63183700	2.92255800
H	1.82705400	2.19751500	2.40551900
C	-0.07100200	1.67316100	1.46351000
H	-0.73057200	2.01256300	2.27492300
H	0.03502400	2.52147400	0.76777400
C	3.33681800	1.58443300	-0.01555800
C	2.76555000	2.74484000	-0.55517600
H	1.69593600	2.93624300	-0.45110500
C	3.55099900	3.66107800	-1.24981300
H	3.09234000	4.56127100	-1.66487800
C	4.91578400	3.42482500	-1.42140200
H	5.53078900	4.14036800	-1.97178700
C	5.49103500	2.27441400	-0.88515700
H	6.55879500	2.08348100	-1.01311300
C	4.70770700	1.35879400	-0.18257000
H	5.17479600	0.46159600	0.22801900
C	3.43968500	-0.67796300	1.73710600
C	3.76860800	-1.94174600	1.23701600
H	3.27505300	-2.31217700	0.33644300
C	4.70066500	-2.73581800	1.90271200
H	4.95139700	-3.72370000	1.51040900

C	5.30229300	-2.27211300	3.07254200
H	6.02910100	-2.89640000	3.59731400
C	4.97447300	-1.01223100	3.57602000
H	5.44417900	-0.64844100	4.49251300
C	4.04872800	-0.21280300	2.90875700
H	3.80533400	0.77622800	3.30476000
C	-1.89491500	0.17167800	0.99023000
C	-3.54585200	-1.63416900	0.56523200
C	-4.60104000	-0.91307200	-0.00740900
H	-4.45607600	0.11935100	-0.32966800
C	-5.84311400	-1.51956800	-0.19024100
H	-6.65205800	-0.93657100	-0.63874600
C	-6.07108300	-2.85276100	0.16587100
C	-4.99953800	-3.56722400	0.71920900
H	-5.13958600	-4.61392900	1.00307000
C	-3.75976700	-2.97244100	0.92332700
H	-2.93991100	-3.54537500	1.36316900
C	-7.41680600	-3.50055400	-0.02074900
H	-8.06838500	-2.89609700	-0.66771600
H	-7.93156100	-3.62693800	0.94577900
H	-7.32085400	-4.50113800	-0.46894000
C	-3.06518100	2.36359500	1.15537600
C	-2.51196700	2.95391600	0.01593900
H	-1.85219100	2.38145400	-0.63662700
C	-2.80590900	4.28070500	-0.29685000
H	-2.36116200	4.71975700	-1.19419200
C	-3.65800200	5.05242000	0.49773200
C	-4.21187000	4.44185700	1.63215300
H	-4.88603500	5.01321900	2.27633100
C	-3.92015100	3.12397200	1.96372000
H	-4.35993900	2.67099100	2.85595000
C	-3.97127000	6.48571200	0.16095800
H	-3.55304600	6.76968100	-0.81515200
H	-3.55613100	7.17205700	0.91652500
H	-5.05785400	6.66020600	0.12923000
H	-3.60089600	0.56891900	2.00074500
H	-1.43620400	-1.75900800	0.74058800
O	0.17882700	-2.19206900	0.85398200
C	0.40112200	-2.20526500	2.22325400
H	-0.05635300	-1.34400700	2.75502800
H	-0.05038300	-3.11796600	2.65797400
H	1.47210400	-2.22265000	2.49664700
Ir	0.63352300	-0.64436000	-0.50292300

IV^{Rh}

Rh	-0.67720100	-0.69423900	0.55012000
P	-2.32459200	0.29571300	-0.75138100
N	0.58692200	0.49796700	-0.68736300
N	2.18026100	-1.14691300	-0.66917500
N	2.77407200	0.96593800	-1.45700600
C	0.15165300	-1.97581900	2.15555900
C	0.60299600	-0.65074900	2.37099700
C	-0.55489700	0.20606800	2.52920700
C	-1.72181100	-0.63096300	2.48423600
C	-1.29926200	-1.96627600	2.18235500
C	0.96995100	-3.19030800	1.88540800
H	2.04609300	-2.97551900	1.90999000
H	0.71202900	-3.58428600	0.88845300

H	0.75236500	-3.97267300	2.62820700
C	2.01559700	-0.19051600	2.49967700
H	2.22729500	0.03404200	3.55739100
H	2.19784500	0.73012900	1.92986300
H	2.72759400	-0.95408700	2.16537900
C	-0.49414200	1.64555100	2.92537800
H	-1.45312400	2.15202400	2.76192200
H	0.28183200	2.18212000	2.36143200
H	-0.24584300	1.73335400	3.99562600
C	-3.11638400	-0.22143800	2.81967200
H	-3.31043200	-0.48281400	3.87258400
H	-3.85946300	-0.74541800	2.20424700
H	-3.26913200	0.85884600	2.70851800
C	-2.15100000	-3.18972400	2.10413300
H	-1.82946100	-3.83809000	1.27705700
H	-3.21090400	-2.93980700	1.96391400
H	-2.06584900	-3.76838400	3.03826900
C	-1.34308000	1.24685600	-1.98608200
H	-1.18278000	0.57003200	-2.83903200
H	-1.89663300	2.12736400	-2.34252200
C	0.00182400	1.63989600	-1.37612900
H	0.65910200	1.99652700	-2.18274200
H	-0.12409300	2.48320400	-0.67691900
C	-3.41754900	1.51559300	0.05982000
C	-2.87165500	2.70703000	0.55668500
H	-1.80900000	2.92317300	0.43203100
C	-3.67442600	3.62521300	1.22905500
H	-3.23568500	4.55011800	1.60984600
C	-5.03148000	3.36099300	1.41924900
H	-5.66034500	4.07909900	1.95027300
C	-5.58194200	2.17990000	0.92474400
H	-6.64400800	1.96788500	1.06614000
C	-4.78140000	1.26134900	0.24636500
H	-5.22924800	0.34091400	-0.13331600
C	-3.47956300	-0.76216300	-1.67211300
C	-3.77448500	-2.03671600	-1.17863200
H	-3.26477900	-2.40084200	-0.28477400
C	-4.68951300	-2.84971000	-1.84540300
H	-4.91366800	-3.84605200	-1.45849500
C	-5.30654000	-2.39404500	-3.01023400
H	-6.01971900	-3.03301500	-3.53606000
C	-5.01107100	-1.12380000	-3.50819400
H	-5.49255400	-0.76706100	-4.42134100
C	-4.10304100	-0.30542300	-2.83971000
H	-3.88428700	0.69142500	-3.23077500
C	1.82745200	0.13432700	-0.92163900
C	3.46514200	-1.68572100	-0.49987400
C	4.53257000	-0.97101500	0.05877900
H	4.40091100	0.06569100	0.37282800
C	5.76942600	-1.58890900	0.23893600
H	6.58741300	-1.01036800	0.67678100
C	5.98103600	-2.92772500	-0.10586600
C	4.89848900	-3.63533000	-0.64638600
H	5.02552200	-4.68597600	-0.92184800
C	3.66376700	-3.02923100	-0.84805000
H	2.83557500	-3.59791100	-1.27755000
C	7.32099000	-3.58798100	0.07907500
H	7.98003400	-2.98763900	0.72234000
H	7.83211000	-3.72238100	-0.88832100

H	7.21682900	-4.58605100	0.53103400
C	3.02488100	2.31258300	-1.13002200
C	2.47316700	2.93846300	-0.00889700
H	1.79674800	2.39189200	0.64838100
C	2.79009500	4.26591900	0.27794200
H	2.34612000	4.73250000	1.16167400
C	3.66322300	5.00426500	-0.52544400
C	4.21561000	4.35837600	-1.64081200
H	4.90646500	4.90255600	-2.29087600
C	3.90233600	3.03919100	-1.94595300
H	4.34259300	2.55881400	-2.82359600
C	3.99856300	6.43947600	-0.21937800
H	3.59950500	6.74645600	0.75783800
H	3.57883000	7.11716800	-0.98036300
H	5.08733000	6.60202100	-0.20832200
H	3.52993400	0.50144100	-1.95132700
H	1.35261200	-1.79299800	-0.65203900
O	-0.25246000	-2.26410000	-0.77026000
C	-0.43463200	-2.26604100	-2.14371900
H	0.04011800	-1.40294200	-2.65793700
H	0.02353800	-3.17676000	-2.57850900
H	-1.49686000	-2.27747700	-2.45156600

MeOH

C	0.65175100	-0.01908400	0.00000000
H	1.09546500	0.98773400	-0.00000100
H	1.03140700	-0.54915900	-0.89441900
H	1.03140700	-0.54915700	0.89442000
O	-0.74260000	0.12302600	0.00000000
H	-1.12798600	-0.75912600	0.00000000

TS_I^{Ir}·MeOH-6·CH$_2$O

P	-2.34025500	0.36445700	-0.80301700
N	0.56555200	0.41640700	-0.91257600
N	2.25051400	-1.17319100	-0.89717100
N	2.73484200	0.98431400	-1.63534000
C	0.29546600	-1.89363500	2.07541200
C	0.72376700	-0.56381800	2.28770800
C	-0.45010400	0.27159100	2.47574900
C	-1.61209200	-0.60180400	2.47609200
C	-1.16494000	-1.91984900	2.17138800
C	1.13352500	-3.08854100	1.77192600
H	2.20005000	-2.87932500	1.92555000
H	1.00132600	-3.39678900	0.71826100
H	0.84692200	-3.93020500	2.42001000
C	2.12865200	-0.07251500	2.36922800
H	2.32884900	0.28587700	3.39104300
H	2.31007300	0.76697500	1.68647200
H	2.84667500	-0.86620900	2.13454000
C	-0.41362700	1.70382700	2.89486600
H	-1.36582100	2.20965400	2.69242300
H	0.38956000	2.24888900	2.37924100
H	-0.22030600	1.76905100	3.97805900
C	-3.00567300	-0.19985500	2.82925100
H	-3.16658700	-0.37551400	3.90480400
H	-3.75200900	-0.78611700	2.27654700
H	-3.18423800	0.86455000	2.63158900

C	-1.98534800	-3.16705500	2.12677700
H	-1.65708700	-3.83344100	1.31712000
H	-3.05275100	-2.94760200	1.99530300
H	-1.87380400	-3.71544600	3.07597000
C	-1.39899500	1.18575400	-2.15211200
H	-1.31404200	0.44871900	-2.96568100
H	-1.93263100	2.06701000	-2.53509400
C	-0.01399000	1.54902400	-1.63162300
H	0.61930500	1.83720300	-2.48245300
H	-0.07999600	2.43354100	-0.97634600
C	-3.38802600	1.63212800	-0.01852900
C	-2.78790600	2.80410900	0.46194100
H	-1.71371400	2.96346500	0.34446000
C	-3.55380200	3.77059700	1.10816600
H	-3.07768000	4.68210800	1.47590300
C	-4.92335600	3.57066000	1.29036800
H	-5.52313300	4.32734700	1.80100100
C	-5.52393500	2.40526000	0.81752500
H	-6.59525900	2.24456600	0.95607400
C	-4.76141000	1.43804800	0.16256900
H	-5.24445700	0.52992600	-0.20411200
C	-3.50134000	-0.77504400	-1.61548800
C	-3.79342800	-2.01132400	-1.03152200
H	-3.30034200	-2.30215900	-0.10265600
C	-4.70299700	-2.87636200	-1.63847500
H	-4.92425400	-3.84116500	-1.17730500
C	-5.32074100	-2.51031800	-2.83337100
H	-6.02935600	-3.18979200	-3.31218500
C	-5.03232600	-1.27709300	-3.42068900
H	-5.51521400	-0.98968200	-4.35710000
C	-4.12811800	-0.40853800	-2.81424500
H	-3.91446100	0.55731500	-3.27906900
C	1.83707900	0.07898000	-1.12230600
C	3.57744600	-1.55283500	-0.63875500
C	4.52221800	-0.73971600	0.01362300
H	4.26132100	0.27433000	0.31891100
C	5.79207500	-1.22652100	0.30794600
H	6.50134600	-0.56997300	0.82064200
C	6.17592400	-2.53444200	-0.01685800
C	5.22967800	-3.33831400	-0.66105600
H	5.48990900	-4.36634400	-0.92867900
C	3.95780700	-2.86245600	-0.97127100
H	3.23318800	-3.51063600	-1.46838600
C	7.55089700	-3.04458100	0.32453900
H	8.33254100	-2.47842600	-0.20710700
H	7.66369100	-4.10483400	0.05685600
H	7.75797500	-2.94504400	1.40190300
C	2.93899500	2.32214200	-1.25359300
C	2.32331400	2.90363300	-0.14105400
H	1.62658100	2.32517600	0.46585100
C	2.60172300	4.22626500	0.20322100
H	2.10855900	4.65618500	1.07957800
C	3.49773500	5.00575400	-0.53310400
C	4.11401800	4.40580200	-1.64072300
H	4.82585500	4.98168800	-2.23871200
C	3.84065600	3.09212300	-2.00169000
H	4.33365200	2.64814000	-2.87032500
C	3.78978500	6.43627000	-0.16673900
H	3.35512400	6.69818300	0.80841100

H	3.37603000	7.13120700	-0.91545600
H	4.87359500	6.62305200	-0.11792400
H	3.51864800	0.56440600	-2.12597400
O	0.80791600	-3.12151200	-1.44000900
H	1.53010700	-2.11598200	-1.16438500
C	-0.39917800	-2.64140600	-1.66571700
H	-1.21832400	-3.39012800	-1.66202500
H	-0.81136200	-1.90947200	-0.79290400
H	-0.52000700	-1.96847600	-2.54396200
Ir	-0.65683700	-0.58167500	0.50462300

TS_I^{Rh}·MeOH-5·CH_2O

P	-2.40172400	0.29418000	-0.75588900
N	0.51393000	0.35654900	-0.83623900
N	2.20047100	-1.22574500	-0.76803000
N	2.68340500	0.91135400	-1.57228200
C	0.20811600	-1.91290300	2.14145400
C	0.63746700	-0.57912700	2.32042400
C	-0.53455900	0.25790400	2.48233700
C	-1.69690300	-0.60757300	2.50184200
C	-1.24989200	-1.92958800	2.23029600
C	1.04467200	-3.11455500	1.87962200
H	2.10805200	-2.91143300	2.06092500
H	0.93821500	-3.42918200	0.82300000
H	0.73096900	-3.94865700	2.52481600
C	2.04220900	-0.09234200	2.41218200
H	2.23507900	0.25503000	3.43959900
H	2.22837500	0.75595300	1.74205100
H	2.76089900	-0.88465600	2.17569200
C	-0.49950900	1.70354400	2.84787800
H	-1.45749800	2.19801200	2.64696300
H	0.29437000	2.23590600	2.30577400
H	-0.28914100	1.80333700	3.92545500
C	-3.08939200	-0.19730700	2.84602300
H	-3.25394400	-0.37238900	3.92131100
H	-3.83786100	-0.78068200	2.29316500
H	-3.26226900	0.86792900	2.64897200
C	-2.07347300	-3.17407800	2.19977500
H	-1.75184100	-3.84878300	1.39441700
H	-3.14189200	-2.95538500	2.07765100
H	-1.95292300	-3.71485300	3.15243500
C	-1.44075800	1.09734300	-2.10196600
H	-1.34062400	0.34505700	-2.89980500
H	-1.97484900	1.96727600	-2.51018700
C	-0.06513200	1.47709200	-1.56969700
H	0.57276300	1.76636600	-2.41742400
H	-0.14648400	2.36798300	-0.92418100
C	-3.45332100	1.57760400	-0.00188400
C	-2.86356200	2.77078100	0.43865400
H	-1.79306300	2.94233200	0.30493900
C	-3.63510300	3.74561600	1.06590000
H	-3.16648200	4.67300800	1.40219400
C	-4.99979000	3.53431300	1.26907200
H	-5.60351300	4.29761000	1.76497800
C	-5.59058700	2.34871200	0.83534900
H	-6.65837900	2.17896300	0.98945200
C	-4.82295300	1.37279800	0.20011100
H	-5.29920100	0.44931600	-0.13585600

C	-3.56088800	-0.84847200	-1.56585300
C	-3.85945300	-2.07709100	-0.96931900
H	-3.37295200	-2.35779200	-0.03404300
C	-4.76812400	-2.94699700	-1.57092500
H	-4.99466600	-3.90562200	-1.09957400
C	-5.37802000	-2.59321200	-2.77341900
H	-6.08616200	-3.27610200	-3.24803100
C	-5.08265300	-1.36779600	-3.37375700
H	-5.55969400	-1.09021100	-4.31611200
C	-4.17946900	-0.49445800	-2.77280200
H	-3.96028400	0.46521500	-3.24772100
C	1.78272900	0.01903200	-1.03388300
C	3.53578900	-1.59676000	-0.54302600
C	4.48927600	-0.77502700	0.08550200
H	4.22327800	0.23423200	0.40306100
C	5.77561300	-1.24483800	0.33214600
H	6.49337200	-0.58117300	0.82342700
C	6.16682900	-2.54452600	-0.01658700
C	5.20971100	-3.35914100	-0.63056700
H	5.47564000	-4.38132700	-0.91452800
C	3.92128700	-2.89983600	-0.89412600
H	3.18841500	-3.55284700	-1.37256900
C	7.56288900	-3.03379500	0.26435000
H	8.31196700	-2.44908800	-0.29343700
H	7.68275100	-4.08936200	-0.01840500
H	7.81219500	-2.94057400	1.33329200
C	2.89784300	2.25310200	-1.21349900
C	2.28150400	2.85981200	-0.11482200
H	1.57607600	2.29663200	0.49596300
C	2.56966100	4.18549900	0.20893800
H	2.07542900	4.63428600	1.07523800
C	3.47669100	4.94444200	-0.53513800
C	4.09342900	4.32008900	-1.62905900
H	4.81362900	4.87953700	-2.23272100
C	3.81077700	3.00275400	-1.96924700
H	4.30475700	2.53975700	-2.82745000
C	3.78050500	6.37787800	-0.19002600
H	3.34342000	6.65930400	0.77864700
H	3.37680500	7.06523200	-0.95117900
H	4.86569900	6.55563300	-0.13842900
H	3.46616500	0.47866900	-2.05312000
O	0.75386700	-3.18838100	-1.24693600
H	1.48002300	-2.16519800	-0.99960800
C	-0.45404700	-2.72406400	-1.51733500
H	-1.26036800	-3.48711800	-1.54256700
H	-0.89563100	-2.00456600	-0.67073300
H	-0.55207200	-2.06904000	-2.41212300
Rh	-0.72161600	-0.65169500	0.55236800

TS_II^{Ir}-III^{Ir}

P	-2.22756100	0.36503600	0.90281000
N	0.67361200	0.52711300	0.72136100
N	2.24168300	-1.15561100	0.80869100
N	2.85158600	0.96672900	1.52702300
C	0.50505900	-1.22712100	-2.32724200
C	-0.57193100	-2.20519400	-2.22429500
C	-1.80752100	-1.42929600	-2.19999100
C	-1.49584600	-0.05018300	-2.51812400

C	-0.06209800	0.07017400	-2.58208900
C	1.95504800	-1.58463200	-2.39718200
H	2.19348700	-2.42428000	-1.73083700
H	2.59918000	-0.74129900	-2.11760100
H	2.22344800	-1.88725400	-3.42249400
C	-0.42168800	-3.54388600	-1.80124400
C	-3.18574400	-2.01328800	-2.16753000
H	-3.51750100	-2.27476700	-3.18563700
H	-3.91233500	-1.30285700	-1.74892700
H	-3.22014400	-2.93060900	-1.56350700
C	-2.47078600	0.99604400	-2.94413500
H	-2.11467400	2.00548800	-2.70126100
H	-3.45430100	0.85476500	-2.47888900
H	-2.60391800	0.93656200	-4.03666900
C	0.68954400	1.29638900	-2.98196900
H	0.70026700	1.38098700	-4.08065100
H	1.73272000	1.25750200	-2.64271100
H	0.22035300	2.20444700	-2.57980400
C	-3.17781800	-0.77437000	1.96606800
C	-3.39139100	-2.09686900	1.56279000
H	-2.97777600	-2.45088400	0.61617000
C	-4.13651700	-2.96495900	2.36191500
H	-4.29769400	-3.99588700	2.03938500
C	-4.67275800	-2.51488200	3.56692600
H	-5.25423100	-3.19412200	4.19440400
C	-4.46906400	-1.19403800	3.97157800
H	-4.89214600	-0.83785700	4.91344700
C	-3.72871800	-0.32497200	3.17418700
H	-3.58581000	0.71008800	3.49507000
C	-3.50168000	1.47682600	0.21741700
C	-4.79470900	1.00530600	-0.03834400
H	-5.07805000	-0.01148600	0.24376300
C	-5.73330100	1.83237100	-0.65478500
H	-6.73939900	1.45544200	-0.85156600
C	-5.39108000	3.13575500	-1.01327100
H	-6.12810800	3.78296300	-1.49394700
C	-4.10627500	3.61288700	-0.75199800
H	-3.83370800	4.63407400	-1.02740500
C	-3.16326000	2.78667300	-0.14475100
H	-2.15575400	3.16985200	0.03346500
C	-1.19199300	1.36998100	2.04866400
H	-1.72089100	2.26808500	2.39814700
H	-0.98685100	0.73162700	2.92186600
C	0.12198300	1.72157900	1.35571400
H	-0.04019400	2.50299800	0.59522100
H	0.81220100	2.13444700	2.10415400
C	1.89573800	0.13918900	1.00314300
C	3.52702300	-1.70824600	0.65431900
C	3.71702800	-3.03894100	1.04001000
H	2.88534400	-3.59248600	1.48273600
C	4.95137600	-3.65834100	0.85706600
H	5.07209300	-4.70075100	1.16380800
C	6.03433400	-2.97289700	0.29749500
C	5.82804500	-1.64121300	-0.08765700
H	6.65000100	-1.08123200	-0.54235300
C	4.59731000	-1.01287900	0.07337700
H	4.47413800	0.01588000	-0.26859000
C	7.37342500	-3.63114800	0.10069900
H	7.65760400	-3.64167500	-0.96347400

H	7.36805300	-4.67009500	0.45925600
H	8.16633400	-3.09030400	0.64103900
C	3.15881600	2.28544000	1.13684600
C	4.04787700	3.01939600	1.93308200
H	4.45595200	2.56780600	2.84106400
C	4.41221000	4.31093100	1.57095900
H	5.11022200	4.86231700	2.20712500
C	3.90142100	4.92043900	0.41601500
C	3.01800500	4.17339800	-0.36795800
H	2.60573400	4.61091700	-1.28147200
C	2.64887200	2.87339800	-0.02389000
H	1.96334100	2.32290100	-0.66751500
C	4.28788300	6.32864200	0.05063300
H	3.89645400	6.61021000	-0.93725800
H	5.38177100	6.45164200	0.03065800
H	3.89541700	7.05040500	0.78508800
H	3.57778800	0.50372300	2.06518100
H	-1.29618400	-4.19660800	-1.89662600
H	0.53731500	-4.02846100	-2.01294400
H	1.44424500	-1.80501400	0.78462200
O	-0.23466800	-2.43185600	0.55289700
H	-0.32746200	-3.13313800	-0.37769400
H	-0.80497900	-2.73037200	1.27571900
Ir	-0.68752800	-0.53632200	-0.58235200

TS_II^{Rh}-III^{Rh}

Rh	-0.73370300	-0.59057400	-0.65143100
P	-2.28616600	0.29166700	0.83082600
N	0.61905000	0.50396700	0.61938400
N	2.16972900	-1.18784200	0.71400900
N	2.79062600	0.92359800	1.46119300
C	0.43469900	-1.24135500	-2.40210800
C	-0.65304300	-2.20333700	-2.29670300
C	-1.87794500	-1.41777200	-2.26602900
C	-1.54998200	-0.04471400	-2.56544500
C	-0.11956200	0.05844100	-2.63405400
C	1.87966600	-1.61419400	-2.47526600
H	2.11058400	-2.46212400	-1.81698500
H	2.53418100	-0.77993200	-2.19298000
H	2.14086700	-1.91264600	-3.50374500
C	-0.51311600	-3.55392500	-1.89709400
C	-3.26131300	-1.98653700	-2.22506000
H	-3.60226700	-2.23230800	-3.24414800
H	-3.97830300	-1.27336600	-1.79518500
H	-3.30094700	-2.91090400	-1.63272000
C	-2.50855200	1.02690900	-2.96264100
H	-2.14742900	2.02307600	-2.67551400
H	-3.50254200	0.87729800	-2.52341800
H	-2.62070100	1.01310400	-4.05926700
C	0.63923900	1.29302100	-2.99074400
H	0.61026600	1.44259600	-4.08217900
H	1.69323000	1.22087500	-2.69366100
H	0.19922100	2.18385100	-2.52193300
C	-3.21147100	-0.86119300	1.90027900
C	-3.40060700	-2.18726000	1.49686500
H	-2.97585100	-2.53358200	0.55260900
C	-4.13055000	-3.06864500	2.29563600
H	-4.27314300	-4.10224800	1.97285900

C	-4.67437900	-2.62837700	3.50081000
H	-5.24398400	-3.31792000	4.12796100
C	-4.49301000	-1.30447200	3.90657800
H	-4.92162900	-0.95659100	4.84905500
C	-3.76864400	-0.42189500	3.10938800
H	-3.64345700	0.61550700	3.43036600
C	-3.58292200	1.39855700	0.17965100
C	-4.86241700	0.90225800	-0.10011500
H	-5.11959900	-0.13096200	0.14549100
C	-5.82086700	1.72468300	-0.69094600
H	-6.81562000	1.32771800	-0.90522500
C	-5.51300800	3.04895800	-1.00193700
H	-6.26539700	3.69257700	-1.46323400
C	-4.24337000	3.55117900	-0.71620600
H	-3.99792000	4.58895800	-0.95233500
C	-3.28059400	2.73004000	-0.13265700
H	-2.28698700	3.13628300	0.06958200
C	-1.24553000	1.30839700	1.96248600
H	-1.78508900	2.19288700	2.33053900
H	-1.01448000	0.66673100	2.82689200
C	0.05264900	1.68822800	1.25304700
H	-0.13406800	2.46415900	0.49167100
H	0.73902700	2.12332300	1.99327000
C	1.83204100	0.10949600	0.91599300
C	3.45115900	-1.75108800	0.57707100
C	3.62612700	-3.08389000	0.96412300
H	2.78446500	-3.63120800	1.39539800
C	4.85819200	-3.71264400	0.79822700
H	4.96655900	-4.75613400	1.10615100
C	5.95455900	-3.03535000	0.25493000
C	5.76390200	-1.70194500	-0.13221300
H	6.59663100	-1.14798700	-0.57466700
C	4.53560100	-1.06447900	0.01144600
H	4.42446300	-0.03471100	-0.33170100
C	7.29154400	-3.70365500	0.07777600
H	7.59188100	-3.71624800	-0.98193600
H	7.27291700	-4.74261100	0.43597200
H	8.08040500	-3.16911500	0.63022700
C	3.10714000	2.24807500	1.10271800
C	3.99233700	2.96055700	1.92212600
H	4.39205300	2.48663100	2.82246700
C	4.36540200	4.25857100	1.59231100
H	5.06111900	4.79167600	2.24619700
C	3.86711900	4.89616100	0.44779700
C	2.98842400	4.16963500	-0.36141100
H	2.58785600	4.62895800	-1.26949500
C	2.61101500	2.86409400	-0.05022800
H	1.93166800	2.32866400	-0.71290000
C	4.25113600	6.31482300	0.12220800
H	5.32403000	6.48917100	0.29343900
H	3.70133400	7.02849300	0.75774500
H	4.02634600	6.56277800	-0.92505200
H	3.50786700	0.44821500	2.00030400
H	-1.39641000	-4.19344200	-2.00344200
H	0.43368700	-4.04521600	-2.14647700
H	1.36234500	-1.82817000	0.67171500
O	-0.28978400	-2.47024800	0.44197500
H	-0.39913900	-3.18933600	-0.52062900
H	-0.82291700	-2.77419700	1.18938800

TS_IV^{Ir}-V^{Ir}

P	-2.23190600	0.42405400	0.83457200
N	0.68593900	0.54958400	0.71042000
N	2.29686300	-1.09355700	0.73395400
N	2.86018200	1.01935100	1.51410800
C	0.55561900	-1.22897900	-2.32148300
C	-0.47358100	-2.25012700	-2.18720400
C	-1.74474200	-1.53197500	-2.18664300
C	-1.49704100	-0.15339800	-2.54609500
C	-0.07114100	0.03571100	-2.60738000
C	2.01897000	-1.52183100	-2.41233100
H	2.30524700	-2.35736200	-1.76027000
H	2.63185000	-0.65474400	-2.13536800
H	2.28262200	-1.80212700	-3.44529800
C	-0.26518900	-3.56904000	-1.72569300
C	-3.09343500	-2.18087900	-2.16127700
H	-3.40110700	-2.46089100	-3.18204800
H	-3.85849900	-1.50549700	-1.75355400
H	-3.08764800	-3.09774700	-1.55571800
C	-2.52098100	0.82816300	-3.00833400
H	-2.21359700	1.86236600	-2.80903000
H	-3.49513700	0.66061000	-2.53257500
H	-2.65376500	0.71587300	-4.09674400
C	0.62135300	1.28369700	-3.04584000
H	0.64425600	1.32758900	-4.14684100
H	1.65964500	1.31411600	-2.69113600
H	0.09949300	2.17987400	-2.68378000
C	-3.29195600	-0.64376900	1.86487000
C	-3.57865900	-1.94687900	1.45019600
H	-3.12577700	-2.33367900	0.53620800
C	-4.42806600	-2.75503500	2.20576700
H	-4.64598600	-3.77280800	1.87468700
C	-4.99020000	-2.26491300	3.38319100
H	-5.65225900	-2.89828400	3.97807500
C	-4.70697900	-0.96363500	3.80355200
H	-5.14760700	-0.57690100	4.72512500
C	-3.86584800	-0.15278200	3.04572300
H	-3.66315400	0.86928900	3.37533300
C	-3.42243700	1.59765300	0.09525200
C	-4.75209800	1.22336500	-0.13130100
H	-5.11311700	0.24426700	0.19171900
C	-5.62906200	2.09825500	-0.77242700
H	-6.66379300	1.79467000	-0.94636900
C	-5.18939100	3.35530200	-1.18440900
H	-5.87826300	4.04056800	-1.68336900
C	-3.86725000	3.73692600	-0.95363300
H	-3.51661700	4.72118900	-1.27208000
C	-2.98619200	2.86129000	-0.32356300
H	-1.94932100	3.16945500	-0.17361000
C	-1.20291300	1.39431800	2.01440800
H	-1.72405900	2.29810400	2.36120800
H	-1.03109400	0.74185400	2.88469500
C	0.13215500	1.73538800	1.35927600
H	0.00478800	2.53998800	0.61647900
H	0.81003500	2.11881600	2.13409300
C	1.92091400	0.18432300	0.97102000
C	3.59517900	-1.61659400	0.58349200

C	3.80959800	-2.94978500	0.95099100
H	2.98757500	-3.52553500	1.38343300
C	5.05654100	-3.54078600	0.76818000
H	5.19703700	-4.58463400	1.06152900
C	6.13074600	-2.82416100	0.22768800
C	5.90177200	-1.49096000	-0.13421900
H	6.71614300	-0.90429800	-0.56795100
C	4.65548300	-0.89080800	0.02526500
H	4.51506300	0.14116800	-0.29968200
C	7.47190700	-3.47066600	0.00721900
H	7.63877800	-4.30194300	0.70741000
H	8.29173400	-2.74776100	0.12928100
H	7.54675100	-3.88049600	-1.01371100
C	3.13772000	2.35221200	1.14933000
C	3.98574500	3.10075200	1.97585400
H	4.38601900	2.64815100	2.88672400
C	4.31876600	4.40750000	1.63977200
H	4.98551700	4.97070400	2.29872600
C	3.81426400	5.01783300	0.48243700
C	2.97082900	4.25652300	-0.33132100
H	2.56471800	4.69585800	-1.24663500
C	2.63579500	2.94020500	-0.01471800
H	1.98170500	2.37704800	-0.68068000
C	4.16888100	6.44106000	0.14402700
H	3.77550000	6.73078100	-0.84068300
H	5.25985500	6.58907100	0.13094700
H	3.75754700	7.14088300	0.88922900
H	3.60285800	0.55869000	2.03146000
H	-1.11038400	-4.26171500	-1.80168800
H	0.71267200	-4.01817600	-1.92918500
H	1.50677900	-1.74888500	0.64795500
O	-0.15870600	-2.39428800	0.58370900
H	-0.19607800	-3.13478200	-0.32492000
C	-0.45770600	-2.90122900	1.86641000
H	0.41199300	-3.43555600	2.28731700
H	-0.71090000	-2.07214100	2.54357900
H	-1.31157200	-3.59728300	1.83635400
Ir	-0.66861400	-0.53669300	-0.59263000

TS_IV^{Rh}-V^{Rh}

Rh	0.70925900	-0.54580000	0.67282400
P	2.28804100	0.34429600	-0.77860200
N	-0.62925500	0.54720700	-0.61733000
N	-2.21269900	-1.11137100	-0.62191000
N	-2.79427400	0.97570700	-1.47319000
C	-0.47452600	-1.20289800	2.42167400
C	0.60380400	-2.17381000	2.30468800
C	1.83666600	-1.40070600	2.28627000
C	1.52096100	-0.02842000	2.59796100
C	0.09094300	0.08901000	2.66208800
C	-1.92174800	-1.56390900	2.51111700
H	-2.16496100	-2.42283500	1.87210900
H	-2.57529700	-0.73139300	2.22131800
H	-2.17370300	-1.84275800	3.54743400
C	0.44804000	-3.51963800	1.89445600
C	3.21573500	-1.98036700	2.25216000
H	3.55410800	-2.21548700	3.27462200
H	3.93819400	-1.27776000	1.81389600
H	3.24894900	-2.91197500	1.67123500
C	2.48874100	1.02643000	3.01640700
H	2.13883900	2.03168300	2.74816800
H	3.48213700	0.87474800	2.57712200
H	2.59755600	0.99035200	4.11290500
C	-0.65754300	1.32760000	3.02702200
H	-0.65218100	1.45421300	4.12178200
H	-1.70511800	1.27868200	2.70340200
H	-0.19352900	2.22108100	2.58747200
C	3.28643400	-0.78347200	-1.80522000
C	3.47068900	-2.10950100	-1.40478700
H	2.96830600	-2.47622400	-0.50817200
C	4.27219700	-2.96621600	-2.15975000
H	4.41008500	-4.00180300	-1.84109000
C	4.88737000	-2.50176600	-3.32082900
H	5.51280600	-3.17270900	-3.91419000
C	4.70362700	-1.17839300	-3.72820400
H	5.18481600	-0.81289500	-4.63810300
C	3.91049300	-0.31954600	-2.97193900
H	3.78335600	0.71853000	-3.29028500
C	3.53577200	1.49360500	-0.10001200
C	4.83084600	1.04353500	0.18767200
H	5.12852000	0.02204200	-0.06037400
C	5.75450500	1.89696400	0.78991400
H	6.76127900	1.53486700	1.00985000
C	5.39727200	3.20766400	1.10466900
H	6.12268200	3.87597800	1.57393200
C	4.11203800	3.66423900	0.81254900
H	3.82696900	4.69102300	1.05230700
C	3.18383500	2.81167400	0.21824200
H	2.17782700	3.18508100	0.01478200
C	1.25871400	1.32431700	-1.95219500
H	1.79774100	2.20505400	-2.33039100
H	1.05083500	0.66023100	-2.80569800
C	-0.05529500	1.71321100	-1.27795100
H	0.11164300	2.51421900	-0.53877300
H	-0.73117300	2.11995800	-2.04352500
C	-1.85166900	0.16607500	-0.89255400
C	-3.49609000	-1.66886200	-0.48437600
C	-3.63836200	-3.03546600	-0.74925200
H	-2.77077800	-3.60809300	-1.08761900

C	-4.86811100	-3.66602600	-0.57691200
H	-4.94974500	-4.73569700	-0.78781700
C	-5.99613700	-2.95746000	-0.15022000
C	-5.83753100	-1.59184800	0.11981700
H	-6.69468700	-1.01174100	0.47317800
C	-4.61119300	-0.95069300	-0.02893700
H	-4.52807600	0.10615500	0.22832900
C	-7.33397600	-3.62587600	0.01970200
H	-7.26849800	-4.70856300	-0.15835900
H	-8.07565000	-3.21377300	-0.68313700
H	-7.73098800	-3.47195400	1.03524600
C	-3.09328400	2.31199400	-1.14145600
C	-3.94680800	3.03054200	-1.98906300
H	-4.33577500	2.55250600	-2.89186100
C	-4.30062000	4.33949500	-1.68370700
H	-4.97144100	4.87829400	-2.35876500
C	-3.81296200	4.98235800	-0.53696000
C	-2.96420800	4.25085800	0.29844200
H	-2.57139200	4.71538400	1.20713700
C	-2.60777500	2.93321400	0.01266800
H	-1.95264900	2.39284200	0.69605500
C	-4.19042200	6.40734000	-0.23189000
H	-5.28298300	6.54308600	-0.24389900
H	-3.77145200	7.09890400	-0.98061700
H	-3.82107100	6.71943900	0.75536200
H	-3.52122400	0.49518800	-1.99452800
H	1.32703400	-4.16720200	1.98654200
H	-0.49940500	-4.00501700	2.15296800
H	-1.40904600	-1.74196700	-0.47994400
O	0.23297900	-2.43309700	-0.42474800
H	0.31727800	-3.16790800	0.52766400
C	0.36124700	-2.95019000	-1.72711100
H	-0.60551900	-3.33182100	-2.10577800
H	0.70551300	-2.16485400	-2.41786200
H	1.09234300	-3.77495600	-1.76224500

V^{Ir}

P	2.29506300	0.41040300	-0.87447200
N	-0.59118100	0.57598300	-0.87699500
N	-2.28265900	-0.99660300	-0.77658200
N	-2.77485100	1.11780700	-1.58409300
C	-0.51102200	-1.35799000	2.29267700
C	0.59381600	-2.33155400	2.13424700
C	1.79750600	-1.47943200	2.02826500
C	1.48070100	-0.18128900	2.55222700
C	0.02579400	-0.12466200	2.71065900
C	-1.94503100	-1.77028900	2.35463200
H	-2.18140500	-2.50881300	1.57653700
H	-2.62694500	-0.91920500	2.23386600
H	-2.15929700	-2.23866500	3.32971600
C	0.49784600	-3.67010300	1.97688400
C	3.17677400	-2.03573600	1.86015400
H	3.51421900	-2.52619700	2.78830200
H	3.90031100	-1.24757000	1.61201400
H	3.20743900	-2.78510300	1.05633700
C	2.43539100	0.84683600	3.06609600
H	2.05754200	1.86538500	2.90010100
H	3.41615900	0.76514900	2.58029200
H	2.58214100	0.71193700	4.15025700
C	-0.72829400	1.04128500	3.25913700
H	-0.71414700	1.00778700	4.36102600
H	-1.77797000	1.03008200	2.93663500
H	-0.27538300	1.99338800	2.94975300
C	3.22964000	-0.85543800	-1.80014400
C	2.81603400	-2.19144800	-1.74091600
H	1.96079900	-2.47164600	-1.12024400
C	3.47708700	-3.16467900	-2.49051500
H	3.14934800	-4.20520800	-2.43731800
C	4.55102500	-2.80920600	-3.30475200
H	5.07079500	-3.57254100	-3.88839200
C	4.95924800	-1.47600300	-3.37879500
H	5.79564000	-1.19364800	-4.02201900
C	4.30056100	-0.50123700	-2.63318700
H	4.62750900	0.53991200	-2.69862100
C	3.53382100	1.54877900	-0.17297700
C	4.80588900	1.10338200	0.20515800
H	5.11765100	0.07799400	-0.00567100
C	5.68559200	1.96669100	0.85787000
H	6.67651500	1.61032400	1.14788900
C	5.30252700	3.27740400	1.13912800
H	5.99305400	3.95212400	1.65002700
C	4.03530600	3.72610600	0.76451300
H	3.73020900	4.75208700	0.98192700
C	3.15299200	2.86594400	0.11514200
H	2.15716900	3.22493600	-0.15631800
C	1.35191000	1.32853900	-2.16732800
H	1.90320600	2.20672800	-2.53310200
H	1.22886200	0.62903100	-3.00802400
C	-0.01622800	1.70979200	-1.60786800
H	0.07533400	2.58096800	-0.93851500
H	-0.66717400	2.01547900	-2.43936400
C	-1.85457500	0.25332100	-1.06800000
C	-3.61315200	-1.39880900	-0.53357600
C	-4.00824800	-2.67877900	-0.93305200
H	-3.30391200	-3.31711200	-1.46970900
C	-5.28995400	-3.14208200	-0.64278800

H	-5.57356400	-4.14998000	-0.95745000
C	-6.21803000	-2.34446900	0.03473500
C	-5.80809000	-1.06257600	0.42616000
H	-6.50391400	-0.41808400	0.97034300
C	-4.52618600	-0.59312300	0.16075400
H	-4.23330000	0.39770200	0.51344900
C	-7.61318500	-2.83091200	0.31990900
H	-7.95334600	-2.51150000	1.31637900
H	-7.67492700	-3.92732000	0.27069400
H	-8.32937600	-2.42466700	-0.41326900
C	-2.93850600	2.47778100	-1.24452100
C	-3.70588900	3.28722600	-2.08971600
H	-4.13257200	2.86149100	-3.00132000
C	-3.92610500	4.62220900	-1.76975900
H	-4.53313900	5.23548400	-2.44137400
C	-3.38186500	5.19671700	-0.61270800
C	-2.61993900	4.37240800	0.22149500
H	-2.18940700	4.78460600	1.13819400
C	-2.40122200	3.02823800	-0.07711500
H	-1.80885800	2.41374100	0.60394200
C	-3.59785400	6.65278100	-0.29865300
H	-2.96875100	7.29169200	-0.94002600
H	-3.34611200	6.88094900	0.74686500
H	-4.64322900	6.94921700	-0.47239000
H	-3.57366100	0.68888600	-2.04237700
H	1.39097100	-4.28614100	1.84608400
H	-0.46585600	-4.18062100	2.05154100
H	-1.58639200	-1.75436800	-0.82030500
O	-0.59599600	-3.29076700	-0.89012400
H	-0.15885500	-3.33385300	-0.02178700
C	-0.73958000	-4.59272700	-1.40651600
H	-1.18757300	-4.51662100	-2.40730000
H	0.23462900	-5.10102400	-1.50966400
H	-1.39572400	-5.22106800	-0.77881600
Ir	0.69427500	-0.29407500	0.60612000

V^{Rh}

Rh	0.73584600	-0.35981200	0.65347100
P	2.36706200	0.33524100	-0.80411100
N	-0.53993800	0.46895800	-0.85980800
N	-2.23396600	-1.08682500	-0.74203400
N	-2.71224600	1.01379500	-1.61060200
C	-0.47501500	-1.31215000	2.36803100
C	0.69032200	-2.22108500	2.30153900
C	1.85338900	-1.31781100	2.21335400
C	1.43469500	-0.01100100	2.60339500
C	-0.02449200	-0.02216900	2.69036100
C	-1.88319800	-1.80640800	2.39918000
H	-2.04717000	-2.59444800	1.65092700
H	-2.61063000	-1.00550400	2.21750300
H	-2.10287400	-2.24338700	3.38746500
C	0.67541500	-3.57061100	2.21525500
C	3.26486300	-1.80649600	2.13640000
H	3.56794800	-2.26301400	3.09323400
H	3.96273700	-0.98718900	1.91761800
H	3.38030000	-2.56896800	1.35248500
C	2.30087300	1.12324900	3.04222800
H	1.88988100	2.09171500	2.72474700
H	3.31985700	1.03569300	2.64446100
H	2.36594500	1.13076800	4.14277300
C	-0.86347700	1.14237500	3.10074900
H	-0.90396600	1.20318300	4.20072600
H	-1.89344900	1.04723200	2.73214200
H	-0.44471000	2.08889300	2.73172200
C	3.34836300	-0.93176600	-1.67584800
C	2.99145200	-2.27783100	-1.53819200
H	2.15244800	-2.55055800	-0.89277700
C	3.68890100	-3.26379900	-2.23717500
H	3.40772200	-4.31313300	-2.12327300
C	4.74245500	-2.90930200	-3.07810800
H	5.29021600	-3.68155000	-3.62313000
C	5.09681200	-1.56667100	-3.22657800
H	5.91911200	-1.28681400	-3.88881500
C	4.40281400	-0.57998300	-2.53062500
H	4.68845700	0.46849900	-2.65064800
C	3.57556100	1.53179700	-0.14464700
C	4.84060300	1.11867900	0.29174700
H	5.16407200	0.08508700	0.14831600
C	5.70072500	2.02404900	0.91227300
H	6.68565800	1.69066400	1.24679200
C	5.30645200	3.34777700	1.10373100
H	5.98117500	4.05564700	1.59030300
C	4.04916700	3.76651700	0.66722900
H	3.73589800	4.80303700	0.81073600
C	3.18632700	2.86414300	0.04869600
H	2.20035200	3.20653000	-0.27523000
C	1.41681100	1.19881400	-2.12868300
H	1.96575600	2.06321800	-2.52991100
H	1.29624600	0.46661000	-2.94176400
C	0.04639200	1.59431100	-1.58624800
H	0.13918200	2.46679900	-0.91767600
H	-0.59165900	1.90682200	-2.42640500

C	-1.79747100	0.15530700	-1.05998500
C	-3.56129500	-1.49410600	-0.50553000
C	-3.92446400	-2.80230500	-0.84091600
H	-3.19443900	-3.45674000	-1.32214800
C	-5.20593800	-3.27259600	-0.56142100
H	-5.46370500	-4.30111700	-0.82798500
C	-6.16674200	-2.45552400	0.04328800
C	-5.78861700	-1.14720000	0.37451800
H	-6.51042400	-0.48634400	0.86243100
C	-4.50754600	-0.66912800	0.11911300
H	-4.24213300	0.34450300	0.42535800
C	-7.56195900	-2.94916900	0.31624200
H	-7.91444900	-2.62658100	1.30761600
H	-7.61611900	-4.04625000	0.27348200
H	-8.27349800	-2.55112700	-0.42578600
C	-2.90348200	2.36674300	-1.26261300
C	-3.70107800	3.16374400	-2.09264000
H	-4.12887100	2.73362500	-3.00176100
C	-3.95265800	4.49025400	-1.76012200
H	-4.58351200	5.09170800	-2.42048200
C	-3.41247100	5.07115900	-0.60427000
C	-2.61987200	4.26072600	0.21433100
H	-2.19124500	4.67617800	1.13065500
C	-2.36819100	2.92546200	-0.09783600
H	-1.75489400	2.32220900	0.57354400
C	-3.66474400	6.51811100	-0.27443800
H	-3.05371100	7.18008200	-0.90991000
H	-3.41588900	6.74137300	0.77294400
H	-4.71783100	6.78985700	-0.44195600
H	-3.50132500	0.57668900	-2.07747700
H	1.60531300	-4.14098600	2.15206000
H	-0.26096500	-4.13087300	2.27995400
H	-1.51395000	-1.82330900	-0.70301800
O	-0.34010300	-3.15193900	-0.65851200
H	0.05539800	-3.30627500	0.21661600
C	-0.30325400	-4.33390400	-1.42618400
H	-0.73326600	-4.11144500	-2.41260600
H	0.72913800	-4.69262700	-1.57708200
H	-0.89180900	-5.14462900	-0.96372200

11. References

[S1] (1) Carmona, M.; Ferrer, J.; Rodríguez, R.; Passarelli, V.; Lahoz, F. J.; García-Orduña, P.; Cañadillas-Delgado, L.; Carmona, D., *Chem. Eur. J.* **2019**, *25*, 13665-13670.

[S2] SAINT+, *version 6.01: Area-Detector Integration Software*, Bruker AXS, Madison, WI, **2001**.

[S3] (a) Blessing, R. H., *Acta Crystallogr*. **1995**, *A51*, 33-3. (b) SADABS, *Area Detector Absorption Correction Program*, Bruker AXS, Madison, WI, **1996**.

[S4] (a) Sheldrick, G. M., *Acta Crystallogr.* **1990**, *A46*, 467-473. (b) Sheldrick, G. M., *Acta Crystallogr.* **2008**, *A64*, 112-122.

[S5] Sheldrick, G. M., *Acta Crystallogr.* **2015**, *C71*, 3-8.

[S6] Farrugia, L. J., *Acta Crystallogr*. **2012**, *45*, 849-854.

[S7] Spek, A. L., *Acta Crystallogr. C* **2015**, *71*, 9-18.

[S8] Sheldrick, G. M., *CELL NOW and TWINABS.* University of Göttingen, Germany, **1995**.

[S9] Gaussian 09, Revision D.01, Frisch, M. J.; Trucks, G. W.; Schlegel, H. B.; Scuseria, G. E.; Robb, M. A.; Cheeseman, J. R.; Scalmani, G.; Barone, V.; Mennucci, B.; Petersson, G. A.; Nakatsuji, H.; Caricato, M.; Li, X.; Hratchian, H. P.; Izmaylov, A. F.; Bloino, J.; Zheng, G.; Sonnenberg, J. L.; Hada, M.; Ehara, M.; Toyota, K.; Fukuda, R.; Hasegawa, J.; Ishida, M.; Nakajima, T.; Honda, Y.; Kitao, O.; Nakai, H.; Vreven, T.; Montgomery, J. A.; Peralta, Jr. J. E.; Ogliaro, F.; Bearpark, M.; Heyd, J.

J.; Brothers, E.; Kudin, K. N.; Staroverov, V. N.; Kobayashi, R.; Normand, J.; Raghavachari, K.; Rendell,A. ; Burant, J. C.; Iyengar, S. S.; Tomasi, J.; Cossi, M.; Rega, N.; Millam, J. M.; Klene, M.; Knox, J. E.; Cross, J. B.; Bakken, V.; Adamo, C.; Jaramillo, J.; Gomperts, R.; Stratmann, R. E.; Yazyev, O.; Austin, A. J.; Cammi, R.; Pomelli, C.; Ochterski, J. W.; Martin, R. L.; Morokuma, K.; Zakrzewski, V. G.; Voth, G. A.; Salvador, P.; Dannenberg, J. J.; Dapprich, S.; Daniels, A. D.; Farkas, C.; Foresman, J. B.; Ortiz, J. V.; Cioslowski, J.; Fox, D. J., Gaussian, Inc., Wallingford CT, **2009**.

[S10] Chai, J.-D.; Head-Gordon, M., *Phys. Chem. Chem. Phys.* **2008***, 10*, 6615-6620.

[S11] Weigend, F.; Ahlrichs, R., *Phys. Chem. Chem. Phys*. **2005**, *7*, 3297-3305.

[S12] Marenich, A. V.; Cramer, C. J.; Truhlar, D. G., *J. Phys. Chem. B*, **2009**, *113*, 6378-6396.

[S13] Bryantsev, V. S.; Diallo, M. S.; Goddard, W. A. III, *J. Phys. Chem. B* **2008**, *112*, 9709-9719.
